# Structure, function and dynamics in acyl carrier proteins

**DOI:** 10.1371/journal.pone.0219435

**Published:** 2019-07-10

**Authors:** Rohit Farmer, Christopher Morton Thomas, Peter James Winn

**Affiliations:** 1 School of Biosciences, University of Birmingham, Edgbaston, Birmingham, United Kingdom; 2 Department of Computational Biology and Bioinformatics, Jacob Institute of Biotechnology and Bioengineering, Sam Higginbottom University of Agriculture, Technology and Sciences, Allahabad, India; 3 The Institute of Microbiology and Infection, University of Birmingham, Edgbaston, Birmingham, United Kingdom; 4 Centre for Computational Biology, University of Birmingham, Edgbaston, Birmingham, United Kingdom; Universidade Nova de Lisboa Instituto de Tecnologia Quimica e Biologica, PORTUGAL

## Abstract

Carrier proteins are four-helix bundles that covalently hold metabolites and secondary metabolites, such as fatty acids, polyketides and non-ribosomal peptides. These proteins mediate the production of many pharmaceutically important compounds including antibiotics and anticancer agents. Acyl carrier proteins (ACPs) can be found as part of a multi-domain polypeptide (Type I ACPs), or as part of a multiprotein complex (Type II). Here, the main focus is on ACP2 and ACP3, domains from the type I *trans*-AT polyketide synthase MmpA, which is a core component of the biosynthetic pathway of the antibiotic mupirocin. During molecular dynamics simulations of their apo, holo and acyl forms ACP2 and ACP3 both form a substrate-binding surface-groove. The substrates bound to this surface-groove have polar groups on their acyl chain exposed and forming hydrogen bonds with the solvent. Bulky hydrophobic residues in the GXDS motif common to all ACPs, and similar residues on helix III, appear to prohibit the formation of a deep tunnel in type I ACPs and type II ACPs from polyketide synthases. In contrast, the equivalent positions in ACPs from type II fatty acid synthases, which do form a deep solvent-excluded substrate-binding tunnel, have the small residue alanine. During simulation, ACP3 with mutations I61A L36A W44L forms a deep tunnel that can fully bury a saturated substrate in the core of the ACP, in contrast to the surface groove of the wild type ACP3. Similarly, in the ACP from *E*. *coli* fatty acid synthase, a type II ACP, mutations can change ligand binding from being inside a deep tunnel to being in a surface groove, thus demonstrating how changing a few residues can modify the possibilities for ligand binding.

## Introduction

Carrier proteins are essential for fatty acid, polyketide and non-ribosomal peptide synthesis, as well as other important biochemical pathways [1]. These proteins are important in biosynthetic pathways of interest for novel synthetic biology, particularly for the production of variants of pharmaceuticals such as the statins, or antibiotics such as mupirocin or daptomycin, amongst many.

**Fig 1:**
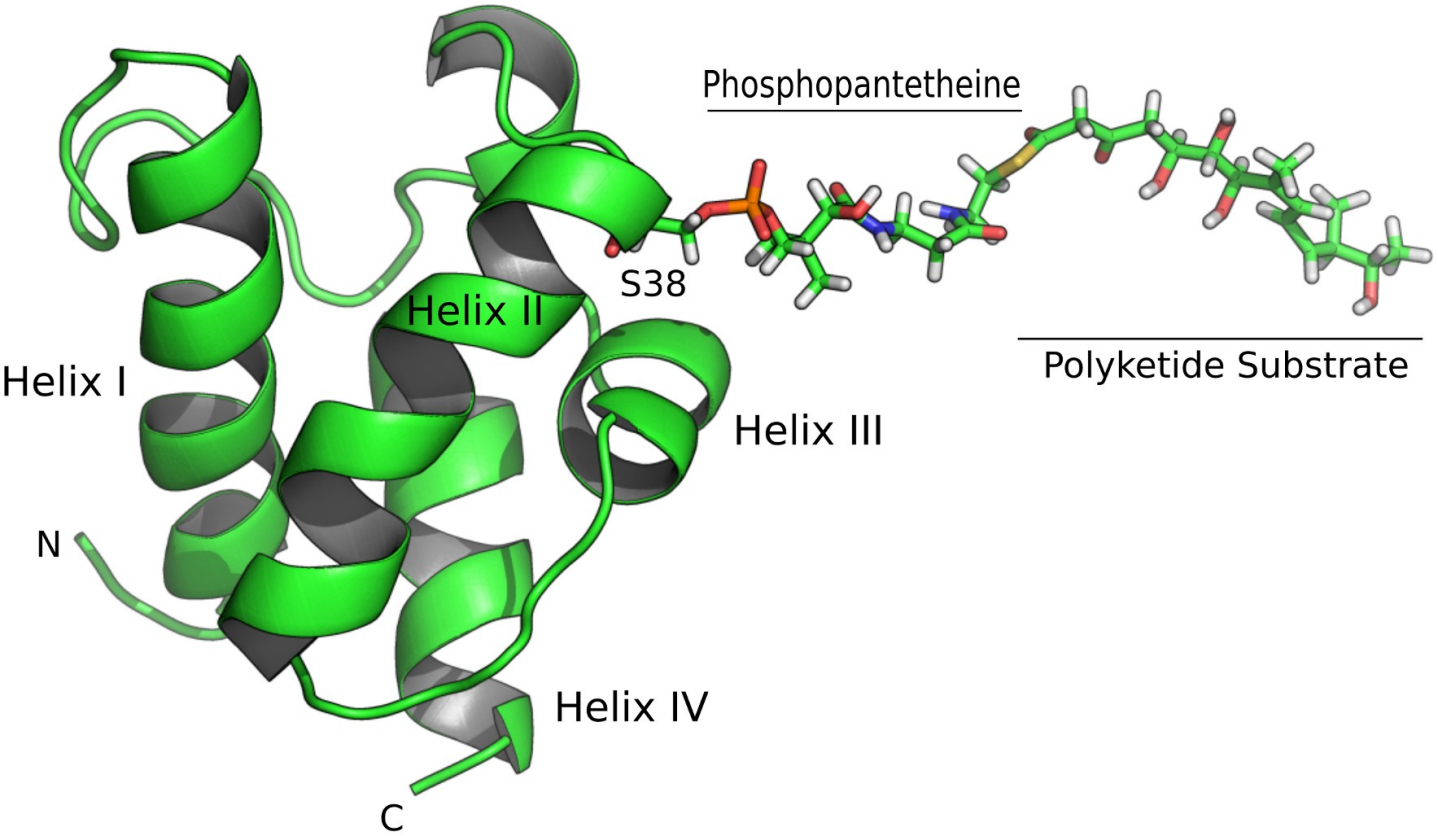
Cartoon drawing of acyl ACP3 from the mupirocin biosynthesis pathway. ACP3 is a four-helix bundle with S38 being unmodified in the apo form of the protein, phosphopantheine bound in the holo form, and with polyketide substrate attached to the phosphopantetheine in the acyl bound form. The protein backbone is shown as ribbon, with the S38 side chain and bound substrate shown as atoms with standard CPK colorings.

Carrier proteins comprise a four-helix bundle that is 70 to 100 amino acids in length. The holo form has a phosphopantetheine (ppt) arm attached to the catalytic serine, which is at the N-terminus of helix II (Fig 1). Acyl carrier proteins (ACPs) involved in fatty acid and polyketide synthesis, our main interest here, carry an acyl group attached to the ppt arm via a thioester linkage. Peptidyl carrier proteins (PCPs) carry an amino acid or peptide during non-ribosomal peptide synthesis. ACPs and PCPs can be classified as type I, where they form part of a multi-domain polypeptide chain, or type II where they are independent discrete proteins that interact with other discrete proteins. The substrate bound to an ACP or a PCP must be processed in a defined functional sequence by the numerous interacting enzymic domains that extend and modify it, thus ensuring that the correct product is produced.

The means by which the correct order of substrate processing is followed has been hypothesised variously in the literature to be due to partner proteins/domains: (i) recognising the substrate directly [2]; (ii) detecting the degree of burial of the acyl-phosphopantetheine in the ACP core; (iii) conformational changes in the ACP in response to the acyl chain [3,4]; or (iv) some combination of these mechanisms. How acyl chains interact with their ACP is thus of interest for those who wish to understand the specificity of processing in fatty acid synthase (FAS) and polyketide synthase (PKS) systems with the aim of improving the efficiency of substrate processing in re-engineered systems.

Acyl chains have been observed to bind to type II carrier proteins in several different ways, as outlined below. Acyl chain sequestration into a narrow internal tunnel, running between and parallel to helices II and IV, has been observed in three type II FAS-associated ACPs [4–6]. In contrast, the ACP from the pathway producing actinorhodin in *S*. *coelicolor* has a shallower acyl chain binding-groove, between helix II and III, which can open up to produce a cavity through the four-helix bundle, between helices III and II to helix IV, by the movement of helix III [7,8]. Helix II and helix III also bind the substrate in a type II peptidyl carrier protein, where pyrrole is seen to bind in a cleft that these helices form [9].

Unlike type II ACPs, type I ACPs show little evidence of acyl chain sequestration. The ACP from the PKS MLSA2, involved in mycolactone production, shows evidence of transitory interactions with hexanoyl and octanoyl substrates, with very shallow binding between the C-terminus of loop I, the N-terminus of helix II, and helix III, but not with substrates two or three carbons long [10]. Yeast FAS ACP shows strong substrate binding but is an atypical ACP structure with eight helices [11]. However, ligand binding to most type I ACPs observed to date causes only weak chemical shift perturbations on helices II and III, and no evidence of a stable interaction between the acyl chain and the ACP [2,12–14].

The current evidence thus points towards type II ACPs sequestering their substrates, possibly to protect them from solvent hydrolysis, and type I ACPs not sequestering their ACPs, possibly because, as part of a multi-domain modular system, they can transfer between processing domains fast enough to avoid significant rates of hydrolysis. One should however keep in mind that many of the experimental studies were performed with non-cognate acyl groups, a necessary experimental expediency. The results presented here focus on how substrates interact with type I ACPs from *trans*-AT systems, which are introduced below, and which we find bind their substrates in a similar way to type II ACPs.

AT refers to the acyl transferase domain/protein that loads an ACP with the chemical unit it uses to extend the growing polyketide chain. Type I modular PKSs can be subdivided into *trans*-AT systems, where the AT is a discrete protein separate from the main multi-domain polypeptide chain, and *cis*-AT systems, where the AT is part of the same polypeptide chain as the ACP. *Trans*-AT systems often have further proteins that act in *trans*, notably enoyl reductase proteins [15]. Thus, as well as acting with domains in the same polypeptide chain, like ACPs from *cis*-AT type I PKSs, ACPs in type I *trans*-AT PKSs interact with discrete proteins, and are thus similar to type II ACPs.

**Fig 2:**
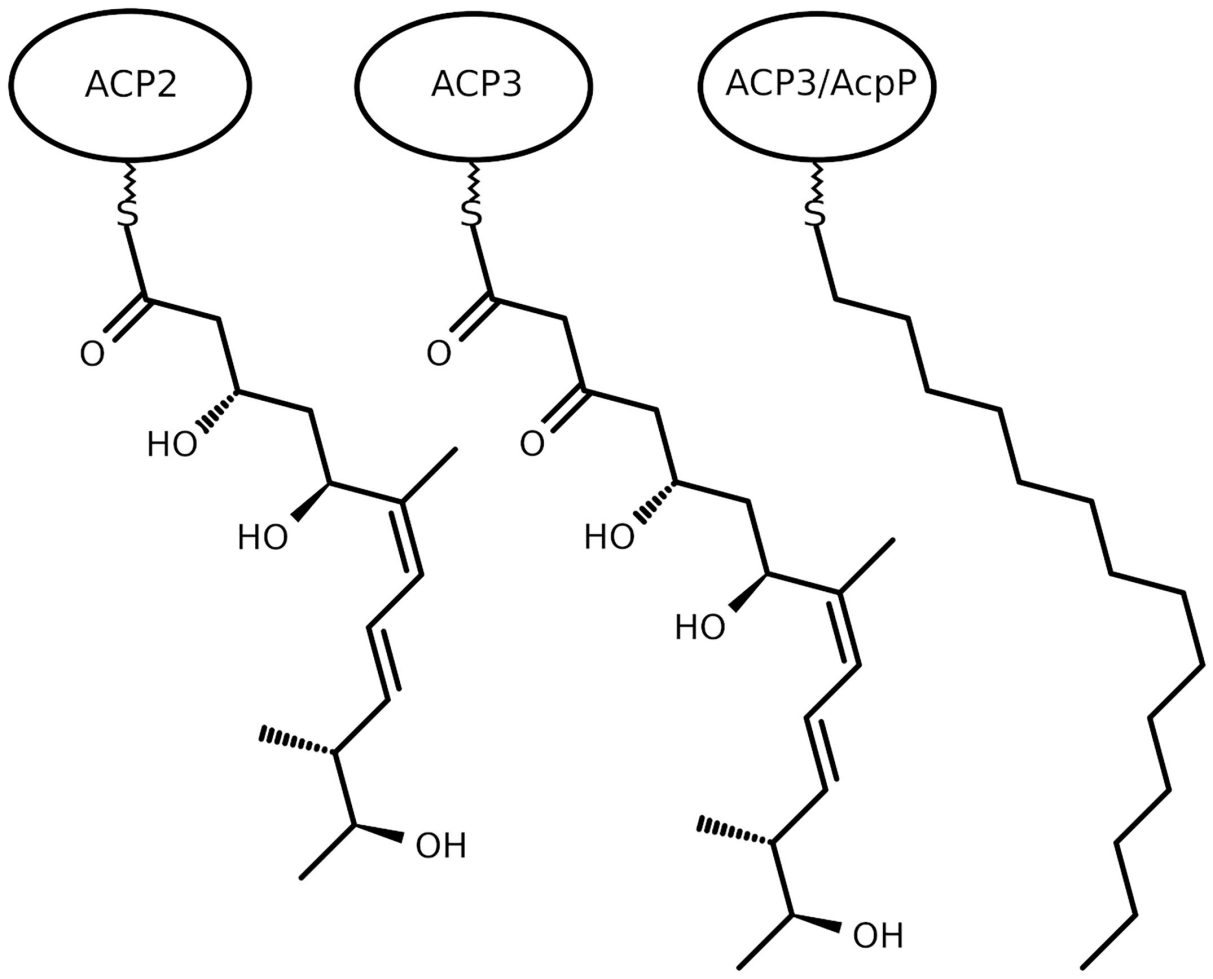
The structure of the cognate substrates that attach to ACP2 and ACP3 via a phosphopantetheine thioester linkage along with the structure of the fourteen carbon (14C) saturated chain attached to ACP3 or AcpP.

Here we investigate the second and third ACPs of MmpA, a multi-domain polypeptide from the mupirocin biosynthetic pathway, hereafter referred to as ACP2 and ACP3. ACP2 and ACP3 mediate the fifth and sixth steps of substrate elongation that occur during mupirocin biosynthesis. Their substrates are shown in Fig 2. The substrate of ACP2, once it has been fully processed, is passed over to ACP3 via a ketosynthase catalysed elongation reaction that is followed by further enzymic processing. ACP3 then passes the substrate down the biosynthetic machinery for further processing, as discussed elsewhere [16].

MmpA is a *trans*-AT type I PKS and thus, as argued above, can be classified somewhere between a classical type I and II PKS system. We find evidence that both ACP2 and ACP3 from this *trans*-AT type I PKS can sequester their cognate substrate to a surface groove, similar to the type II ACP from actinorhodin biosynthesis. Moreover, simulation of one of these *trans*-AT ACPs with three point mutations shows the formation of a tunnel somewhat similar to a type II FAS ACP, while simulation of a mutated ACP from a type II FAS can produce a more type I *trans*-AT PKS-like surface groove suggesting that critical differences can be determined by a few amino acid changes.

## Methods

### Molecular dynamics simulations

Simulations were performed as listed in Table 1. ACP2 and ACP3 were simulated with their cognate substrates attached (Fig 2). ACP3 was also simulated in holo and apo forms, and as a series of mutant forms, the latter to probe the amino acids responsible for substrate binding. ACP3 and AcpP, the ACP from *E*. *coli* FAS, and various mutants, were also simulated with a saturated 14 carbon chain attached to the ppt (Fig 2) via a thioether linkage. The rationale behind the choice of point mutations is discussed in the subsection “The GXDS motif and residue composition of helix III correlate with the mode of acyl chain sequestration” in the results section. ACP2 was modelled using Modeller [17] by homology to the PCP from *Acinetobacter baumannii* (PDB ID 4HKG), to which it has 36% sequence identity. The sequence of ACP2 is given in S1 Supporting Information File, alongside that of ACP3. The NMR structure of the apo ACP3 (PDB ID 2L22) and the crystal structure of the acylated FAS butyryl-AcpP from *Escherichia coli* (PDB ID 1L0I) were modified with PyMol, as noted in Table 1. Na^+^ ions neutralized the charge of each system in Table 1. An octahedron of TIP3P water [18] extending 1 nm beyond the protein surface was added by GROMACS [19,20].

**Table 1:** Summary of ACP simulations.

Structure	Ligand^1^	Number of Simulations
Apo ACP3 WT	-	1 X 1 μs
Apo ACP3 W44L	-	1 X 200 ns
Holo ACP3 WT	ppt	5 (4 X 50 ns; 1 X 200 ns)
Holo ACP3 W44L	ppt	5 (4 X 50 ns; 1 X 200 ns)
Acyl ACP3 WT	ppt + cognate substrate	5 (4 X 50 ns; 1 X 1 μs)
Acyl ACP3 W44L	ppt + cognate substrate	5 (4 X 50 ns; 1 X 200 ns)
14C ACP3 WT	ppt + 14 carbon saturated chain	3 (2 X 50 ns; 1 X 200 ns)
Acyl ACP2 WT	ppt + cognate substrate	3 (2 X 50 ns; 1 X 200 ns)
Acyl ACP3 L36A-I61A	ppt + cognate substrate	3 X 200 ns
Acyl ACP3 L36A- W44L-I61A	ppt + cognate substrate	3 (2 X 50 ns; 1 X 200 ns)
14C ACP3 L36A-I61A	ppt + 14 carbon saturated chain	3 (2 X 50 ns 1 X 200 ns)
14C ACP3 L36A-W44L-I61A	ppt + 14 carbon saturated chain	3 (1 X 50 ns; 2 X 200 ns)
14C AcpP WT	ppt + 14 carbon saturated chain	3 (2 X 50 ns; 1 X 200 ns)
14C AcpP A34L-A59I	ppt + 14 carbon saturated chain	3 (2 X 50 ns; 1 X 200 ns)
14C AcpP A34L-L42W-A59I	ppt + 14 carbon saturated chain	3 (2 X 50 ns; 1 X 200 ns)

^1^the different ligands are shown in Fig 2.

Simulations were performed with GROMACS and used the AMBER99SB-ILDN [21] and GAFF [22] parameters with missing charges for phosphopantetheine and substrates calculated using the RED server [23]. 1000 steps of conjugate gradient energy minimisation were followed by equilibration for 100 ps of NVT and 100 ps of NPT simulation with position restraints on all heavy atoms in the protein. Simulations used the V-rescale thermostat [24] and Parrinello-Rahman pressure coupling [25]. Simulations were carried out at 300 Kelvin and 1 atmosphere with a time step of 2 fs.

The changing cavity volume during simulations was calculated using *trj_cavity* plugin for GROMACS [26], with dimension of 5, grid spacing 1.3, cut off distance 9 and atomic radii from AMBER99SB-ILDN force field. A seed value is required by *trj_cavity*, for which we used the centre of mass of the cavity from the initial structure of each simulation, which was calculated using the PASS program from the MetaPocket server [27]. Further details are given in the S1 Supporting Information File. Modified forcefield parameters are available in the S1 Supporting Information File. Scripts, compressed simulation co-ordinates, and structures highlighted here can be downloaded from: https://github.com/rohitfarmer/acp-dynamics.

For analysis of the correlation between the GXDS motif and the residues present in helix III, sequence sets were manually verified for their context primarily on the basis of their title, type of organism and sequence starting number.

## Results

We simulated the structural dynamics of ACP2, ACP3, and AcpP, the ACP from *E*. *coli* FAS, a type II system. These were variously simulated in apo, holo and substrate bound forms, as described in Methods, Fig 2 and Table 1. To sample a wide range of conformations, multiple replicates for all the holo and acyl structures were simulated for 50 ns (Table 1). For each of these systems, the 50 ns simulation that showed the longest duration interaction between the phosphopantetheine and the surface of the ACP was extended to 200 ns duration. The 200 ns simulations of Apo ACP3 WT and Acyl ACP3 WT were extended further to 1 μs duration. For each simulation, how the root mean squared deviation (RMSD) of the backbone atoms from the starting configuration varies during the simulation is presented in the S1 Supporting Information File. The root mean squared fluctuation (RMSF) of the backbone atoms in selected simulations and the change in cavity size during each simulation are also given in the S1 Supporting Information File.

### Phosphopantetheine is sequestered to a groove on the surface of ACP3

The long apo and holo simulations of WT ACP3 (1 μs and 200 ns duration, respectively) show similar behaviour, but the holo simulation had maximum and modal cavity volumes that were 1.5 and 2.1 times those in the apo simulation (Table A in S1 Supporting Information File). Both simulations have little fluctuation in the core residues of helix I, the N-terminus of helix IV, and parts of helix II, but the N-terminii of helices II and III show similar levels of fluctuation to the inter-helical loops (Figs E, F in S1 Supporting Information File). The average backbone RMSD over all the frames of the trajectory from the starting NMR structure is approximately 2.2 Å for both simulations (Figs C, D in S1 Supporting Information File), indicating no major changes in conformation.

**Fig 3:**
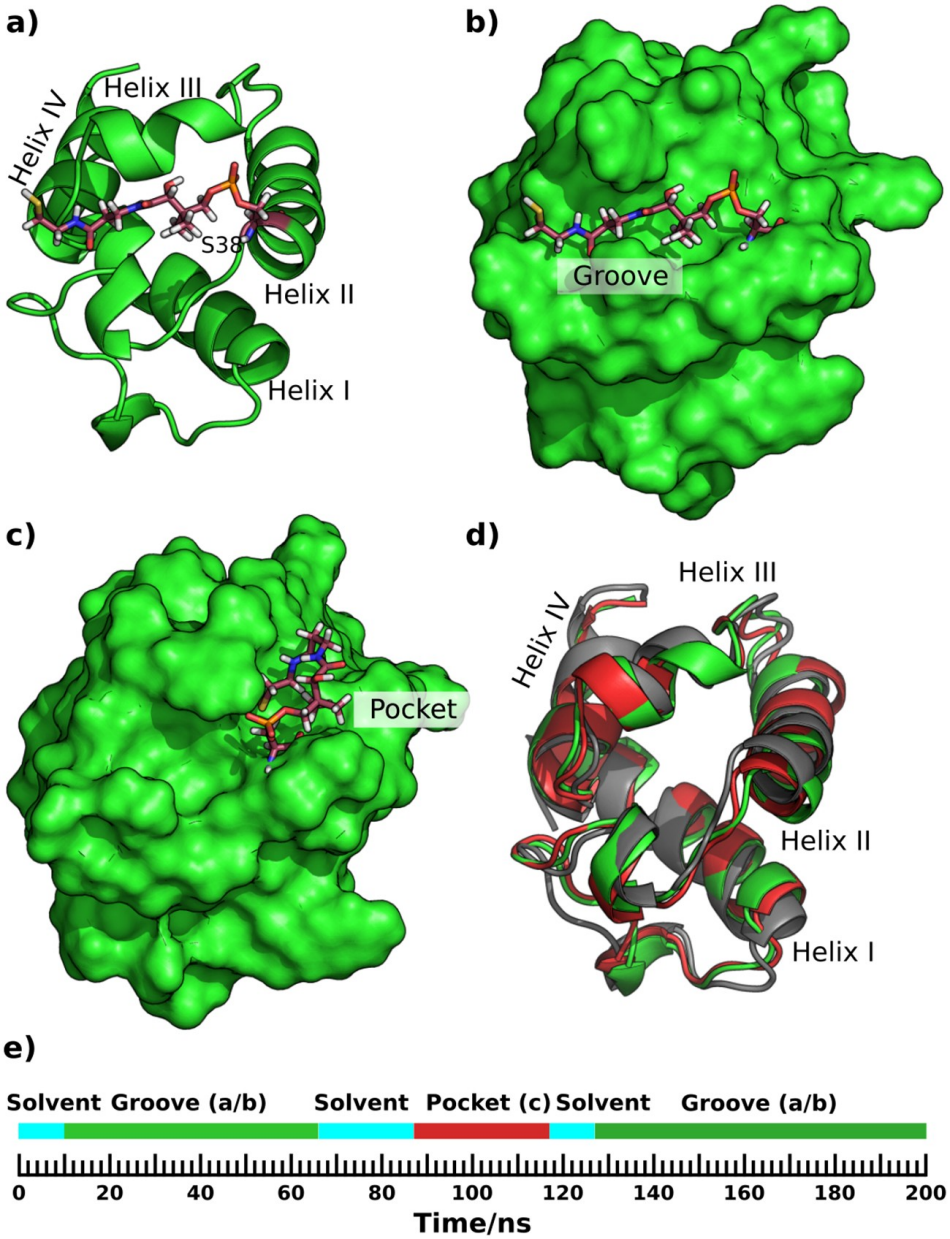
Substrate-bound conformations during a 200 ns simulation of holo ACP3. a) & b) Cartoon & surface representations with the phosphopantetheine (stick representation) lying flat in a groove bounded by helix III, part of the loop connecting helix I and II (R30, F31, L32, E33, L34, G35, L36, D37; numbering is as PDB id 2L22, Fig E in S1 Supporting Information File) and the N-terminus of helix II. c) Phosphopantetheine diving into a small pocket formed by the N-terminus of helix II and helix III. d) Variation in helix position during the simulation; starting position in grey, snapshot shown in a) & b) in green and c) in red. e) Timeline of the simulation showing the occurrence and duration of the different conformations. The letters a, b, and c accord with the Figure panel labels. The orientation is the same in all the sub-figures. A version of this figure with transparent surface is Fig W in S1 Supporting Information File. The co-ordinates of the structures used to generate this and subsequent figures are available for download at https://github.com/rohitfarmer/acp-dynamics.

During the simulation the phosphopantetheine transitioned between solvent, a groove on the ACP’s surface (Fig 3a and 3b), and a small vertical pocket (Fig 3c), as indicated in Fig 3e. The different phosphopantetheine binding configurations correlate with only minor changes in the position of the helices and loops of ACPs (Fig 3d).

### Cognate substrates of ACP3 and ACP2 can bind to the surface groove between helices II and III, but also remain in solvent

Of five 50 ns simulations of ACP3 with cognate substrate (Fig 2), in only one did the acyl chain bind to the groove seen in the holo-ACP simulations, between helices II and III. During two simulations the ppt moiety bound to the same groove but the acyl chain was fully solvent exposed. During the other two simulations there was no binding to the groove; in one the acyl chain remained in contact with the surface in between helix II and the loop connecting helices II and III and in the other the acyl chain passed over helix III and remained on the surface.

**Fig 4:**
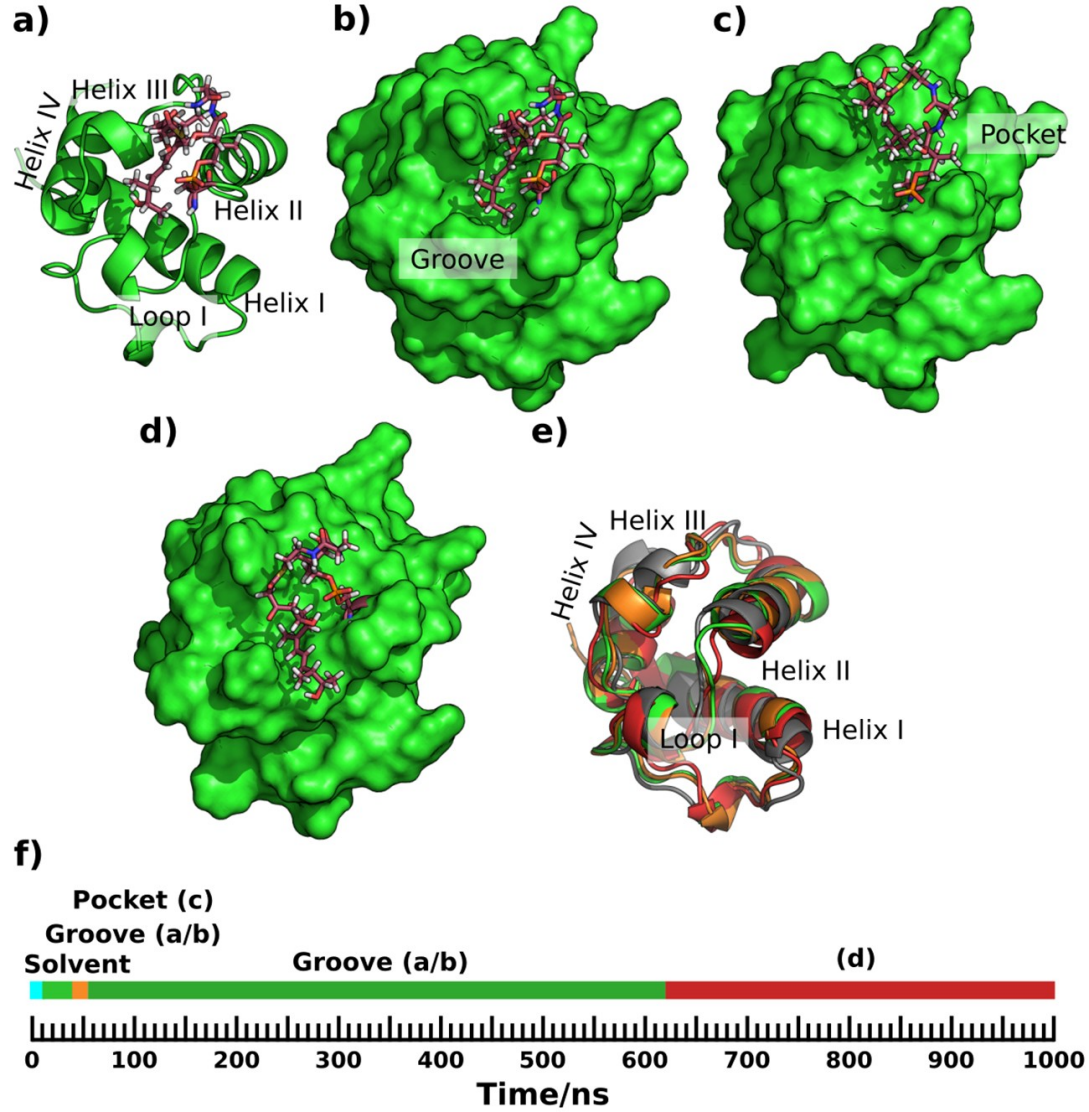
Cognate substrate binding to the surface groove during a 1 μs simulation of acyl ACP3 WT. a) & b) Cartoon & surface drawing respectively of the acyl ACP3 WT with the acyl chain lying flat in the groove—residues within 5 Å of the acyl chain and ppt are F31, L32, L36, D37, V39, I40, A41, A42, A58, A59, G60, Y62 and P65. c) Acyl chain in a small pocket formed by the N-terminus of helix II and helix III. d) Acyl chain passing over loop I. e) Comparison of structural variation; starting position in grey, configuration a/b in green, c in orange and d in red. f) Timeline of the simulation. The orientation of all sub-figures is equivalent and they appear in the same order as in the 1 μs trajectory. A version of this figure with transparent surface is Fig X in S1 Supporting Information File.

The simulation with the acyl chain lying in the groove between helices II and III was extended to 1 μs duration. The phosphopantetheine was found to be solvated for almost all the simulation. The mean and modal cavity sizes were 82.5 and 72.5 Å^3^ respectively. The acyl chain enters a groove similar to that seen in the holo-ACP simulation (compare Fig 4a and 4b and Fig 3). It briefly enters the pocket conformation (Fig 4c) and a variant of the groove-bound conformation with the acyl chain alpha carbon in the groove, but the latter part of the chain passing over loop I into the solvent (Fig 4d).

We simulated another *trans*-AT ACP, a homology model of ACP2 with its cognate substrate (Fig 2), there being no other experimentally determined structure of a *trans*-AT ACP available. Quality metrics for the model are given in the Results section of the S1 Supporting Information File, with e.g. the Ramachandran plot having only one residue (excluding glycine and proline) in a disallowed region (Fig B in S1 Supporting Information File). Of three 50 ns simulations one bound the acyl chain, which was subsequently extended to 200 ns duration. The acyl chain bound to the surface groove at ~15 ns where it remained to the end of this 200 ns simulation. The maximal, mean and modal cavity sizes were similar to those in the holo and acyl WT ACP3 simulations (Table A in S1 Supporting Information File). For the other two 50 ns simulations of ACP2, only the ppt portion of the ligand was bound to the surface, with the polyketide chain in the solvent, an interaction that lasted for approximately half of each simulation.

### The binding mode of the WT ACPs is independent of substrate

There is a weak correlation between cavity size and FAS-likeness, with ~-0.2 Pearson’s correlation between the cavity volume and the backbone RMSD of the ACPs from the FAS AcpP reference structure (Table B & Figs M-R in S1 Supporting Information File). This suggested the possibility that ACP3 may have the capacity to bind substrates in a similar fashion to a type II FAS, but that the cognate substrates were too hydrophilic for such deep burial.

Three 50 ns duration trajectories of ACP3 were simulated with a 14C saturated chain (Fig 2), a hydrophobic chain with backbone equivalent in length to the cognate substrate. This latter construct was to test the effect of an extended extremely hydrophobic ligand, i.e. a pure carbon chain. Such thioether constructs have been previously used to increase the chemical stability of the substrate-ACP covalent interaction during experimental work [6]. In one simulation the carbon chain bound in the surface groove, which simulation was subsequently extended to 200 ns duration. The other two simulations had either unbound solvent-exposed ligand or only the ppt moiety of the ligand bound to the groove with the saturated carbon chain moiety hanging in the solvent.

During the first 89 ns of the 200 ns simulation the ligand was bound to the surface groove and ACP3 became more like an ACP from a type II FAS; the backbone RMSD from AcpP fluctuating between 1.5 and 2Å (Fig P d in S1 Supporting Information File), and the ligand-binding cavity becoming larger. The correlation between the RMSD from AcpP and the cavity size was -0.5, which suggests an association between having an FAS backbone conformation and the capacity to form a large cavity. Subsequently, the ligand bound to a narrow channel parallel to helix II and the loop connecting helices II and III (Fig T in S1 Supporting Information File) and the backbone RMSD from AcpP progressively increased up to almost 3 Å. The saturated carbon chain never entered the core of the ACP.

**Fig 5:**
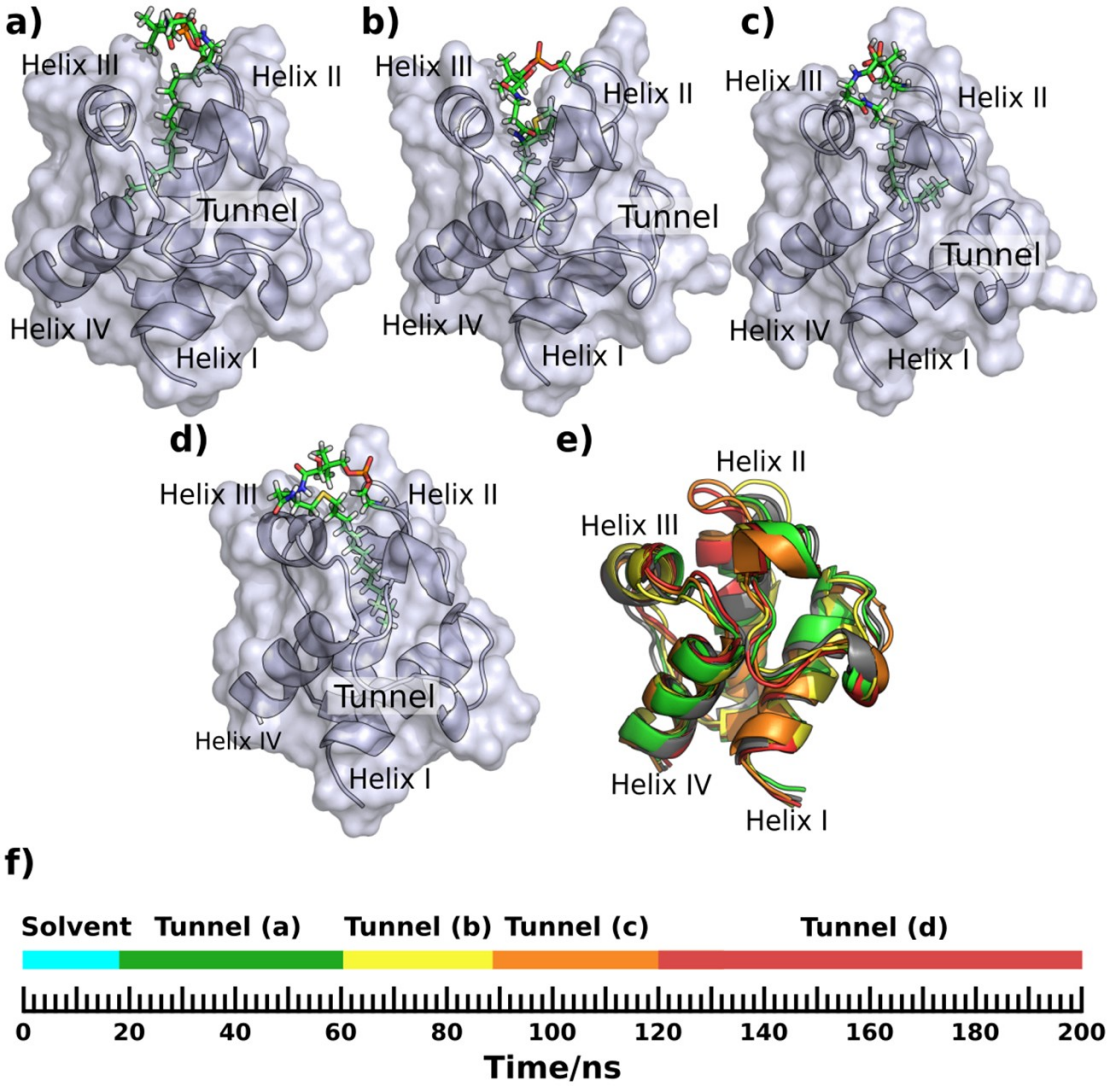
Sequestering of a 14C saturated chain by WT AcpP. The phosphopantetheine-ligated ligand (stick representation) penetrates the surface (transparent rendering) at the opening formed by the N-terminus of helix II, helix III and loop I (cartoon drawing). The 14C chain broadly orients itself in four different orientations, a), b), c) and d), described in the main text. The conformation shown in d occurs at 120 ns with the extent of burial of the ligand fluctuating during the rest of the simulation (Fig V e, f in S1 Supporting Information File). Superposed in e) are the starting position in grey, the orientation from panel a in green, b in yellow, c in orange and d in red. f) Timeline of the simulation with color scheme as in e.

For comparison with the ACP3 simulations, simulations of WT AcpP with the 14C saturated, thioether-linked substrate (Table 1), showed the carbon chain burying itself in the core of the protein and several different bound conformations with different cavity volumes (Fig 5). The conformational diversity observed here, to our knowledge, has not been observed before. We can’t rule out that this diversity is due the thioether lacking the carbonyl of the thioester, although previous simulations with acyl chains did not report a role for the carbonyl in orientating the chain [28]. These AcpP simulations demonstrated that our simulation protocols and parameters can broadly reproduce results seen in X-ray crystallography data and in previous simulation work [4,28,29], thus providing a control.

Out of the three 50 ns long simulations of AcpP one simulation formed a narrow deep tunnel, similar to observations reported by Chan *et al* [28]; the same simulation was further extended to 200 ns. However, several binding conformations were seen: the 14C acyl chain bent towards helices III and IV (Fig 5a); the alpha to gamma carbons of the 14C acyl chain curled with the remaining length of the ligand in a staggered conformation running parallel to helix II (Fig 5b), this conformation also recording the maximum cavity volume (Table A in S1 Supporting Information File); the 14C chain bent towards helix II (Fig 5c); a staggered conformation along the full chain length, running parallel to helix II (Fig 5d), completely buried but with a much lower cavity volume as compared with that in Fig 5b. Superimposing examples of these states shows changes in the position of helices, notably helix III and the N-terminus of helix II, presumably depending upon the burial of the ligand (Fig 5e). In the other two simulations the ligand stayed solvent exposed for the whole 50 ns, occasionally touching the surface in different places.

### The GXDS motif and residue composition of helix III correlate with the mode of acyl chain sequestration

**Fig 6:**
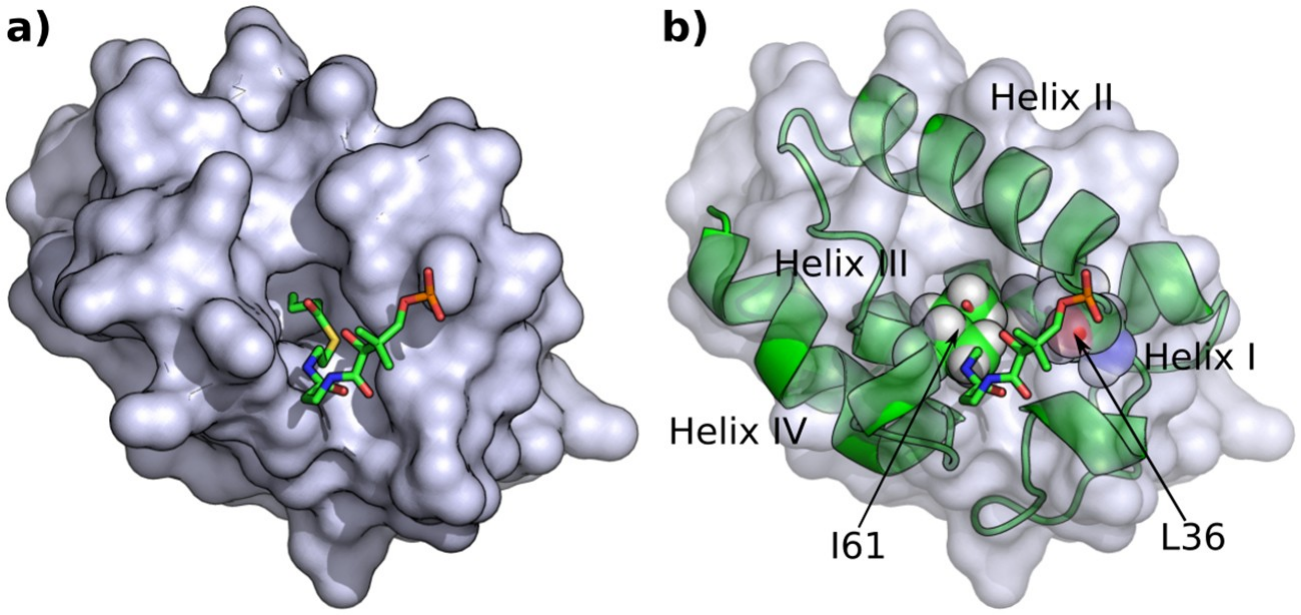
Comparison of FAS AcpP and ACP3 structures. a) Surface rendering of AcpP with the ligand (shown as sticks) buried in the cavity. b) Superimposed AcpP (surface) and ACP3 (cartoon) structures with the I61 & L36 (spheres) shown as a blockage at the cavity opening. A version of this figure with transparent surface is Fig Y in S1 Supporting Information File.

**Fig 7:**
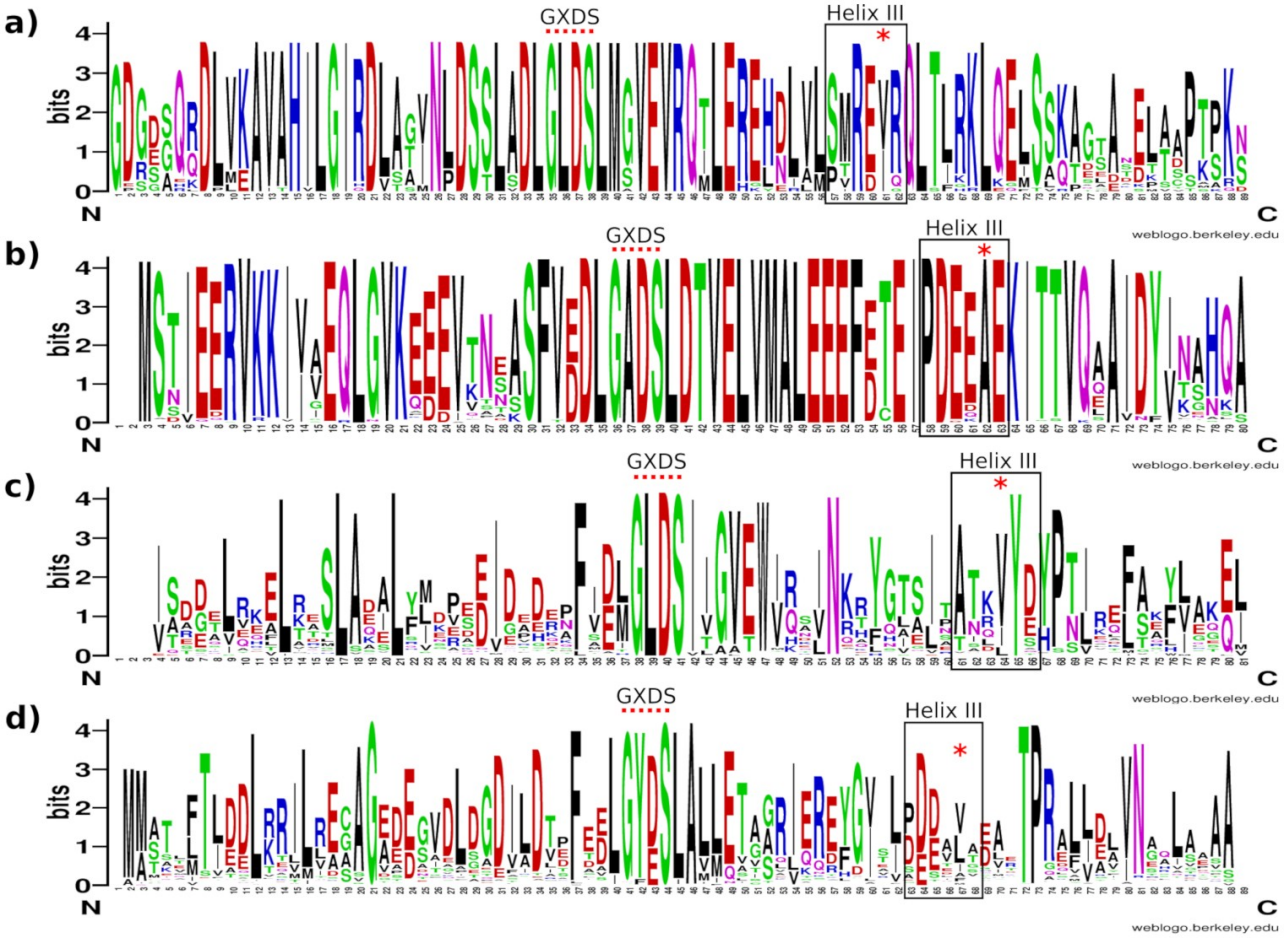
The correlation between a bulky X residue in the GXDS motif and the residue of helix III that packs into the protein core. The residue equivalent to AcpP:A59, which packs into the protein core, is indicated by *. The length of the sequence logo is that of the alignment including gaps, which does not correspond to the length of the template sequence; thus the numbering does not correspond to that used in the text, which follows that of the PDB entries. Empty positions in the logo correspond to too few residues in the sequence alignment for logo construction. Sequence logos were built on a) 27 unique type I ACP sequences from a PHI blast search with a GLDS motif and the rat FAS sequence. Searches were restricted to mammals and fungi and results are all part of larger multi-domain proteins. b) 316 unique type II ACP sequences from a PHI blast search with a GADS motif and the AcpP sequence. The search was restricted to bacterial sequences and produced only discrete ACP sequences. c) 101 unique type I ACP sequences from a PHI blast search with a GLDS motif and the ACP3 sequence. The search was restricted to bacterial sequences and returned ACPs from multi-domain contexts. d) 449 unique type II ACP sequences from a blast search with the actinorhodin ACP sequence. The search was restricted to actinomycetes only and produced type II-like discrete sequences.

Aligning the structures of AcpP and ACP3 indicates that L36 and I61 of ACP3 block the space where the substrate-sequestering channel is seen in AcpP (Fig 6). L36 corresponds to residue X of the GXDS motif, common to all ACPs, the S being the ppt binding site at the N-terminus of helix II (Fig 1). I61 corresponds to the residue in helix III that anchors into the core of the four helices of the ACP, i.e. pointing towards the region where the phosphopantetheine linked fatty acid binds in AcpP. I61 interacts with residues from helices II and IV and loop I whereas Y64, the only other residue from helix III that has any buried side chain, interacts only with helices III and IV and points towards the solvent rather than the core of the protein. Sequence and structure analysis indicates that residues equivalent to L36 and I61 are generally bulky residues in type I ACPs (Fig 7a and 7c), but alanine residues in type II FAS-like ACPs (Fig 7b), e.g. A34 and A59 in AcpP. In type II PKS sequences these are again bulky, Y40 and A65 in actinorhodin ACP but the A65 position is more typically a long chain aliphatic residue (Fig 7d). The backbone of the X residue of the GXDS motif superposes well in all four structures, but the side chains have different orientations (Fig U in S1 Supporting Information File).

Despite ACP3:I61, AcpP:A59 and ActACP:A65 all functioning to anchor their respective helix III to the core of their ACP, these residues do not spatially overlap in their superposed structures (Fig U in S1 Supporting Information File), since the positioning of helix III varies. ACP3, ActACP, and the ACP from rat FAS nonetheless have aliphatic side chains occupying space in this region where AcpP has none (Fig U in S1 Supporting Information File), AcpP being the only protein to sequester its substrate into its core. Despite no exact correspondence between sequence position and residue location in the three dimensional structure, the presence of either bulky hydrophobic residues or alanine within the different ACP types points towards those residues’ possible functional importance, possibly assisting the orientation of helix III and thus modifying substrate-binding properties and dynamics.

The above observations suggest that a few mutations might be sufficient to confer AcpP-like substrate sequestering properties on ACP3, and vice versa. We therefore performed simulations of substrate binding for AcpP and ACP3 with respective double mutations: A34L, A59I, and L36A, I61A (Table 1). We also incorporated W44L in ACP3 (Fig U in S1 Supporting Information File), since this position had previously been hypothesised to be important for orienting helix III of ACP3 [16], helix III seemingly important for substrate binding properties. The three replicates of Acyl ACP3 L36A-I61A and two replicates of Acyl 14C ACP3 L36A-W44L-I61A were all simulated for 200 ns.

**Fig 8:**
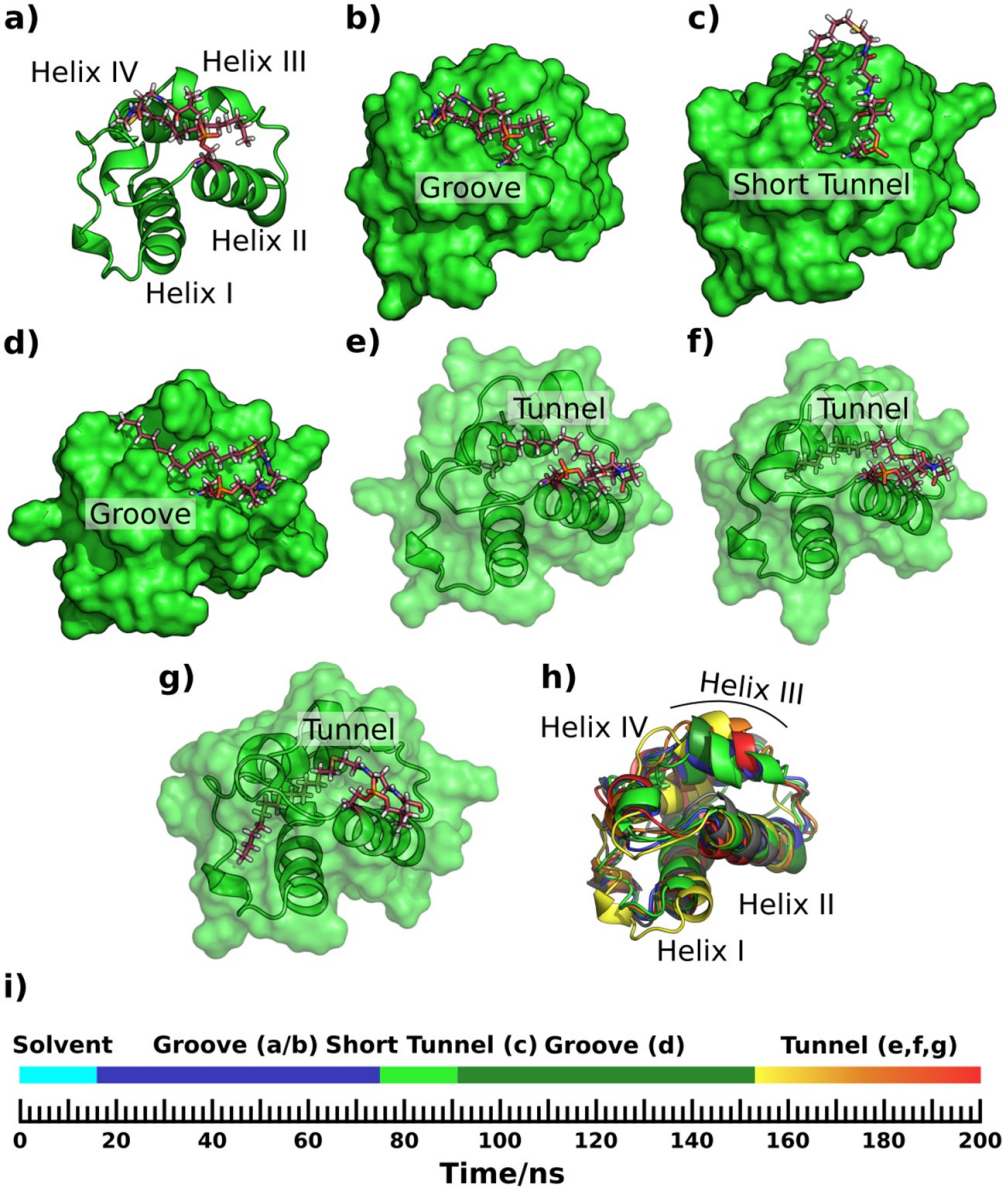
Simulation snapshots showing sequestering of a 14C saturated chain by ACP3 L36A-W44L-I61A. Groove and pocket structures similar to the apo and holo WT ACP3 simulations, a), b), c), d), are followed by progressive penetration of the carbon chain into a tunnel perpendicular to helix II e), f) & g). Superposition of the different configurations h), with the starting structure in grey, and other colors consistent with the timeline, i).

A short tunnel did form in the early part of a 200 ns simulation of ACP3 L36A-W44L-I61A with the hydrophobic 14C substrate bound (Fig 8c; Fig V b in S1 Supporting Information File), but only a short section of substrate was bound, namely almost all of the terminal methyl group and one side of the preceding four methyl groups. This tunnel was bounded by helix III, the loop C-terminal to it, the loop N-terminal to helix II and the N-terminal part of helix II, and was similar to the start of the AcpP binding tunnel. A much larger tunnel had formed by the end of that simulation, but the carbon chain orientation was perpendicular to the N-terminus of helix II (Fig 8e, 8f and 8g) in contrast to the parallel orientation in WT AcpP (Fig 5; Fig V e, f in S1 Supporting Information File). The start of the long tunnel is between helix III, the N-terminal part of helix II and the loop N-terminal to helix II. The ligand passes through this and then aligns along and almost parallel to the mid-point of helix I, with the tail of the ligand crossing the C-terminus of helix I almost perpendicular to it, where it emerges back into the solvent (Fig 8g). It seems that the internal cavity formation is the result of the deepening of the surface groove (Fig 8d) followed by it being covered by the loop between helices I and II. At the end of the simulation the 14C chain was completely buried in the tunnel but the terminal carbon was visible from the other side of the protein surface (Fig 8g). In the 50 ns simulation of this system (Table 1) the acyl chain remained solvent-exposed and located close to helix II throughout the simulation, whereas in the other 200 ns simulation the acyl chain remained in a groove-bound conformation similar to Fig 8b.

Mutants L36A-I61A and L36A-W44L-I61A of ACP3, with the cognate substrate bound (rows 9 and 10 in Table 1) formed shallow surface grooves but nothing similar to the AcpP substrate binding channel.

For AcpP to be more ACP3-like also required a triple mutation, A34L-L42W-A59I, with the double mutant having a similar substrate binding mode to wild type, albeit with a reduced maximal, mean and modal cavity volume. Of three 50 ns simulations of 14CAcpP A34L-L42W-A59I triple mutant, two failed to form a deep tunnel and instead formed a surface groove similar to WT ACP3, either binding only the ppt portion of the ligand or not binding the ligand at all. The third simulation did form a short tunnel, as described further below, but the ligand did not settle stably inside it during the simulation.

**Fig 9:**
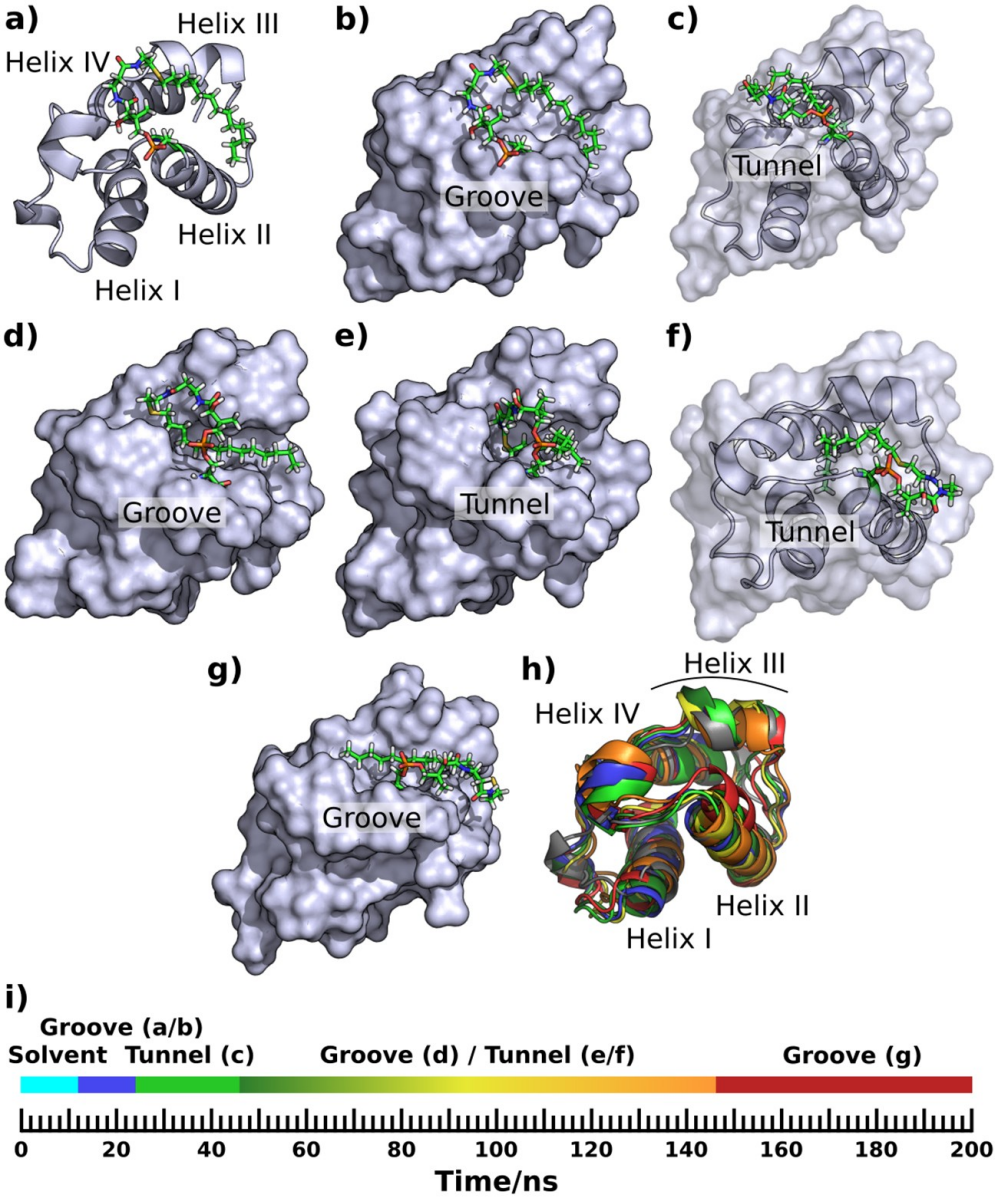
Sequestering of a 14C saturated chain by AcpP A34L-L42W-A59I. a) & b) cartoon & surface drawings respectively of the carbon chain lying flat in the surface groove parallel to helix II. c), d), e), f) & g) show tunnel and groove bound conformations described in the main text. The different conformations are superposed in h), the starting conformation colored grey, the others colored consistent with the timeline, i); the middle section of the simulation oscillates between the configurations in d (green), e (yellow) and f (orange).

Extending the third simulation to 200 ns saw oscillation between the short tunnel and surface-groove ligand-binding sites. Initially the ligand attached in a groove parallel to, and in between helix II & helix III (Fig 9a and 9b), effectively an expansion of the groove seen in WT ACP3. A tunnel started to form, parallel to and between helices II and IV and also bounded by helix III and the loop N-terminal to helix III (Fig 9c), but this changed into a deep surface groove with the ligand lying flat within it (Fig 9d). The cavity then oscillated between the tunnel and surface groove with the ligand staying bound for ~100 ns. A maximum burial of only half of the ligand was seen during the simulation (Fig 9f), as compared to the burial of almost the entire ligand in WT AcpP simulations (Fig 7). Further conformations seen in the 200 ns simulation had the ligand curling towards the catalytic serine (Fig 9e) or lying flat in a surface groove similar to WT ACP3 simulations (Fig 9g). Thus, substituting three residues in AcpP with those of ACP3 severely hampered its substrate sequestering capability, making it more ACP3 like.

Of three 50 ns simulations of 14C AcpP A34L-A59I, the simulation that saw ligand binding and cavity formation ~30 ns after the start was extended to 200 ns duration. Once entering the cavity, the ligand stayed inside till the end of the simulation, as in the WT AcpP simulations. The ligand orientations were also broadly similar to the AcpP WT simulation. In the other two simulations the ligand bound to the surface but remained solvent-exposed, either parallel to helix II or in a small surface groove formed by the loop between helix I and helix II.

## Discussion

In our simulations, ACP2 and ACP3 can sequester their substrates to a surface groove in a fashion similar to the type II ACP involved in actinorhodin biosynthesis. The interaction is more extensive and deeper than seen in the ACP of MLSA2, the only other 4 helix type I ACP seen to bind its substrate [10]. In the simulation of ACP3 with cognate substrate we see only one acyl to ACP surface binding event from five 50 ns simulations, suggesting that binding events are rare. However, when this simulation with acyl chain sequestration is extended to 1 microsecond duration that initial binding event lasts only ~30 ns, but a second alternative mode is seen for ~14 ns, subsequently followed by a return to the original binding groove for a duration of greater than half the simulation time.

One interpretation of the relative infrequency of binding in the five 50 ns second simulations compared to the length of binding seen in the 1 microsecond simulation is that binding depends on the conformation and/or dynamics of the ACP and that the 1 microsecond simulation had the ACP in the correct configuration to undergo binding. This hypothesis is tempting since it provides one mechanism by which interaction between ACP and environment might change the substrate binding affinity of the ACP.

The structural elements that make up the binding groove seen in the WT ACP3 simulations show the greatest backbone fluctuations during the simulation. Of these, helix III and both terminii of helix II show the greatest variation in the magnitude of fluctuations between different time periods (Fig F in S1 Supporting Information File). Repositioning helix III in response to ligand binding is common to many ACP systems, the length and degree of reduction of the acyl substrate that is bound to the phosphopantetheine being able to change the position of helix III with minimal perturbation to the rest of the ACP [6]. This hints at a mechanism by which the ACP can flag the substrate that is attached and thus ensure interaction with the correct partner protein [6]. Helix III, along with helix II, is also implicated in the binding and mechanism of transfer of substrate from AcpP to FabA, with the phosphopantetheine arm entering the FabA structure and the cavity in AcpP closing such that it resembles the conformation found in apo-AcpP [30]. Crystallography, NMR and molecular dynamics simulation together evidence a mechanism of FabA:AcpP interaction via helices II and III of the ACP of *E*. *coli*. Upon binding, helix III moves allowing the substrate sequestered in the core of the ACP to slide into FabA with a concerted or subsequent collapse of the hydrophobic pocket that had sequestered the acyl chain. A similar interaction is hinted at in the binding of AcpP to LpxD [31]. Indeed, helices II and III and the C-terminus of loop I are the regions most frequently interacting with other proteins [32], i.e. the very same regions of the ACP that are seen here to mediate substrate-ACP interactions, and show high degrees of backbone fluctuation.

## Conclusions

Simulations of ACP2 and ACP3, examples of *trans*-AT ACPs, show that when substrate sequestration occurs it occurs in a surface groove. Binding is mediated by the most dynamic parts of the ACP structure. In contrast, AcpP, part of a type II FAS, sequesters the substrate into its core. Three point mutations changed the way ACP3 could interact with a fully saturated carbon chain, allowing it to bury the carbon chain in its core in a somewhat similar fashion to an FAS, although binding to the surface groove, as in WT ACP3 simulations, was also seen, and may be a precursor to being bound into the core of this ACP3 mutant. Similarly, mutating AcpP to be more ACP3-like abrogated its ability to bury a fatty acid substrate, which instead, when sequestered to the ACP fold, bound to an ACP3 like surface groove.

Previously we found that ACPs mediating beta-branched modifications had distinct sequences [16]. It seems evident that some ACPs have sequence and structure that is adapted to their context in terms of the enzymic domains they interact with and/or the substrates that they carry. I.e. it is not enough to simply be an ACP, the ACP needs to be a specific type of ACP, something that has been seen in peptide carrier proteins [33] and pointed to by recent phylogenetic analysis [32] but which generally seems to have been little studied. The ability to form a substrate cavity is seen in the simulations here as one mode by which ACPs can be distinguished from one another, although the functional importance of this difference is unclear. The results also suggest that changing only a few amino acids may be sufficient to change an ACP from one type to another.

## Supporting information

S1 Supporting Information File Additional details of methods and results, including all supplementary figures and the additional parameters developed for these simulations. (DOCX)
