# Supplementary material for: Structure, function and dynamics in acyl carrier proteins
Source: PLoS One. 2019 Jul 10;14(7):e0219435. doi: 10.1371/journal.pone.0219435 (PMC6619796; doi:10.1371/journal.pone.0219435)
Supplement: S1 Supporting Information File — (DOCX) [file pone.0219435.s001.docx]

**Supporting Information**

Structure, Function and Dynamics in Acyl Carrier Proteins.

Rohit Farmer^1,2^, Christopher Morton Thomas^1,3^, Peter James Winn^1,3,4*^

^1^School of Biosciences, University of Birmingham, Edgbaston, Birmingham, B15 2TT, UK

^2^Department of Computational Biology and Bioinformatics, Jacob Institute of Biotechnology and Bioengineering, Sam Higginbottom University of Agriculture, Technology and Sciences, Allahabad, 211007, India. ^3^The institute of Microbiology and Infection, University of Birmingham, Edgbaston, Birmingham, B15 2TT, UK. ^4^Centre for Computational Biology, University of Birmingham, Edgbaston, Birmingham, B15 2TT, UK

KEYWORDS Acyl Carrier Protein (ACP), Type I Polyketide Synthases (PKS), Mupirocin, Molecular Dynamics Simulation and Ligand Sequestering.

*Corresponding Author: p.j.winn@bham.ac.uk

**S1. Supplementary Methods**

**Molecular dynamics simulation parameter determination**

Since force fields in GROMACS are optimized for protein and nucleic acid simulation, parameters are given only for the standard amino acids, nucleotides and a few counter ions. However, GROMACS allows the addition of extra parameters to represent systems which consist of atoms not represented by the standard set of parameters. While working with the AMBER force fields small molecules can be simulated using parameters from the general amber force field (GAFF). GAFF was created to be compatible with the AMBER force fields (1) and is part of the AMBER molecular dynamics package which is not included in GROMACS by default.

To carry out molecular dynamics simulation with the phosphopantetheine and other molecules attached to the serine on ACP a new residue type was created for each serine ligated with a ligan d. Since, these new residues were not a part of the standard amino acid set GROMACS did not have the parameters to carry out the simulation. For this reason parameters from the GAFF were added to the AMBER 99SB-ILDN (2) force field. The GAFF parameters were obtained from the AMBER molecular dynamics package and the values which differed in units from GROMACS format were converted using several in house Perl scripts.

To derive partial atomic charges for the new residues, AMBER force field compatible RESP charges were calculated using the RED server (http://q4md-forcefieldtools.org/REDS/ (3–5). For the calculation of RESP charges the RED server requires the structure file in a P2N format. The program Ante RED, available in the RED server, converts a PDB file into the P2N format. The RED IV server was used to calculate RESP-A1A charges for all the ligands, using the Gaussian 2009 D.01 quantum mechanics program. Fully automated mode 1 was chosen while running RED IV which performs geometry optimisation as well as charge fitting. Once the charges were calculated a new entry was created for each new residue type in the *aminoacids.rtp* database of GROMACS. The new residue name was also updated in the *residuetype.dat* database. For each new residue the corresponding hydrogen atoms were also created in the *aminoacids.hdb* database. While determining charges for the new moiety attached to an existing amino acid, the charges for the serine side chain were varied but the backbone atoms of the parent residue were kept the same as that of the original force field. **Supplementary Section S2.7** lists the modified forcefield parameters that were added to AMBER99SB-ILDN.

**Change in cavity volume during the course of simulations**

To detect the formation and change in the volume of the proposed cavity in the ACP structures during the course of molecular dynamics simulations a third party GROMACS plugin *trj_cavity* was used (6). It takes a GROMACS topology and trajectory as the input and an optional seed value in the form of Cartesian coordinates to initiate the cavity detection at a particular position on the protein. Here, the seed value was calculated using the PASS program from the MetaPocket Server (7). The default value of 5 was set for dimensions with a grid size of 1.3. The default grid size is 1.4 Å that represents the size of a water molecule. The grid size of 1.3 was decided after the observation that, for ACP3, with a grid size of 1.4 there was no cavity detected in many of the frames where a human would deem there to be one. As the proposed cavity is highly surface exposed the void spaces were not deep enough to find protein in all the five directions. The grid size of 1.3 did not eliminate the possibility of no cavity detection but upon visual inspection of the trajectories there were fewer frames found with no cavity mapped. Upon decreasing the dimension size to 4 and keeping the grid size to default 1.4 more frames were detected with cavities but there were more instances of spill over. Here, spill over means that the probe couldn’t find a protein atom in 5 directions and hence ran off the intended cavity space. This could be controlled with a cut-off distance, however various cut-off distances were tried and there wasn’t much improvement. Decreasing the grid size lower than 1.3 led to the probe going through the protein interior thus mapping the whole or the majority of the structure as a potential cavity. A grid size lower than 1.3 with an increased atomic radii was also tried but again there wasn’t much improvement and at the same time the system seemed to be more unrealistic. Therefore, after comparing different parameter sets the final set of parameters were decided to be dimension of 5, 1.3 grid spacing, 9 cutoff distance and atomic radii from the AMBER99SB-ILDN force field.

**Calculation of Protein Structural Properties During the Simulation.** Backbone root mean square deviations (RMSD) and root mean square fluctuations (RMSF) were calculated at every 10 ps of the simulation using GROMACS modules *g_rms* and *g_rmsf*. Hydrogen bonds and solvent accessible surface area (SASA) were calculated using GROMACS modules *g_hbond* and *g_sas*. Hydrogen bonds were calculated during the course of simulation at 10 ps intervals between the phosphopantetheine/acyl chains (excluding phosphopantetheine) and the protein/solvent separately. SASA were also calculated at 10 ps intervals for both the phosphopantetheine and the acyl chains (excluding phosphopantetheine). RMSDs were calculated between the FAS AcpP WT and ACP3 mutant structures at every 1 ns of the simulation trajectory using the Matt structural alignment program (8). Similarly, RMSDs were also calculated between the ACP3 WT and FAS AcpP mutant structures at every 1 ns of the simulation trajectory.

**ACP2 & ACP3 sequences**

>ACP2

VLLEHALQVLKRVLSPVVQWPEDRLDSDEPLERYGLDSMMVMTITAALQAQFGPLPTTLFFEYSTLRALAAYLCREHASTLVA

>ACP3

VADDECAQFLRQSLAAMLYCEPGQIRDGSRFLELGLDSVIAAQWIREINKHYQLKIPADGIYTYPVFKAFTQWVGTQL

**ACP2 homology modelling**

Homology modelling of ACP2 was carried out using Modeller. The above mentioned ACP2 sequence was searched against PDB database using BLASTP to find the template structure. The crystal structure of peptidyl carrier protein from *Acinetobacter baumannii* (PDB ID 4HKG) was selected as the best hit with 36% sequence identity. Along with the sequence from 4HKG, sequences from ACP1 (the ACP from the module in MmpA N-terminal to ACP2) and AcpI from the CurA module from *Lyngbya majuscula* were also used to create the master alignment using Muscle (<https://www.ebi.ac.uk/Tools/msa/muscle/>) **(Fig S1)**. For the modelling step the alignment between the ACP2 and 4HKG from the master alignment was input into Modeller, which used the very fast function.


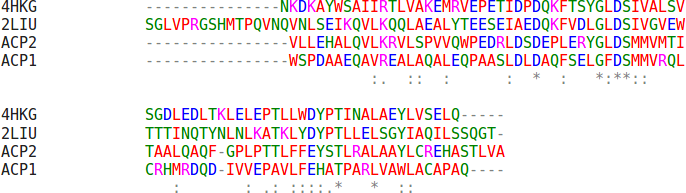


**Fig A:** Master sequence alignment used in ACP2 homology modelling.

**S2. Supplementary Results**

The compressed simulation co-ordinates, and structures used to create the figures in the main text can be downloaded from: <https://github.com/rohitfarmer/acp-dynamics>, along with scripts and parameters.

**S2.0 Modelling ACP2**

The overall quality (Z-Score) of the ACP2 model using the ProSA-web server (<https://prosa.services.came.sbg.ac.at/prosa.php>) was -6.75, which lies within the range of the Z-Scores of experimentally determined protein structures of similar size **(Fig S2 A)**. The plot of residue scores also showed overall negative values for energies as a function of amino acid sequence position **(Fig S2B)**. The modelled structure also passed the Verify 3D (<http://services.mbi.ucla.edi/Verify_3D/>) test with 85.90% of the residues having an average 3D-1D score >= 0.2. The Ramachandran plot calculated through PROCHECK (<http://servicesn.mbi.ucla.edu/SAVES/>) showed 68 out of 69 non-glycine and non-proline residues in the allowed regions with only one residue, Phe52, in the disallowed region, which is N-terminal to residues GPLP **(Fig S2C)**.


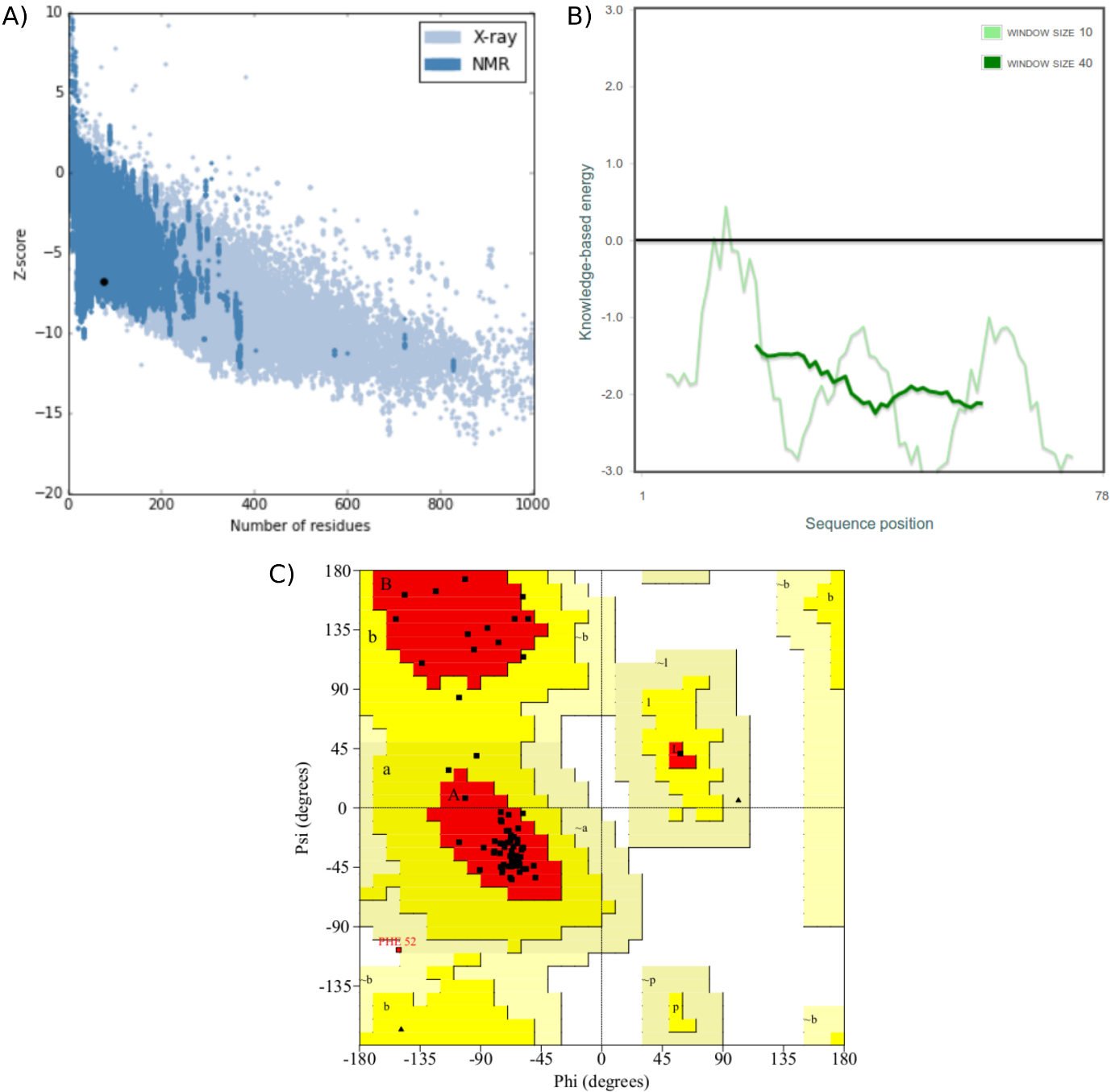


**Fig B**: **Quality check data for the structural model of ACP2**. A) The ProSA-web results’ Z-Score plot where the black dot is the score, -6.75, calculated for the model of ACP2. B) The ProSA plot of residue scores; negative values denote good structure quality. C) The Ramachandran plot produced by PROCHECK.

**S2.1 Tables**

**Table A:** Average values for cavity volume, hydrogen bonds and solvent accessible surface.

| **Label^!^** | **Simulation** | **Maximum cavity volume (Å^3^)*** | **Mean/ modal cavity volume (Å^3^) *** | **Mean number/ standard deviation of hydrogen bonds by PPT to: **** | | **Mean number & standard deviation of hydrogen bonds by acyl chain to: **** | | **Mean & SD of SASA (nm^2^) ***** |
| --- | --- | --- | --- | --- | --- | --- | --- | --- |
|  |  |  |  | **Protein** | **Solvent** | **Protein** | **Solvent** |  |
| 1. | Apo ACP3 WT (200ns) | 151.593 | 64.951/  52.728 | - | - | - | - | - |
| 2. | Apo ACP3 WT(1µs) | 151.593 | 62.856/  52.728 | - | - | - | - | - |
| 3. | Apo ACP3 W44L (200ns) | 173.563 | 75.408/  50.531 |  |  |  |  |  |
| 4. | Holo ACP3 WT (200ns) | 228.488 | 107.303/  109.850 | 1.180/ 1.266 | 11.612/ 2.313 | - | - | 5.876/ 0.309 |
| 5. | Holo ACP3 W44L (200ns) | 243.867 | 95.976/  92.274 | 2.376/ 1.577 | 10.473/ 1.945 | - | - | 5.275/ 0.297 |
| 6. | Acyl ACP3 WT (200ns) | 265.837 | 107.452/  103.259 | 0.415/ 0.769 | 10.341/ 1.879 | 2.618/ 1.478 | 5.719/ 1.968 | 4.943/  0.168 |
| 7. | Acyl ACP3 WT (1µs) | 265.837 | 82.463/  72.501 | 0.376/ 0.654 | 8.266/ 5.039 | 2.221/ 1.039 | 5.493/ 1.604 | 4.901/ 0.175 |
| 8. | Acyl ACP3 W44L (200ns) | 243.867 | 99.163/  57.122 | 0.445/ 0.778 | 8.193/ 4.815 | 1.295/ 1.007 | 6.825/ 1.767 | 4.920/ 0.197 |
| 9. | 14C  ACP3 WT (200ns) | 195.533 | 94.186/  96.668 | 0.463/ 0.771 | 10.649/ 1.967 | - | - | 4.288/ 0.151 |
| 10. | Acyl ACP2 WT (200ns) | 248.261 | 101.973/  92.274 | 1.430/ 0.772 | 7.504/ 1.658 | 1.060/ 0.535 | 5.748/ 1.466 | 4.433/ 0.127 |
| 11. | Acyl ACP3 L36A-I61A (3 X 200ns) | 206.518 | 71.246/  50.531 | 0.599/  0.814 | 9.017/  4.83 | 0.739/  0.991 | 6.581/  3.745 | 4.844/  0.211 |
|  |  | 186.745 | 70.143/  50.531 | 0.664/  0.938 | 9.030/  5.527 | 0.875/  0.760 | 6.885/  3.994 | 4.868/  0.188 |
|  |  | 197.73 | 76.662/  63.713 | 0.531/  0.676 | 9.147/  4.797 | 1.053/  1.243 | 6.751/  3.818 | 4.907/  0.191 |
| 12. | Acyl ACP3 L36A-W44L-I61A (200ns) | 340.535 | 94.37/  54.925 | 0.973/  0.918 | 10.019/  1.777 | 1.666/  1.205 | 5.506/  2.04 | 4.964/  0.160 |
| 13. | 14C ACP3 L36A-I61A (200ns) | 263.640 | 79.325/  59.319 | 0.481/  0.916 | 9.299/  5.632 | - | - | 4.303/  0.145 |
| 14. | 14C ACP3 L36A-W44L-I61A (200ns) | 342.732 | 118.197/  105.456 | 0.390/  0.762 | 10.866/  1.793 | - | - | 4.311/  0.150 |
|  |  | 246.064 | 113.206/  112.047 | 0.679/  0.354 | 11.163/  1.738 | - | - | 4.329/  2.167 |
| 15. | 14C AcpP WT (200ns) | 417.43 | 163.258/  123.032 | 0.689/  0.905 | 9.262/  5.259 | - | - | 4.261/  0.175 |
| 16. | 14C AcpP A34L-A59I (200ns) | 382.278 | 125.728/  98.865 | 0.494/  0.771 | 8.917/  5.015 | - | - | 4.314/  0.144 |
| 17. | 14C AcpP A34L-L42W-A59I (200ns) | 305 | 133.183/  129.623 | 0.278/  0.663 | 11.703/  1.871 | - | - | 4.285/  0.152 |

*Maximum, mean and model cavity volume was calculated with the ligand removed from the co-ordinates.

**Hydrogen bonds were calculated separately for the phosphopantetheine and acyl moieties.

***SASA was calculated separately for the phosphopantetheine and acyl moieties. SASA for the acyl ACPs only shows the value for the acyl moieties excluding the phosphopantetheine.

^!^Cross references with Table 1..

**Table B:** Pearson’s Correlation between cavity volume and backbone RMSD / hydrogen bonds; demonstrating the more similar ACP3 is to AcpP the larger the cavity it has.

| **Simulation** | **Correlation (R) between cavity**  **volume and BACKBONE RMSD from the**  **AcpP** | | | | **Correlation between cavity volume and hydrogen bonds with the protein** | | | | **Correlation between the hydrogen bonds formed by the ligand with protein and solvent** | | | |
| --- | --- | --- | --- | --- | --- | --- | --- | --- | --- | --- | --- | --- |
|  | **Wild Type** | **W44L** | **L36A-I61A** | **L36A-W44L-I61A** | **Wild Type** | **W44L** | **L36A-I61A** | **L36A-W44L-I61A** | **Wild Type** | **W44L** | **L36A-I61A** | **L36A-W44L-I61A** |
| Apo ACP3 (200ns) | -0.241 | -0.309 | - | - | - | - |  |  | - | - |  |  |
| Apo ACP3 (1 s) | -0.241 | - | - | - | - | - |  |  | - | - |  |  |
| Holo ACP3 (200ns) | -0.225 | -0.601 | - | - | 0.041 | 0.021 | -0.115  0.395  0.143 | 0.251 | -0.433 | -0.222 | -0.101  0.04  -0.109 | -0.260 |
| Acyl ACP3 (200ns) | -0.111 | -0.435 | 0.007  -0.04  -0.08 | -0.235 | 0.164 | 0.015 | -0.022  -0.168  -0.118 | -0.020 | -0.653 | -0.323 | -0.160  -0.067  -0.086 | -0.490 |
| Acyl ACP3 (1µs) | -0.245 | - | - | - | 0.203 | - | - | - | -0.429 | - | - | - |
| 14C ACP3 (200ns) | -0.538 | - | 0.026 | -0.058  -0.176 | - | - | - | - | - | - | - | - |
| Acyl ACP2 (200ns) | 0.143 | - | - | - | 0.012 | - | - | - | -0.207 | - | - | - |
|  |  |  |  |  |  |  |  |  |  |  |  |  |

**S2.2 The Root Mean Square Deviation of the Backbone of ACPs from their initial conformation.**

**
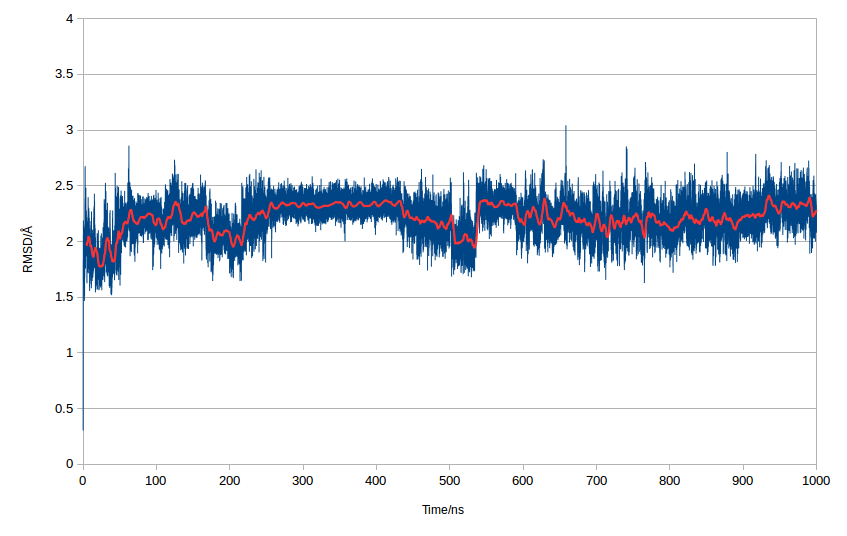
**

**Fig C**: **The RMSD of the backbone atoms of Apo ACP3 WT from the NMR structure over time (1 µs).** The red line represents the running average over 500 frames.

**
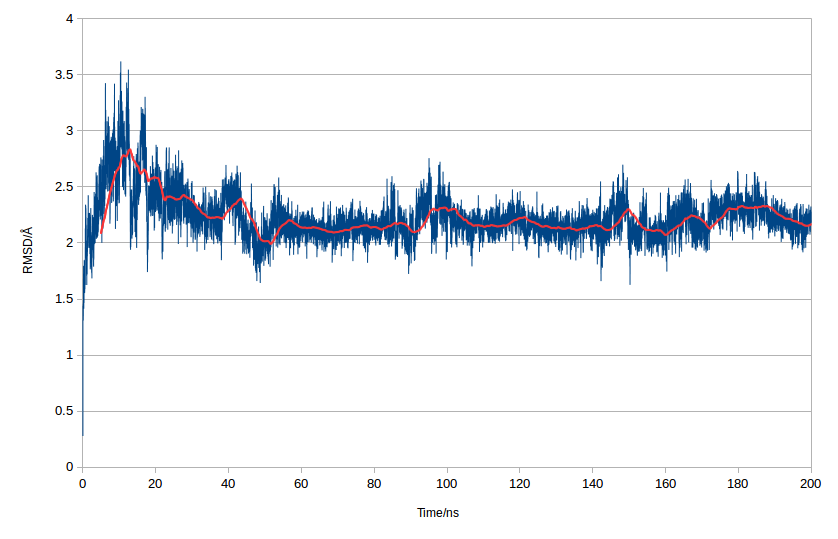
**

**Fig D**: **The RMSD of the backbone atoms of Holo ACP3 WT from the NMR structure over time (200 ns).** The red line represents the running average over 500 frames.

**
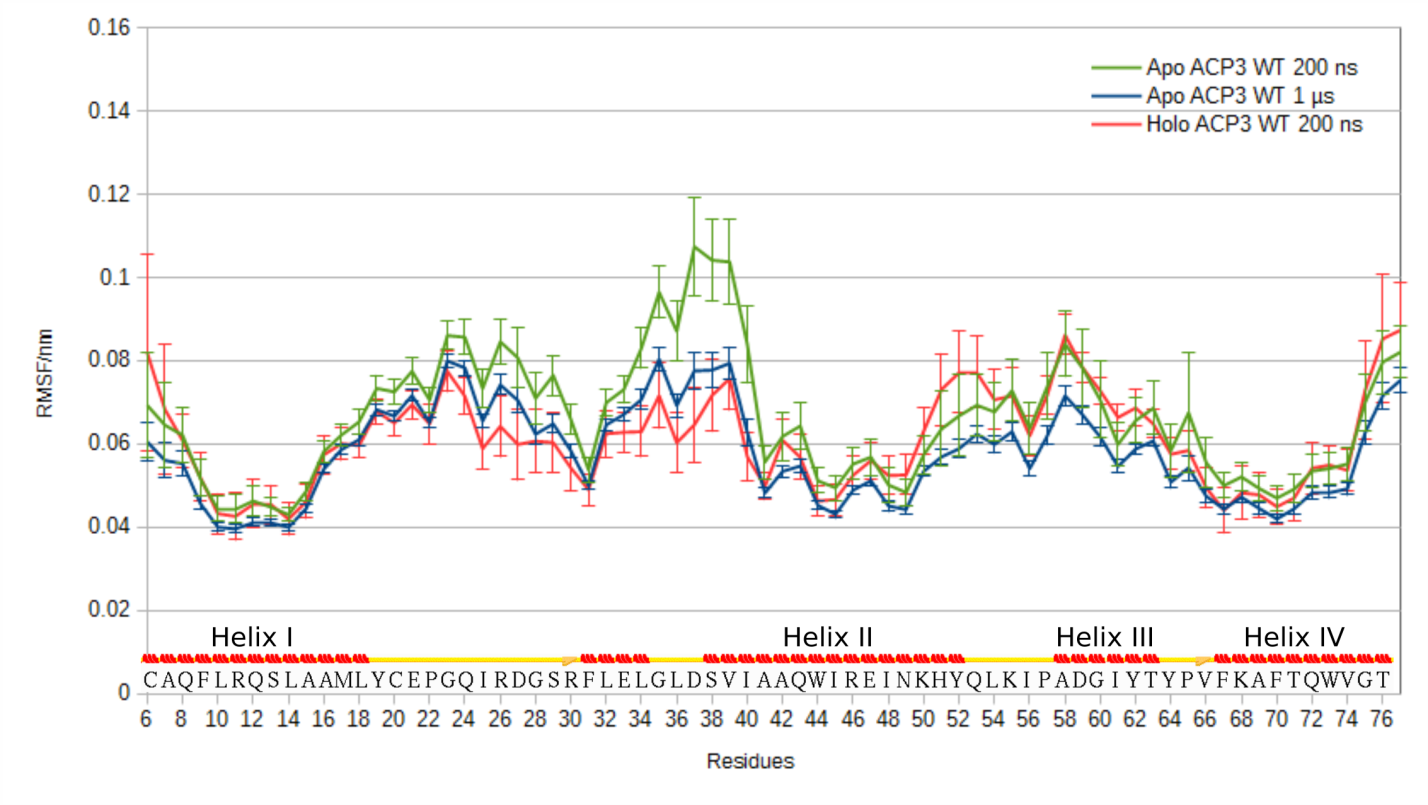
**

**Fig E:** **The backbone RMSF per residue of apo (1 µs & 200 ns) and holo (200 ns) forms of ACP3 WT.** The RMSF was calculated for 10 ns windows, with 1000 frames per 10 ns. For apo ACP3 WT, the blue line corresponds to the average of 100 10 ns RMSFs and for holo ACP3 WT, red and green lines corresponds to the average of 20 10 ns RMSFs, with the error bars representing confidence intervals at 95% confidence level. Values for first five and last two residues were not included in the graph for clarity. Sequence and corresponding secondary structures are marked on top of the x-axis.

| 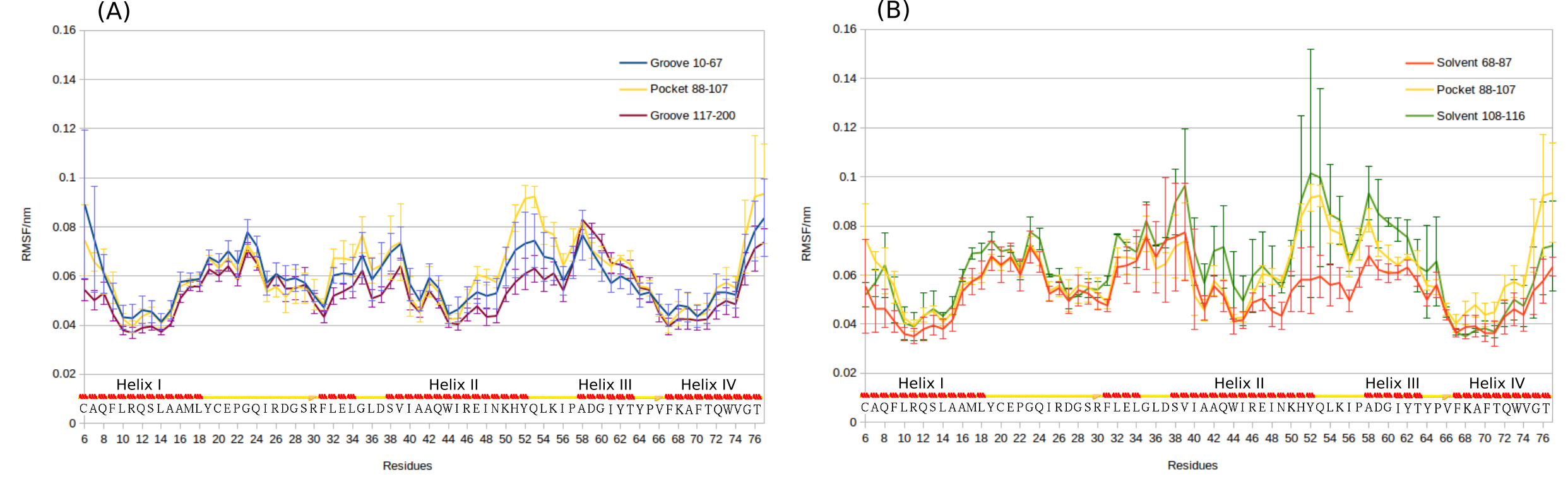 |
| --- |
| (a) |
| 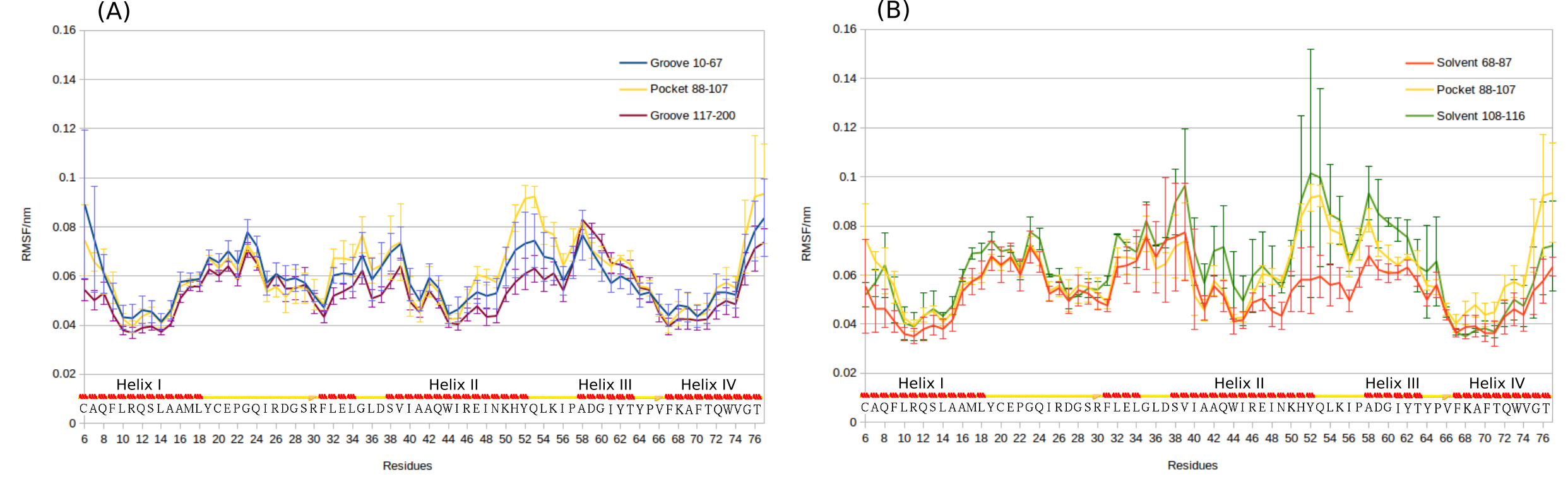 |
| (b) |

**Fig F:** **The backbone RMSF per residue of holo ACP3 WT over 200 ns simulation**. The RMSF was calculated for 5ns windows, with 500 frames per 5ns. Each line corresponds to the average of multiple 5 ns RMSF with the error bars representing confidence intervals at 95% confidence level estimated from the standard deviation over each multiple windows analysed. a) Comparison between the pocket and two groove binding modes. b) Comparison between the pocket binding and the solvent exposed modes. The line for the pocket data provides a reference point between A and B. Values for first five and last two residues show extremely high RMSFs and were not included in the graph for clarity.

**S2.3 The formation and change in cavity volume during simulation of ACP2/3**

| **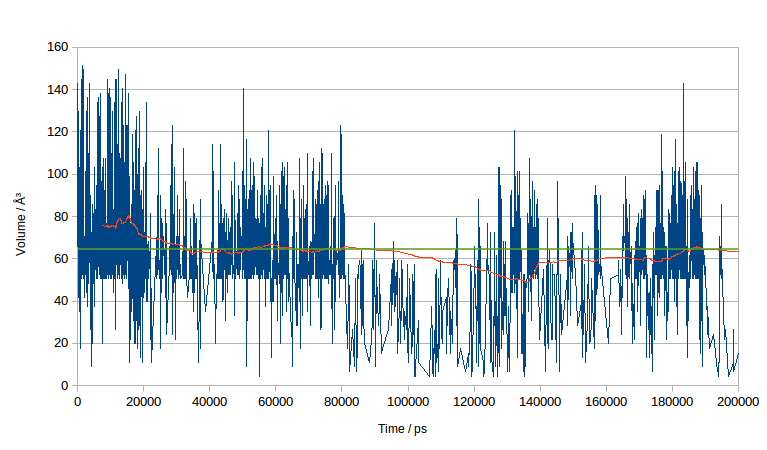**  (a) | 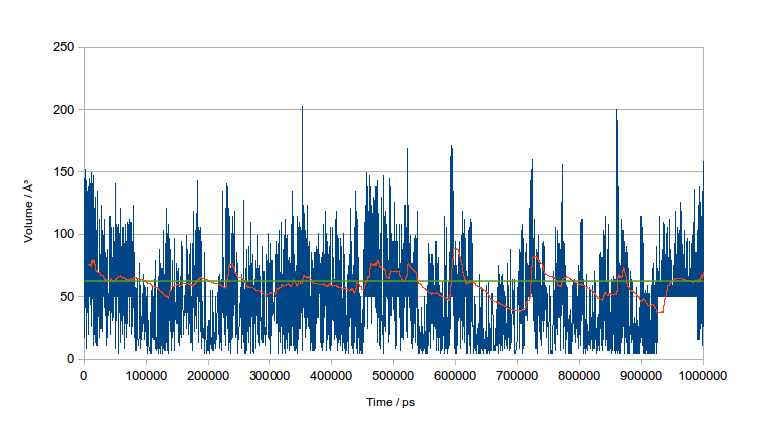  (b) |
| --- | --- |
| 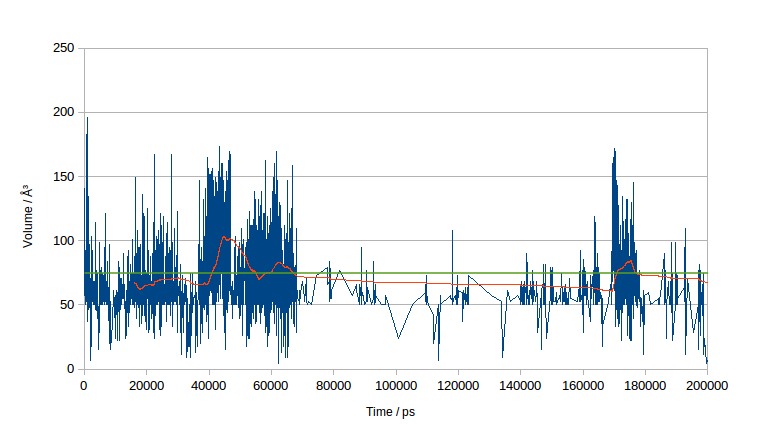  (c) | |

**Fig G:** **The formation and change in cavity volume during simulations of ACP3.** (a) apo ACP3 WT (200 ns); (b) apo ACP3 WT (1 µs); (c) apo ACP3 W44L (200 ns). The time frames which had a zero value for the volume were omitted from the plot. The red line represents the running average over 500 frames and the green line represents the mean.

| 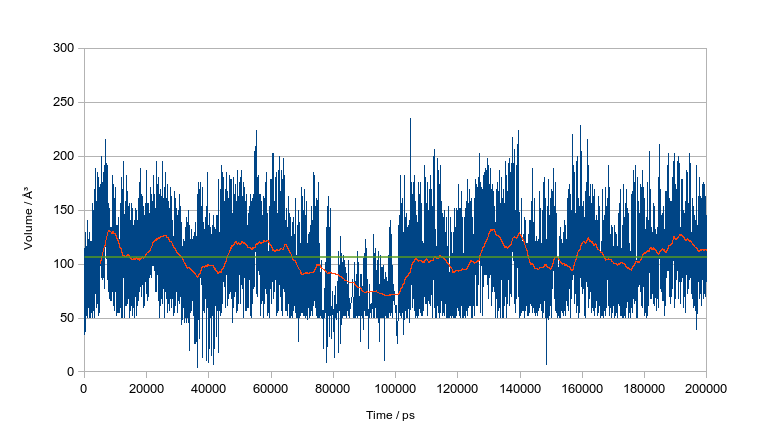  (a) | 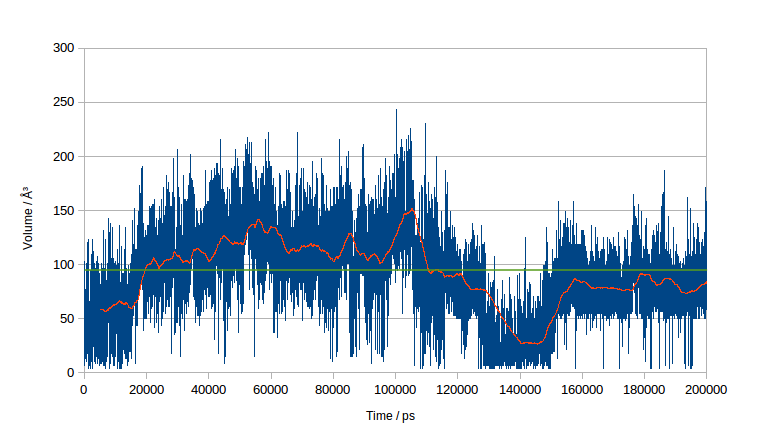  (b) |
| --- | --- |

**Fig H:** **The formation and change in cavity volume during simulations of ACP3.** (a) holo ACP3 WT (200 ns); (b) holo ACP3 W44L (200 ns). The time frames which had a zero value for the volume were omitted from the plot. The red line represents the running average over 500 frames and the green line represents the mean.

| 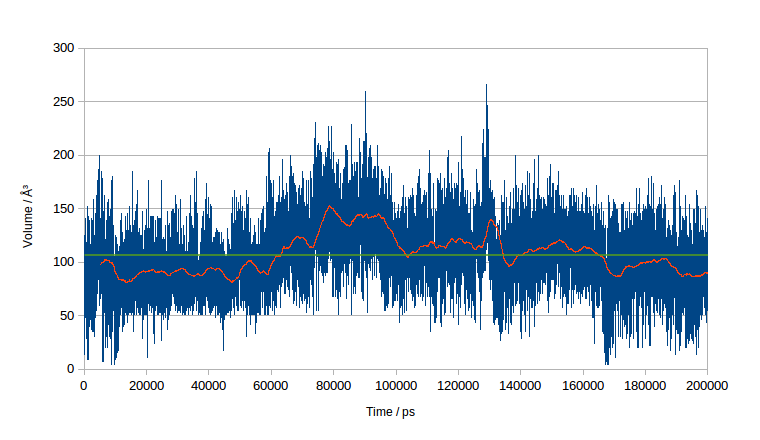  (a) | 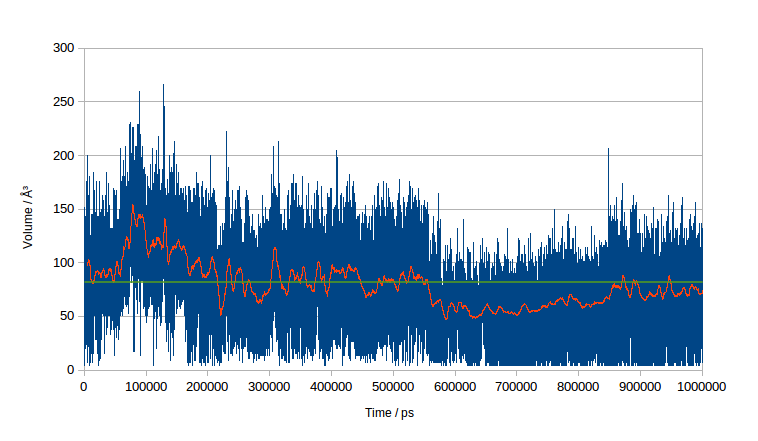  (b) |
| --- | --- |
| 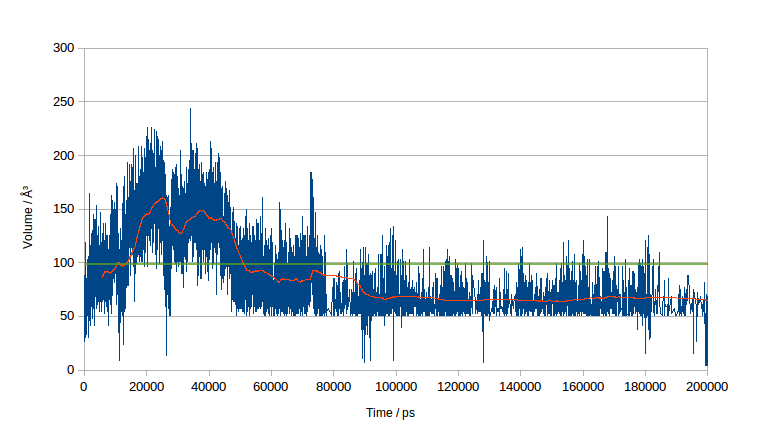  (c) | 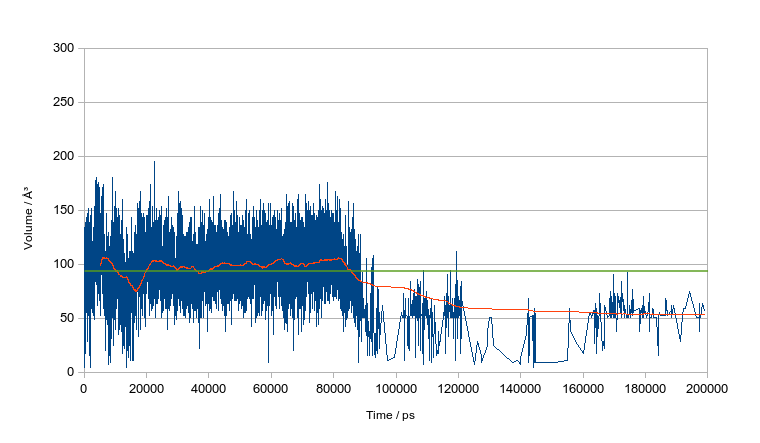  (d) |

**Fig I:** **The formation and change in cavity volume during simulations of ACP3.** (a) acyl ACP3 WT (200 ns); (b) acyl ACP3 WT (1 µs); (c) acyl ACP3 W44L (200 ns); (d) 14C ACP3 (200 ns). The time frames which had a zero value for the volume were omitted from the plot. The red line represents the running average over 500 frames and the green line represents the mean.


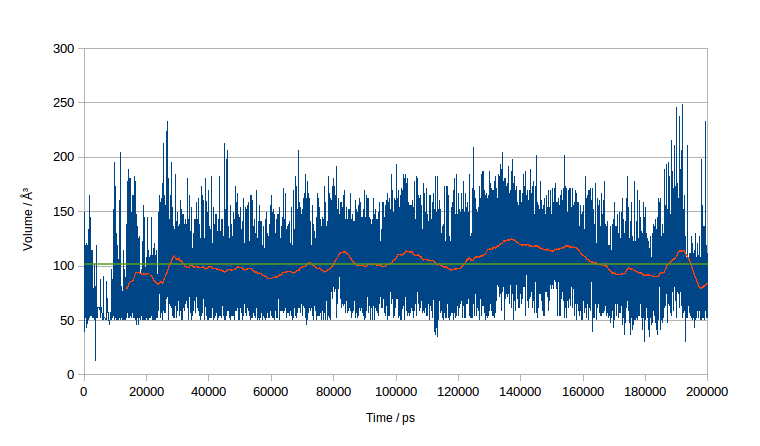


**Fig J:** **The formation and change in cavity volume during the simulation of acyl ACP2.** The time frames which had a zero value for the volume were omitted from the plot. The red line represents the running average over 500 frames and the green line represents the mean.

| 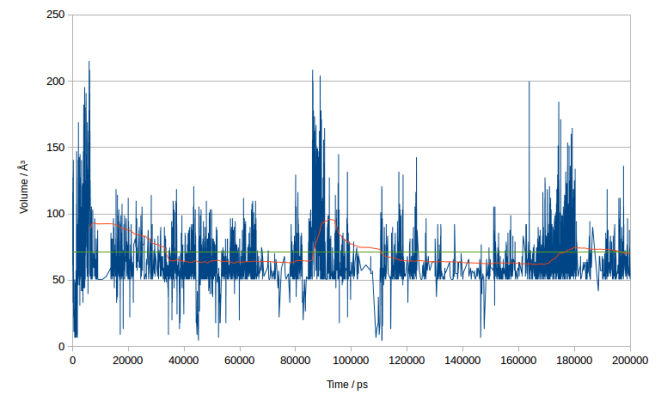  (a) | 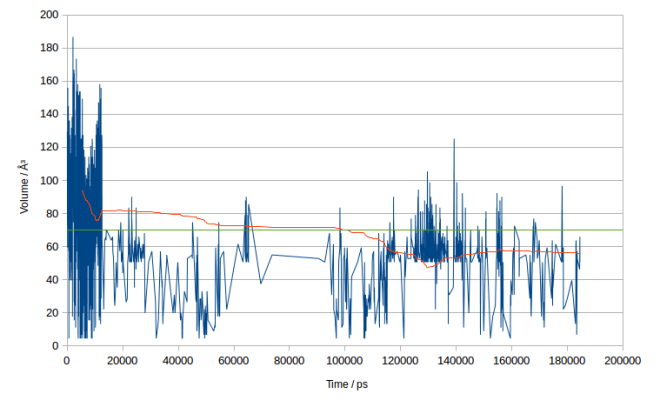  (b) |
| --- | --- |
| 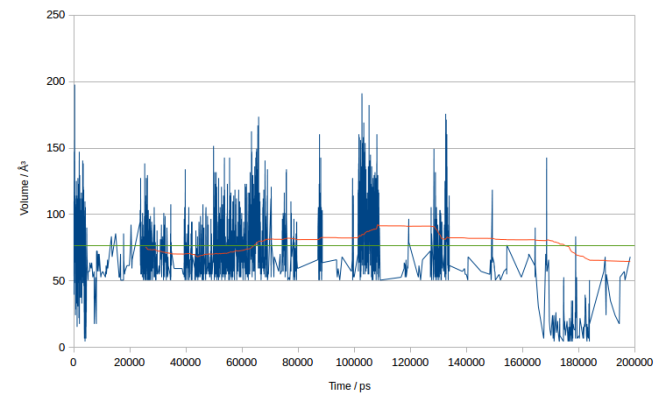  (c) | 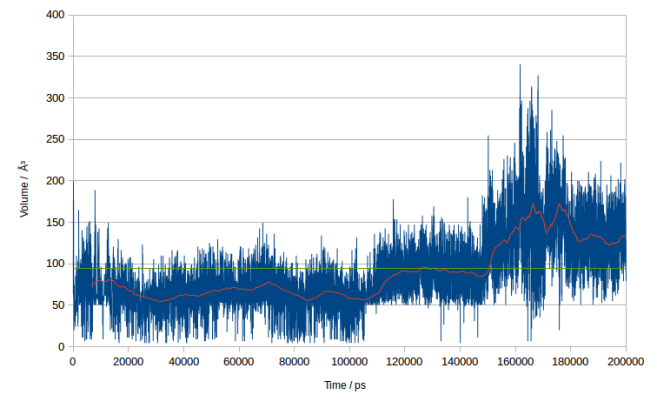  (d) |

**Fig K:** **The formation and change in cavity volume during the simulation of ACP3 mutants with cognate substrate bound.** (a) acyl ACP3 L26A-I61A (200 ns, replicate 1); (b) acyl ACP3 L26A-I61A (200 ns, replicate 2); (c) acyl ACP3 L26A-I61A (200 ns, replicate 3); (d) acyl ACP3 L26A-W44L-I61A (200 ns). The time frames which had a zero value for the volume were omitted from the plot. The red line represents the running average over 500 frames and the green line represents the mean.

| 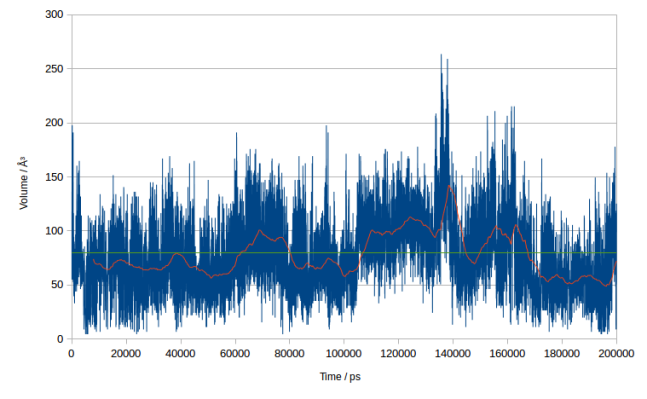  (a) | 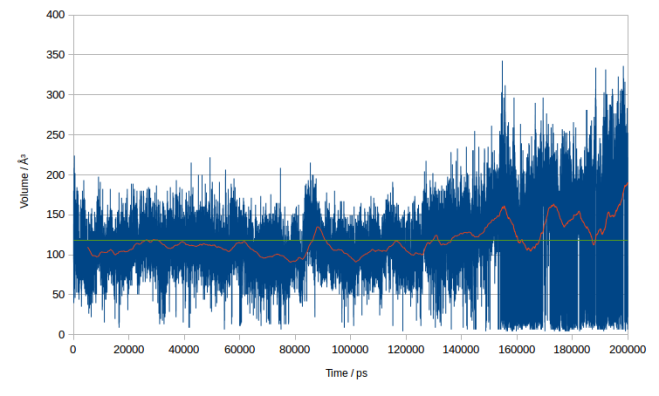  (b) |
| --- | --- |
| 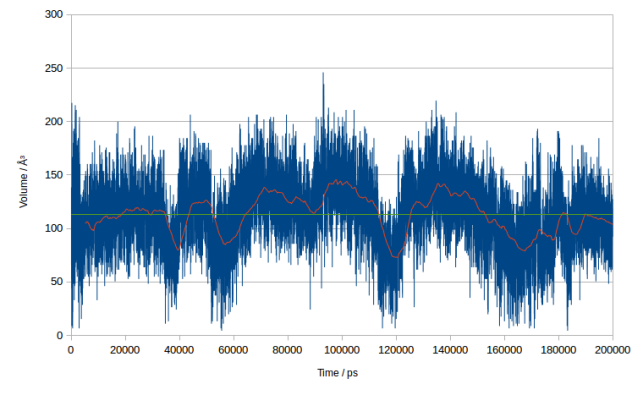  (c) | |

**Fig L:** **The formation and change in cavity volume during the simulation of ACP3 mutants with 14C substrate bound.** (a) 14C ACP3 L36A-I61A (200 ns); (b) 14C ACP3 L36A-W44L-I61A (200 ns, replicate 1); (c) 14C ACP3 L36A-W44L-I61A (200 ns, replicate 2). The time frames which had a zero value for the volume were omitted from the plot. The red line represents the running average over 500 frames and the green line represents the mean.

**S2.4 The formation and change in cavity volume during simulation of FAS AcpP**

| 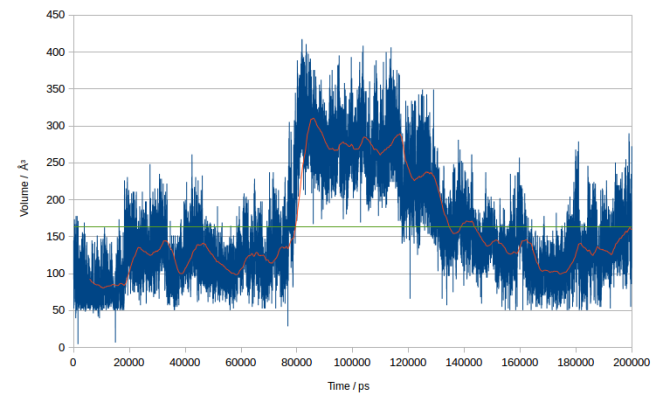  (a) | 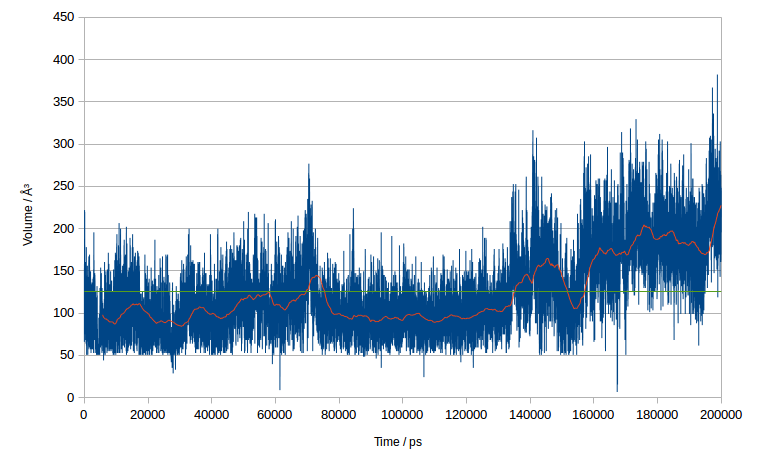  (b) |
| --- | --- |
| 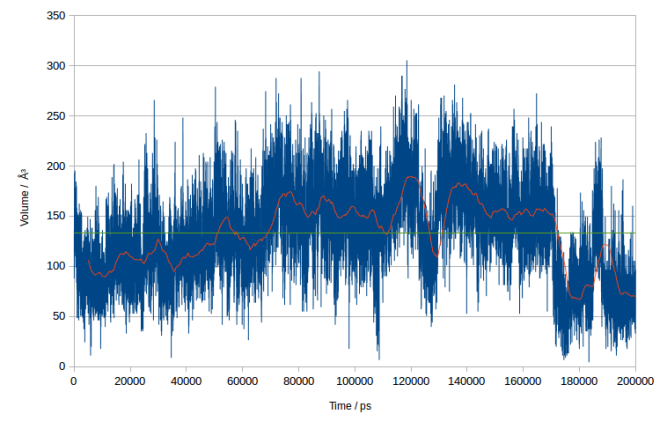  (c) | |

**Fig M:** The formation and change in cavity volume during simulations of AcpP. (a) 14C AcpP WT (200 ns); (b) 14C AcpP A34L-A59I (200 ns); (c) 14CAcpP A34L-L42W-A59I (200 ns). The time frames which had a zero value for the volume were omitted from the plot. The red line represents the running average over 500 frames and the green line represents the mean.

**S2.5 The backbone Root Mean Square Deviation of PKS ACPs from FAS AcpP over time.**

| 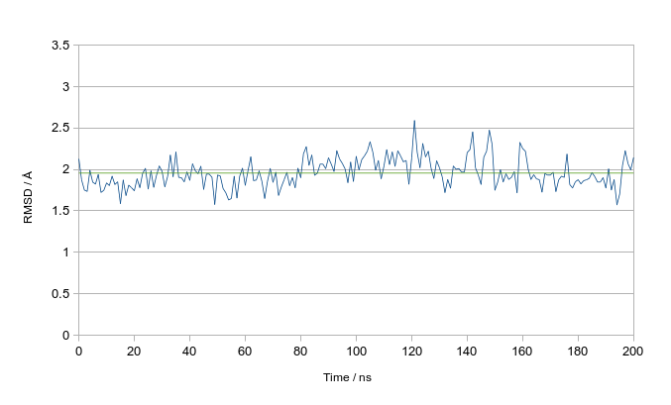  (a) | 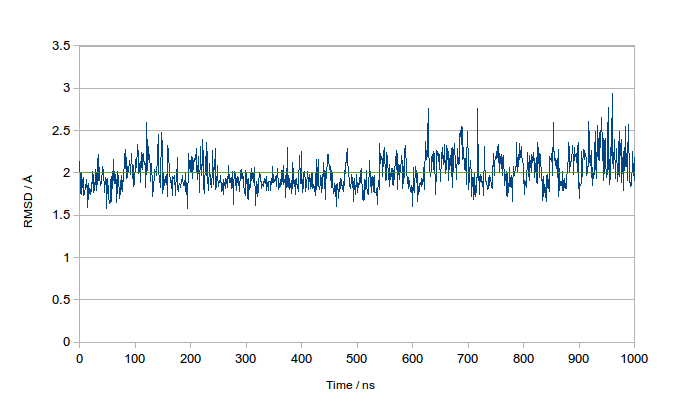  (b) |
| --- | --- |
| 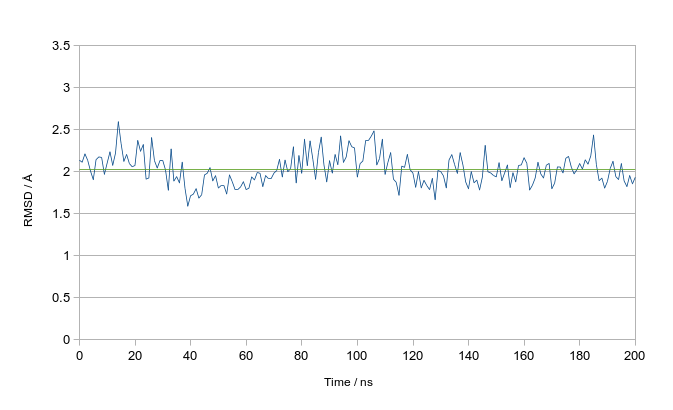  (c) | |

**Fig N:** **The backbone RMSD of simulated apo-ACP3 structures from the FAS crystal structure.** Comparisons between (a) FAS AcpP and apo ACP3 WT (200 ns); (b) FAS AcpP and apo ACP3 WT (1 µs); (c) FAS AcpP and apo ACP3 W44L (200 ns). The green line represents the mean.

| 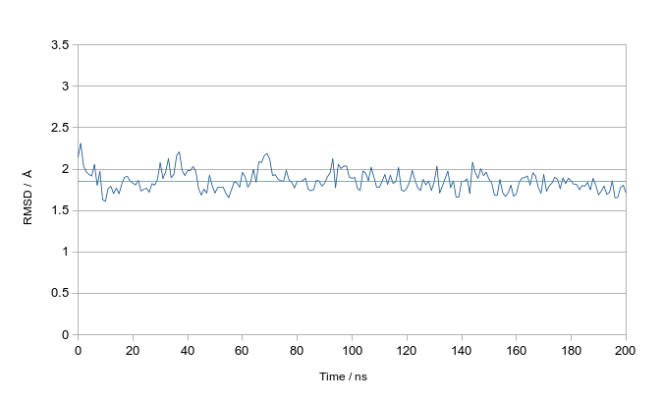  (a) | 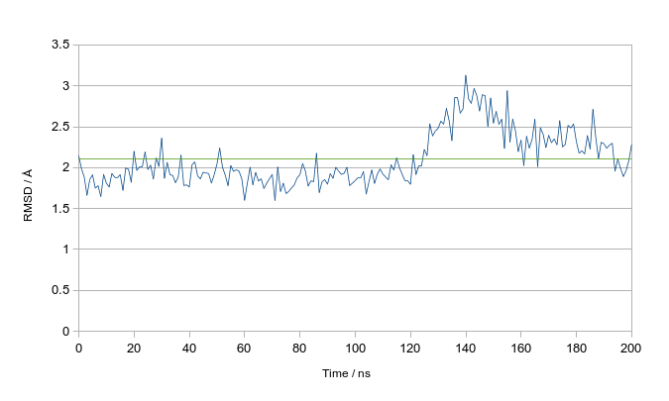  (b) |
| --- | --- |

**Fig O:** **The backbone RMSD of simulated holo-ACP3 structures from the FAS crystal structure.** Comparisons between (a) FAS AcpP and holo ACP3 WT (200 ns); (b) FAS AcpP and holo ACP3 W44L (200 ns). The green line represents the mean.

| 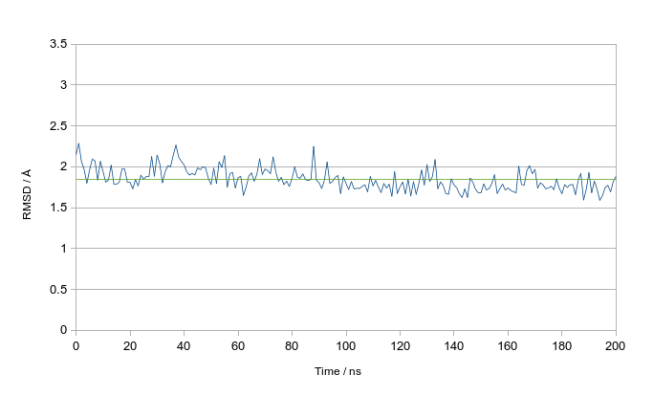  (a) | 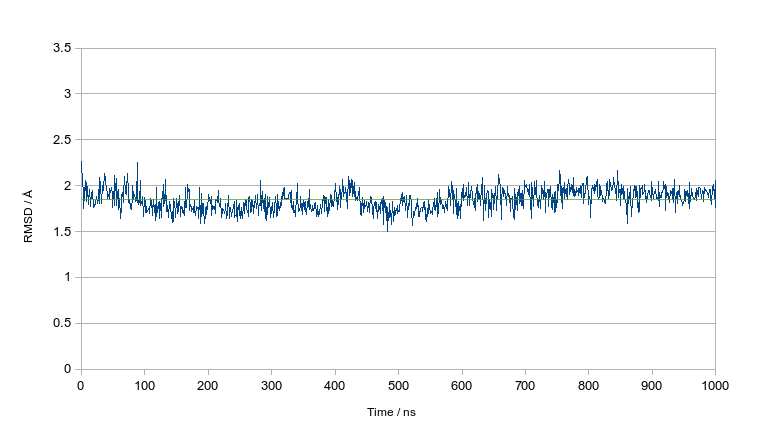  (b) |
| --- | --- |
| 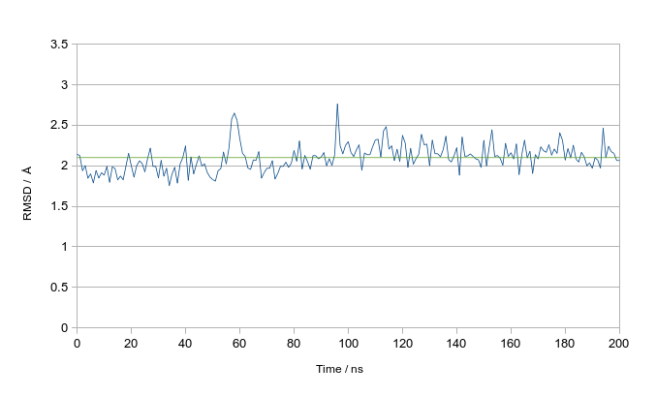  (c) | 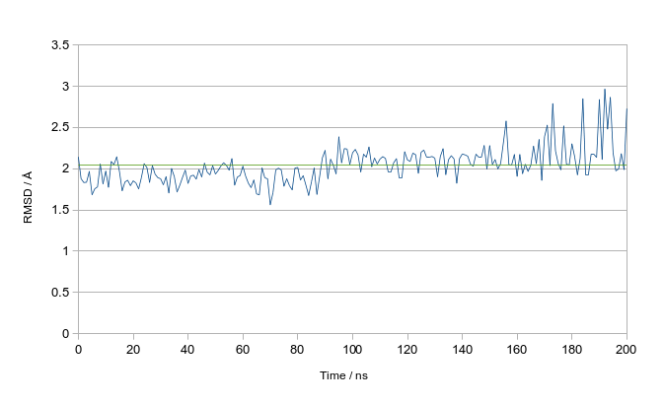  (d) |

**Fig P:** **The backbone RMSD of simulated acyl-ACP3 structures from the FAS crystal structure.** Comparisons between (a) FAS AcpP and acyl ACP3 WT (200 ns); (b) FAS AcpP and the acyl ACP3 WT (1 µs); (c) FAS ACP and acyl ACP3 W44L (200 ns); (d) FAS AcpP and acyl 14C ACP3 WT (200 ns). The green line represents the mean.


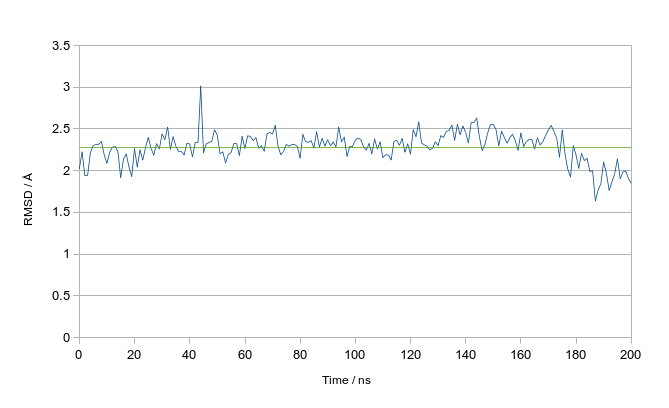


**Fig Q:** **The backbone RMSD between FAS AcpP and acyl ACP2 WT (200 ns).** The green line represents the mean.

| 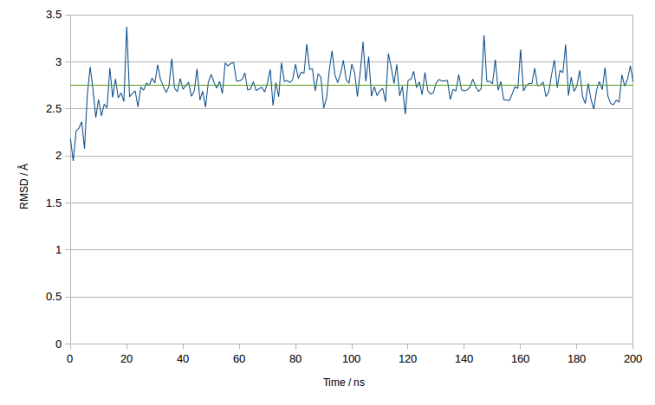  (a) | 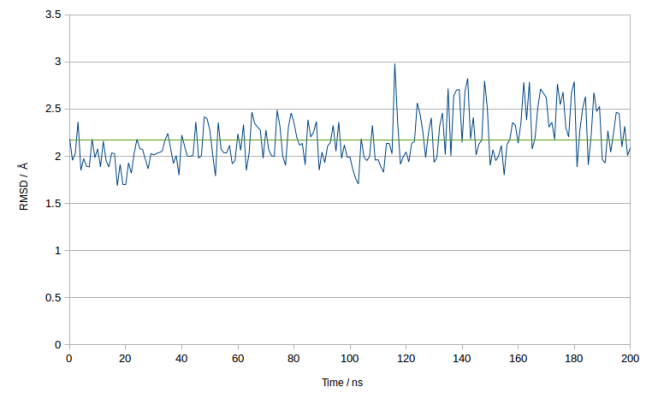  (b) |
| --- | --- |
| 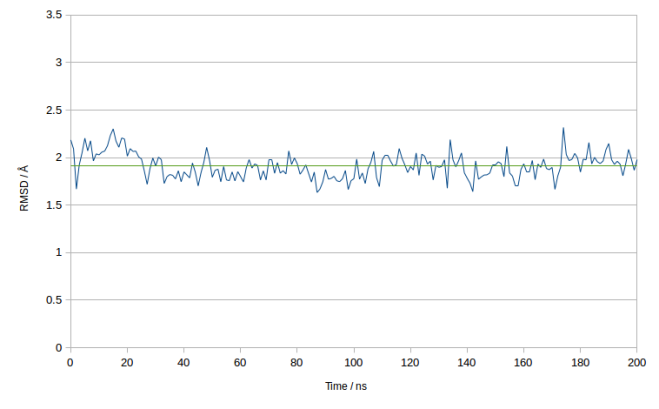  (c) | 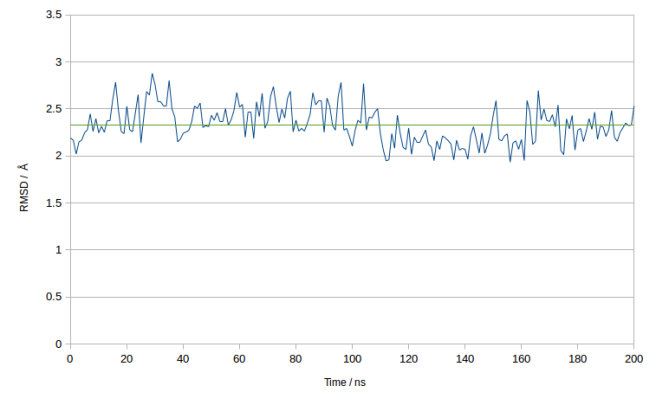  (d) |

**Fig R:** **The backbone RMSD of simulated mutant acyl-ACP3 structures from the FAS crystal structure.** Comparisons between (a) FAS AcpP and acyl ACP3 L36A & I61A (200 ns, replicate 1); (b) FAS AcpP and acyl ACP3 L36A-I61A (200 ns, replicate 2); (c) FAS AcpP and acyl ACP3 L36A-I61A (200 ns, replicate 3); (d) FAS AcpP and the acyl ACP3 L36A-W44L-I61A (200 ns). The green line represents the mean.

| 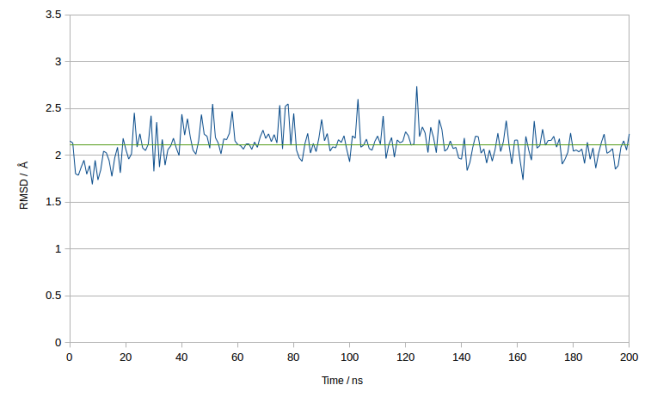  (a) | 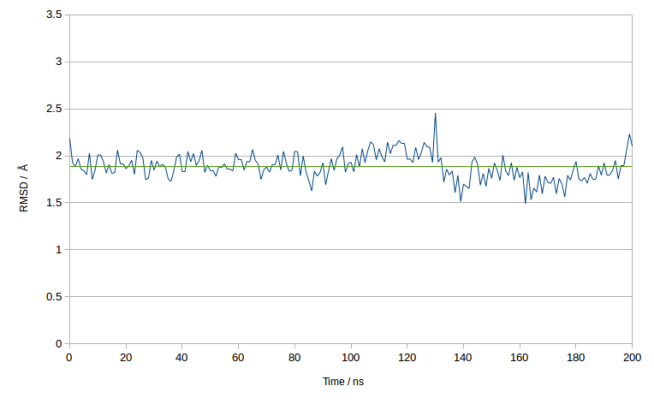  (b) |
| --- | --- |
| **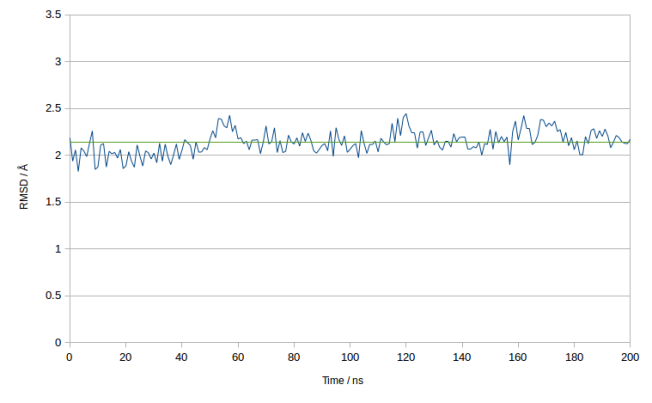**  (c) | |

**Fig S:** **The backbone RMSD of simulated 14C bound mutant ACP3 structures from the FAS crystal structure.** Comparisons between (a) FAS AcpP and 14C ACP3 L36A & I61A (200 ns); (b) FAS AcpP and 14C ACP3 L36A-W44L-I61A (200 ns, replicate 1); (c) FAS AcpP and 14C ACP3 L36A-W44L-I61A (200 ns, replicate 2). The green line represents the mean.

**S2.6 Simulation Snapshots and Structural Comparisons**

**
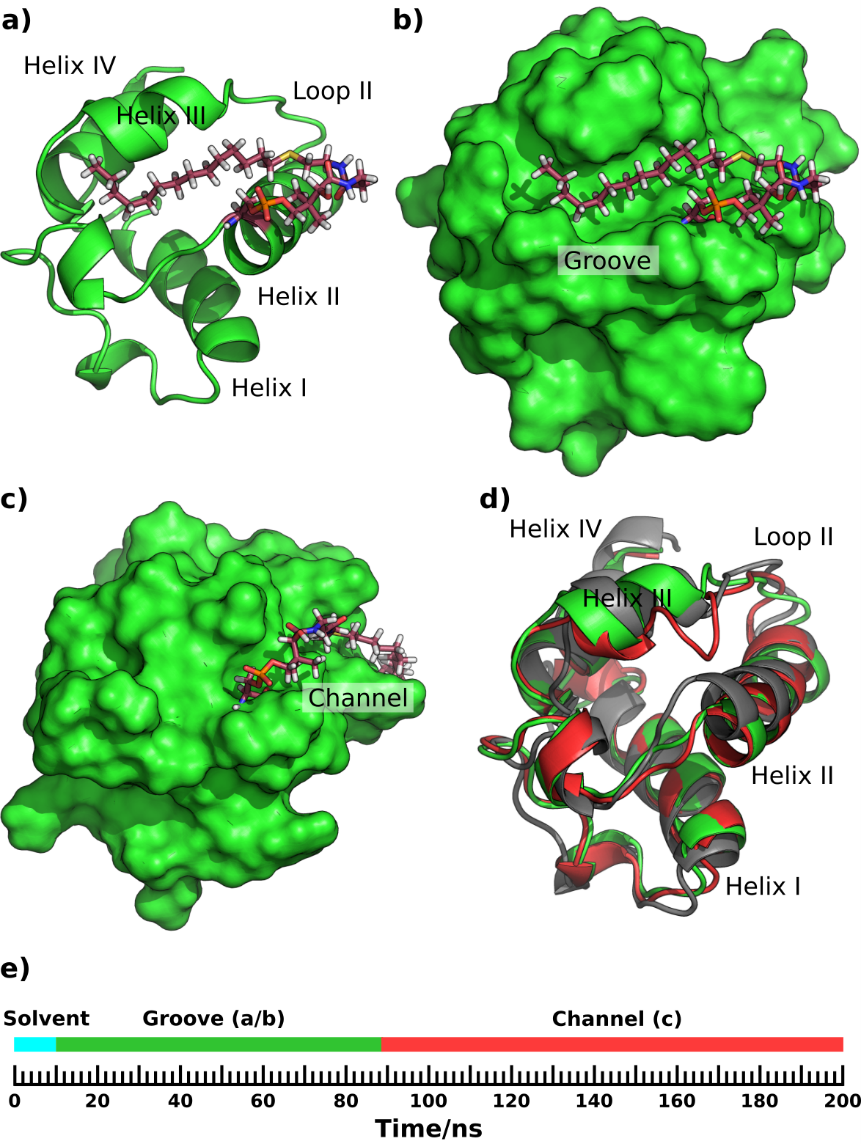
**

**Fig T:** **The 14C substrate binding to the surface groove in the 14C ACP3 WT 200 ns simulation.** a) & b) Cartoon & surface drawing respectively of the ACP with the 14C chain lying flat in the groove. c) The 14C chain at the surface in a narrow channel parallel to helix II and the loop II; loop connecting helix II and helix III. d) Superposition of the different conformations: starting structure in grey. e) Timeline of the simulation; colouring is the same as the ribbons in panel d. Orientation of the helices in all the figure panels is equivalent.

| 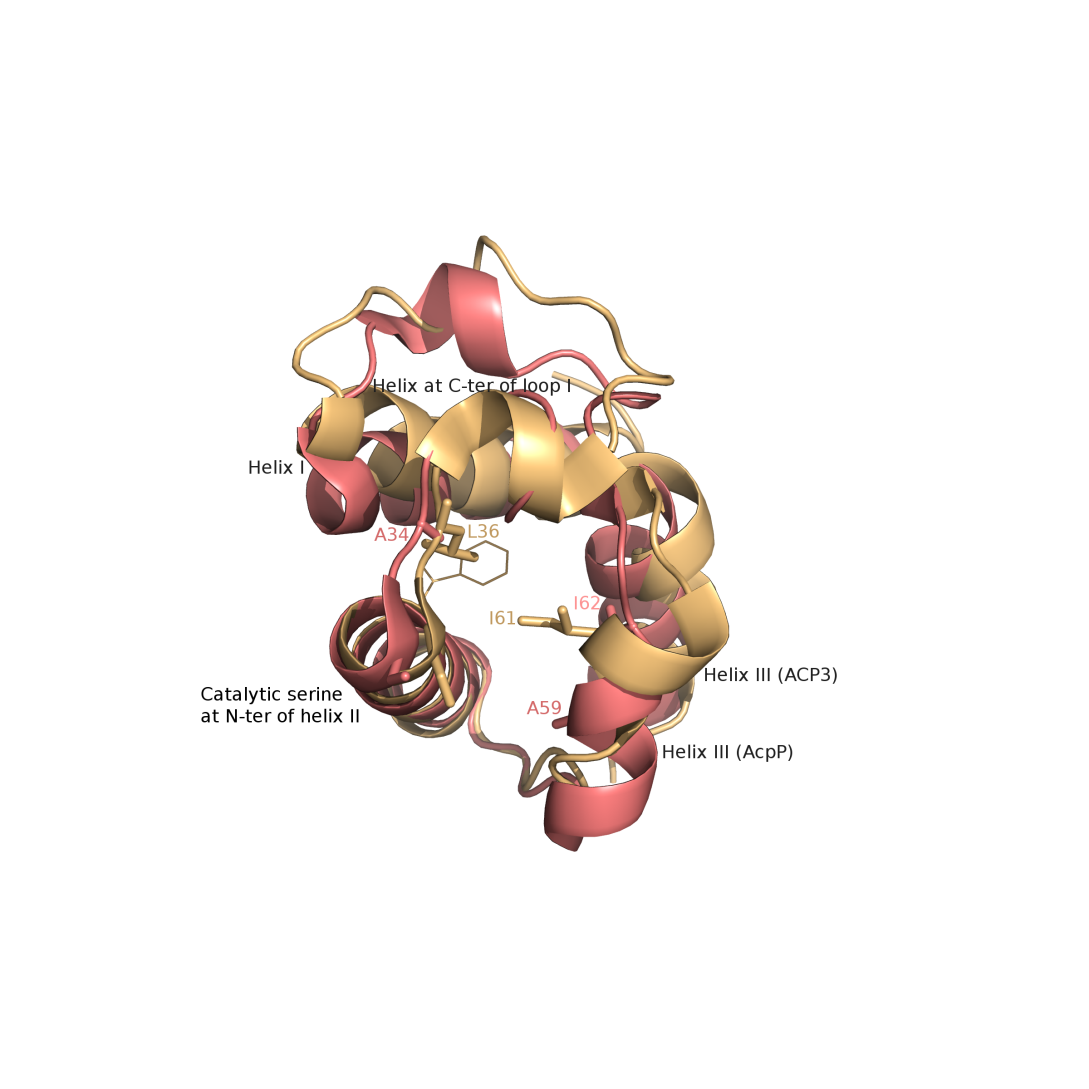 | 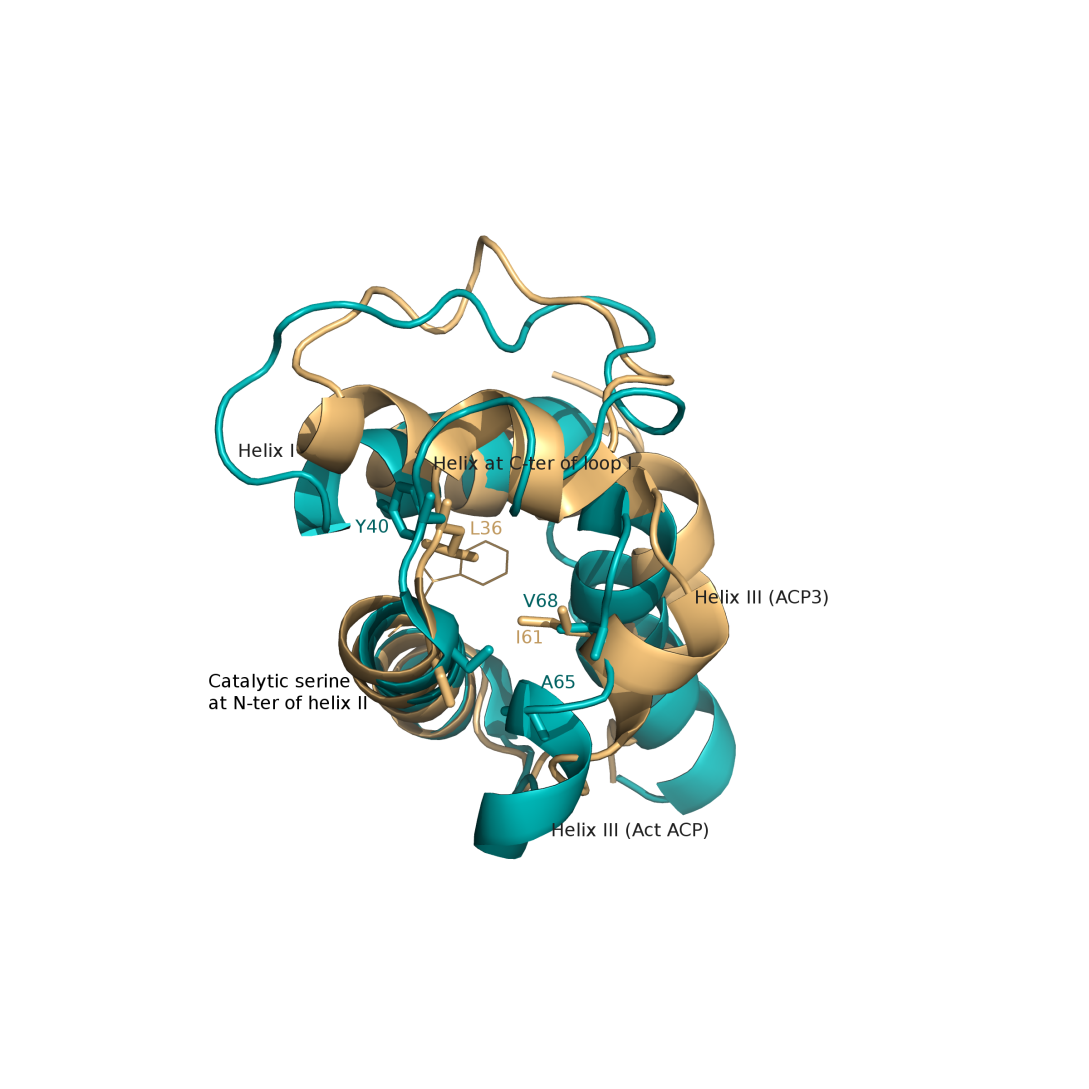 |
| --- | --- |
| **a** | **b** |
| 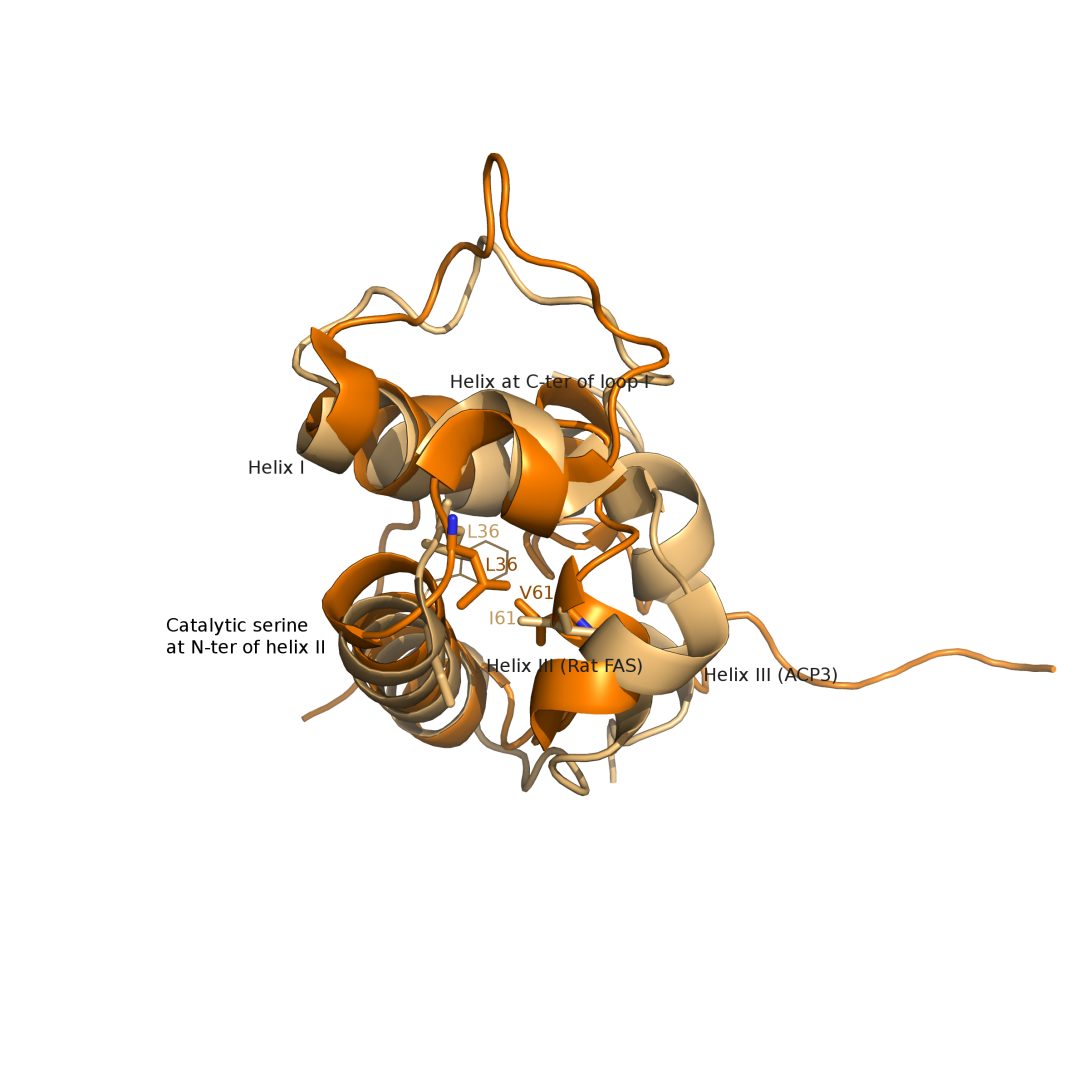 |  |
| **c** |  |

Fig U: Comparison between different ACPs of the orientation of the residue from III that packs into the core of the protein and the X residues of the GXDS motif. (a) AcpP from *E. coli* (in pink), and ACP3 (in beige) from MmpA of the mupirocin biosynthetic pathway (pdb IDs 2fac and 2l22). A59 on helix III of AcpP and I61 of ACP3 pack into the core of the protein, but due to the shifting of the position of helix III, relative to the other helices in the structure, I62 of AcpP actually spatially overlays more closely with I62 of ACP3, albeit with the sidechain from AcpP oriented not to occlude the channel. W44 of ACP3 is shown as lines, and is characteristic of ACPs that are involved in beta-branched modifications to the polyketide chain. A34 and L36, shown as stick representation, are the X residue of the GXDS motif. I61 interacts with helices II and IV and loop I, whereas Y64 (not shown), the only other residue from helix III of ACP3 that has any buried side chain, interacts only with helices III and IV. A59 of AcpP contacts the phosphopantetheine sequestered into the core of the four helix bundle and residues on helices II and II and the loop N-terminal to helix III (V43, I54, and D56, respectively, not shown). E58, the only other residue from helix II of AcpP that makes intra-protein interactions, interacts with helix IV (Y71) and helix III (P55, I54), but is still largely surface exposed. (b) Act ACP residue A65 (cyan; pdb ID 2k0x) packs from helix III into the core, the same as ACP3:I61 and AcpP:A59. I61 actually aligns better in the three dimensional structure with V68. A65 is the only residue in helix III that is totally buried, its side chain interacting with residues from helices II and IV. V64 from helix III (not shown) is partially buried and interacts with residues from helix IV and the loops before and after helix III. (c) ACP from type I FAS from rat (orange; pdb ID 2png), showing ACP3:I61 and V61, the residue of the FAS ACP that packs from helix III into the ACP’s core via side chain to side chain contact with residues from helix II and helix IV. ACP3:I61 and V61superpose in three dimensional space even though each ACP orients its helix III differently. V61 is the only residue from helix III of the FAS ACP that is totally buried, although the side chain of I58 contacts residues in helix II. All structures were superposed on the first domain of 2l22 using pymol’s align function with a restriction that only pairs of residues with a backbone RMSD of 1Å or less be included in the final fitting process. Figures were generated with pymol.


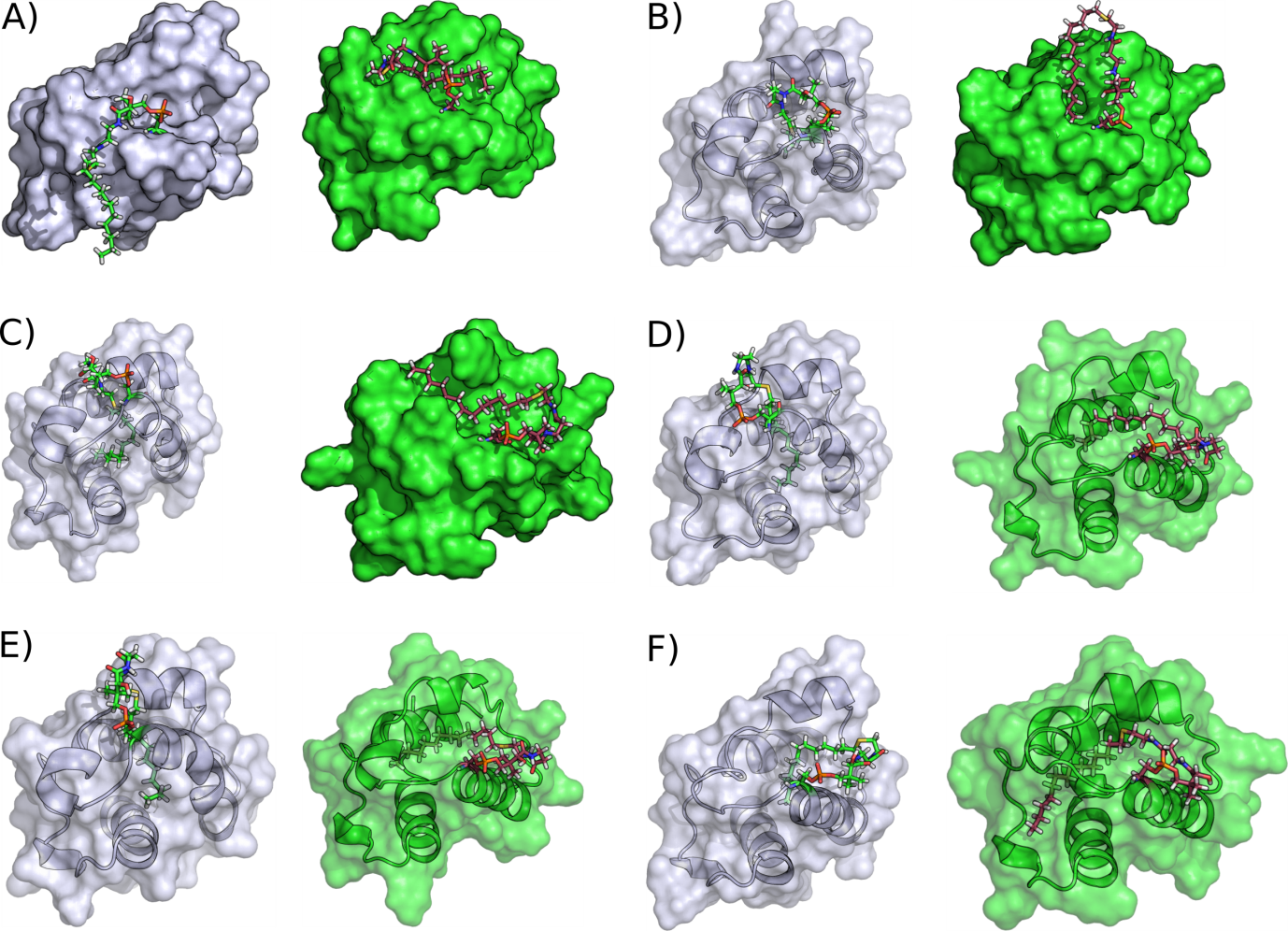


**Fig V: Comparison of the progression of substrate binding and the substrate binding modes of 14C AcpP WT (white) and 14C ACP3 L36A-W44L-I61A (green) during the course of their simulations.** The structures shown for the 14C ACP3 L36A-W44L-I61A structure are the same as in the Fig 8 b to g, in the main text, with the equivalent time points used for 14C AcpP WT frames (A=16.1 ns; B=82.48 ns; C=125.2 ns; D=155.05 ns; E=156.48 ns; F=200.00 ns), thus each pair does not necessarily show the same binding mode, but rather allow comparison of how the two simulations progress. The structure shown for AcpP are different snapshots from those shown in Fig. 5 of the main article. Panels E and F of this figure are indicative of how the ligand moves within the AcpP tunnel during the latter part of the simulation.


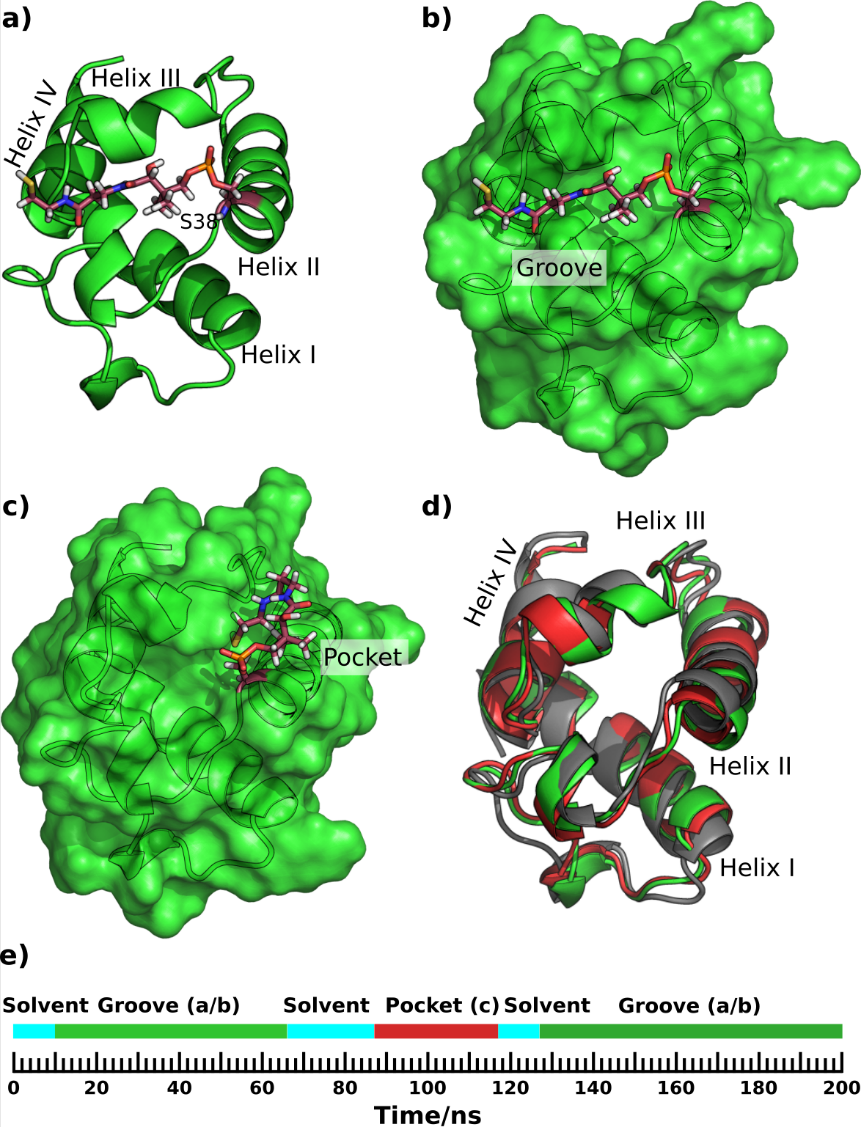


**Fig W: Substrate-bound conformations during a 200 ns simulation of holo ACP3.** a) & b) Cartoon & surface representations with the phosphopantetheine (stick representation) lying flat in a groove bounded by helix III, part of the loop connecting helix I and II (R30, F31, L32, E33, L34, G35, L36, D37; numbering is as PDB id 2L22, Figure S5) and the N-terminus of helix II. c) Phosphopantetheine diving into a small pocket formed by the N-terminus of helix II and helix III. d) Variation in helix position during the simulation; starting position in grey, snapshot shown in a) & b) in green and c) in red. e) Timeline of the simulation showing the occurrence and duration of the different conformations. The letters a, b, and c accord with the Figure panel labels. The orientation is the same in all the sub-figures.


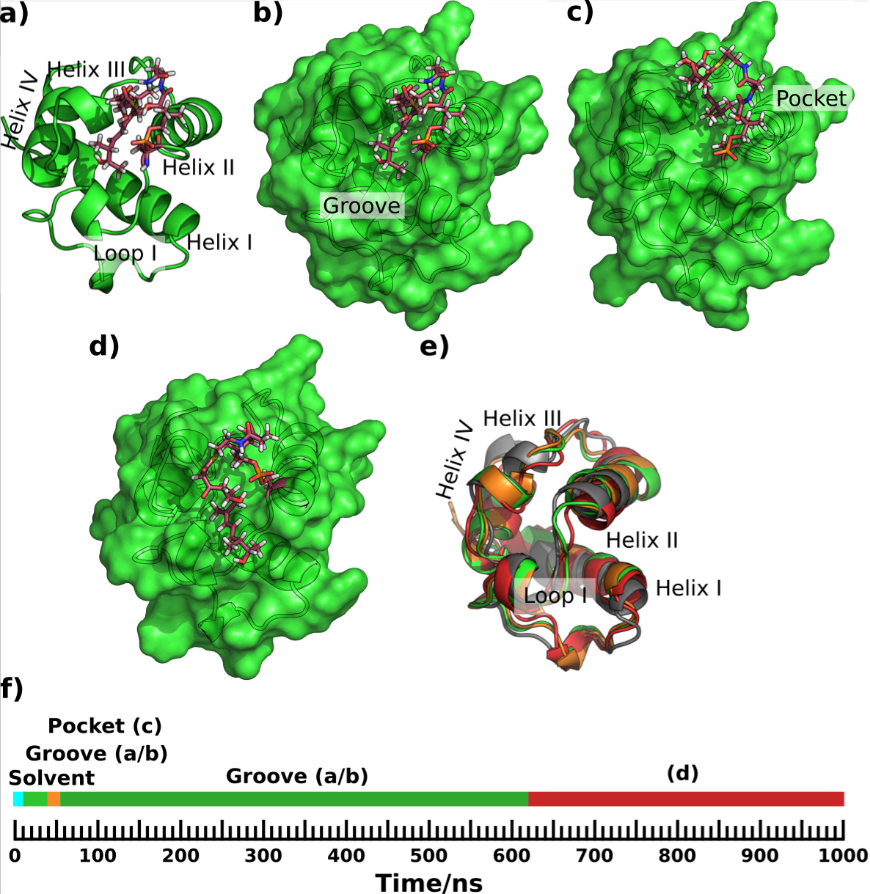


**Fig X:** **Cognate substrate binding to the surface groove during a 1 µs simulation of acyl ACP3 WT.** a) & b) Cartoon & surface drawing respectively of the acyl ACP3 WT with the acyl chain lying flat in the groove – residues within 5 Å of the acyl chain and ppt are F31, L32, L36, D37, V39, I40, A41, A42, A58, A59, G60, Y62 and P65. c) The acyl chain in a small pocket formed by the N-terminus of helix II and helix III. d) The acyl chain passing over loop I. e) Comparison of the variation between structures; starting position in grey, configuration a/b in green, c in orange and d in red. f) Timeline of the simulation. The orientation of all sub-figures is equivalent and they appear in the same order as in the 1 µs trajectory.


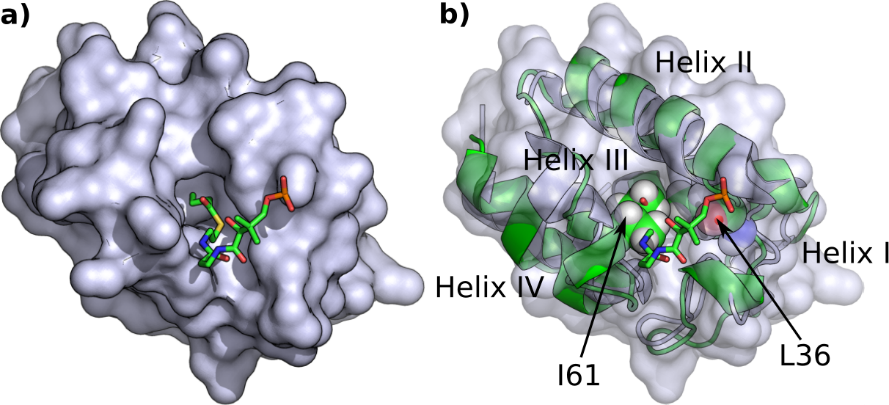


**Fig SY:** **Comparison of FAS AcpP and ACP3 structures.** a) Surface rendering of AcpP with the ligand (shown as sticks) buried in the cavity. b) Superimposed AcpP (surface) and ACP3 (cartoon) structures with the I61 & L36 (spheres) shown as a blockage at the cavity opening.

**S2.7 Modified forcefield parameters implemented in AMBER99SB-ILDN forcefield in Gromacs format.**

**Residue Topology Parameters (aminoacids.rtp) for:**

**Phosphopantetheine**

[ SPT ]

[ atoms ]

N N -0.41570 1

H H 0.27190 2

CA CT -0.08800 3

HA H1 0.15590 4

CB CT 0.03140 5

HB1 H1 0.08540 6

HB2 H1 0.08540 7

OG OS -0.43570 8

PD p5r 1.04280 9

OE or -0.73120 10

OH or -0.73120 11

OZ osr -0.40080 12

CQ c3r -0.02100 13

HQ1 h1r 0.10220 14

HQ2 h1r 0.10220 15

CI c3r 0.12870 16

CIA c3r -0.15840 17

HIA1 hcr 0.03550 18

HIA2 hcr 0.03550 19

HIA3 hcr 0.03550 20

CIB c3r -0.15840 21

HIB1 hcr 0.03550 22

HIB2 hcr 0.03550 23

HIB3 hcr 0.03550 24

CIG c3r -0.00210 25

HIG h1r 0.16460 26

OID ohr -0.58750 27

HID hor 0.40570 28

CIE cr 0.38430 29

OIZ or -0.49960 30

NH nr -0.28870 31

HH hnr 0.25640 32

CIQ c3r 0.03840 33

HIQ1 h1r 0.05000 34

HIQ2 h1r 0.05000 35

CK c3r -0.17820 36

HK1 hcr 0.07800 37

HK2 hcr 0.07800 38

CKA cr 0.51920 39

OKB or -0.57540 40

NKG nr -0.40580 41

HKG hnr 0.27300 42

CKD c3r 0.02980 43

HKD1 h1r 0.06100 44

HKD2 h1r 0.06100 45

CKE c3r -0.00750 46

HKE1 h1r 0.09690 47

HKE2 h1r 0.09690 48

SKZ shr -0.42300 49

HKZ hsr 0.21670 50

C C 0.59730 51

O O -0.56790 52

[ bonds ]

N H

N CA

CA HA

CA CB

CA C

CB HB1

CB HB2

CB OG

OG PD

PD OE

PD OH

PD OZ

OZ CQ

CQ HQ1

CQ HQ2

CQ CI

CI CIA

CI CIB

CI CIG

CIA HIA1

CIA HIA2

CIA HIA3

CIB HIB1

CIB HIB2

CIB HIB3

CIG HIG

CIG OID

OID HID

CIG CIE

CIE OIZ

CIE NH

NH HH

NH CIQ

CIQ HIQ1

CIQ HIQ2

CIQ CK

CK HK1

CK HK2

CK CKA

CKA OKB

CKA NKG

NKG HKG

NKG CKD

CKD HKD1

CKD HKD2

CKD CKE

CKE HKE1

CKE HKE2

CKE SKZ

SKZ HKZ

C O

-C N

[ impropers ]

-C CA N H

CA +N C O

CIE CIQ NH HH

CIG NH CIE OIZ

CKA CKD NKG HKG

CK NKG CKA OKB

**Unbranched monic acid attached to phosphopantetheine (ACP3 cognate substrate)**

[ SPM ]

[ atoms ]

N N -0.4157 1

H H 0.2719 2

CA CT -0.0880 3

HA H1 0.1559 4

CB CT 0.0314 5

HB1 H1 0.0854 6

HB2 H1 0.0854 7

OG OS -0.4357 8

PD p5r 1.0428 9

OE or -0.7312 10

OH or -0.7312 11

OZ osr -0.4008 12

CQ c3r -0.0210 13

HQ1 h1r 0.1022 14

HQ2 h1r 0.1022 15

CI c3r 0.1287 16

CIA c3r -0.1584 17

HIA1 hcr 0.0355 18

HIA2 hcr 0.0355 19

HIA3 hcr 0.0355 20

CIB c3r -0.1584 21

HIB1 hcr 0.0355 22

HIB2 hcr 0.0355 23

HIB3 hcr 0.0355 24

CIG c3r -0.0021 25

HIG h1r 0.1646 26

OID ohr -0.5875 27

HID hor 0.4057 28

CIE cr 0.3843 29

OIZ or -0.4996 30

NH nr -0.2887 31

HH hnr 0.2564 32

CIQ c3r 0.0384 33

HIQ1 h1r 0.0500 34

HIQ2 h1r 0.0500 35

CK c3r -0.1782 36

HK1 hcr 0.0780 37

HK2 hcr 0.0780 38

CKA cr 0.6244 39

OKB or -0.5521 40

NKG nr -0.5468 41

HKG hnr 0.2730 42

CKD c3r -0.1108 43

HKD1 h1r 0.1309 44

HKD2 h1r 0.1309 45

CKE c3r 0.0796 46

HKE1 h1r 0.0565 47

HKE2 h1r 0.0565 48

SKZ ssr -0.278 49

CKH cr 0.4936 50

OKQ or -0.4762 51

CX c3r -0.0376 52

HX1 hcr 0.0292 53

HX2 hcr 0.0292 54

CXA cr 0.5017 55

OXB or -0.4804 56

CXG c3r -0.0339 57

HXG1 hcr 0.0325 58

HXG2 hcr 0.0325 59

CXD c3r 0.1369 60

HXD h1r 0.1344 61

OXE ohr -0.6552 62

HXE hor 0.4331 63

CXZ c3r -0.0199 64

HXZ h1r 0.1196 65

OXH ohr -0.6166 66

HXH hor 0.3962 67

CXQ c3r 0.1625 68

HXQ h1r 0.1158 69

OM ohr -0.6569 70

HM hor 0.4266 71

CMA c2r 0.1042 72

CMB c3r -0.1920 73

HMB1 hcr 0.0639 74

HMB2 hcr 0.0639 75

HMB3 hcr 0.0639 76

CMG cer -0.2584 77

HMG har 0.1528 78

CMD cer -0.1381 79

HMD har 0.1190 80

CME c2r -0.1661 81

HME har 0.1142 82

CMZ c3r 0.0367 83

HMZ hcr 0.0647 84

CMH c3r -0.1807 85

HMH1 hcr 0.0480 86

HMH2 hcr 0.0480 87

HMH3 hcr 0.0480 88

CMQ c3r 0.2814 89

HMQ h1r 0.0165 90

ON ohr -0.6655 91

HN hor 0.4035 92

CNA c3r -0.2053 93

HNA1 hcr 0.0630 94

HNA2 hcr 0.0630 95

HNA3 hcr 0.0630 96

C C 0.5973 97

O O -0.5679 98

[ bonds ]

C CA

CA HA

CA N

CA CB

N H

CB HB1

CB HB2

CB OG

OG PD

PD OE

PD OH

PD OZ

OZ CQ

CQ HQ1

CQ HQ2

CQ CI

CI CIA

CI CIB

CI CIG

CIA HIA1

CIA HIA2

CIA HIA3

CIB HIB1

CIB HIB2

CIB HIB3

CIG HIG

CIG OID

CIG CIE

OID HID

CIE OIZ

CIE NH

NH HH

NH CIQ

CIQ HIQ1

CIQ HIQ2

CIQ CK

CK HK1

CK HK2

CK CKA

CKA OKB

CKA NKG

NKG HKG

NKG CKD

CKD HKD1

CKD HKD2

CKD CKE

CKE HKE1

CKE HKE2

CKE SKZ

SKZ CKH

CKH OKQ

CKH CX

CX HX1

CX HX2

CX CXA

CXA OXB

CXA CXG

CXG HXG1

CXG HXG2

CXG CXD

CXD HXD

CXD OXE

CXD CXZ

OXE HXE

CXZ HXZ

CXZ OXH

CXZ CXQ

OXH HXH

CXQ HXQ

CXQ OM

CXQ CMA

OM HM

CMA CMB

CMA CMG

CMB HMB1

CMB HMB2

CMB HMB3

CMG HMG

CMG CMD

CMD HMD

CMD CME

CME HME

CME CMZ

CMZ HMZ

CMZ CMH

CMZ CMQ

CMH HMH1

CMH HMH2

CMH HMH3

CMQ HMQ

CMQ ON

CMQ CNA

ON HN

CNA HNA1

CNA HNA2

CNA HNA3

C O

-C N

[ impropers ]

-C CA N H

CA +N C O

CIG NH CIE OIZ

CIE CIQ NH HH

CK NKG CKA OKB

CKA CKD NKG HKG

CX OKQ CKH SKZ

CX CXG CXA OXB

CXQ CMB CMA CMG

CMA CMD CMG HMG

CME CMG CMD HMD

CMZ CMD CME HME

**Mupirocin intermediate attached to phosphopantetheine (ACP2 cognate substrate)**

[ SPB ]

[ atoms ]

N N -0.4157 1

H H 0.2719 2

CA CT -0.0880 3

HA H1 0.1559 4

CB CT 0.0314 5

HB1 H1 0.0854 6

HB2 H1 0.0854 7

OG OS -0.4357 8

PD p5r 1.0428 9

OE or -0.7312 10

OH or -0.7312 11

OZ osr -0.4008 12

CQ c3r -0.0210 13

HQ1 h1r 0.1022 14

HQ2 h1r 0.1022 15

CI c3r 0.1287 16

CIA c3r -0.1584 17

HIA1 hcr 0.0355 18

HIA2 hcr 0.0355 19

HIA3 hcr 0.0355 20

CIB c3r -0.1584 21

HIB1 hcr 0.0355 22

HIB2 hcr 0.0355 23

HIB3 hcr 0.0355 24

CIG c3r -0.0021 25

HIG h1r 0.1646 26

OID ohr -0.5875 27

HID hor 0.4057 28

CIE cr 0.3843 29

OIZ or -0.4996 30

NH nr -0.2887 31

HH hnr 0.2564 32

CIQ c3r 0.0384 33

HIQ1 h1r 0.0500 34

HIQ2 h1r 0.0500 35

CK c3r -0.1134 36

HK1 hcr 0.0353 37

HK2 hcr 0.0353 38

CKA cr 0.6244 39

OKB or -0.5521 40

NKG nr -0.5991 41

HKG hnr 0.3539 42

CKD c3r 0.0208 43

HKD1 h1r 0.0485 44

HKD2 h1r 0.0485 45

CKE c3r 0.0518 46

HKE1 h1r 0.0613 47

HKE2 h1r 0.0613 48

SKZ ssr -0.2812 49

CXA cr 0.5541 50

OXB or -0.4720 51

CXG c3r -0.1453 52

HXG1 hcr 0.0647 53

HXG2 hcr 0.0647 54

CXD c3r 0.2284 55

HXD h1r 0.1019 56

OXE ohr -0.5954 57

HXE hor 0.4071 58

CXZ c3r 0.0214 59

HXZ h1r 0.1137 60

OXH ohr -0.6254 61

HXH hor 0.3978 62

CXQ c3r 0.1110 63

HXQ h1r 0.1173 64

OM ohr -0.6240 65

HM hor 0.3929 66

CMA c2r 0.1146 67

CMB c3r -0.1914 68

HMB1 hcr 0.0624 69

HMB2 hcr 0.0624 70

HMB3 hcr 0.0624 71

CMG cer -0.2579 72

HMG har 0.1484 73

CMD cer -0.1291 74

HMD har 0.1234 75

CME c2r -0.1780 76

HME har 0.1196 77

CMZ c3r 0.0148 78

HMZ hcr 0.0736 79

CMH c3r -0.1712 80

HMH1 hcr 0.0441 81

HMH2 hcr 0.0441 82

HMH3 hcr 0.0441 83

CMQ c3r 0.3047 84

HMQ h1r 0.0121 85

ON ohr -0.6719 86

HN hor 0.4045 87

CNA c3r -0.2237 88

HNA1 hcr 0.0668 89

HNA2 hcr 0.0668 90

HNA3 hcr 0.0668 91

C C 0.5973 92

O O -0.5679 93

[ bonds ]

C CA

CA HA

CA N

CA CB

N H

CB HB1

CB HB2

CB OG

OG PD

PD OE

PD OH

PD OZ

OZ CQ

CQ HQ1

CQ HQ2

CQ CI

CI CIA

CI CIB

CI CIG

CIA HIA1

CIA HIA2

CIA HIA3

CIB HIB1

CIB HIB2

CIB HIB3

CIG HIG

CIG OID

CIG CIE

OID HID

CIE OIZ

CIE NH

NH HH

NH CIQ

CIQ HIQ1

CIQ HIQ2

CIQ CK

CK HK1

CK HK2

CK CKA

CKA OKB

CKA NKG

NKG HKG

NKG CKD

CKD HKD1

CKD HKD2

CKD CKE

CKE HKE1

CKE HKE2

CKE SKZ

SKZ CXA

CXA OXB

CXA CXG

CXG HXG1

CXG HXG2

CXG CXD

CXD HXD

CXD OXE

CXD CXZ

OXE HXE

CXZ HXZ

CXZ OXH

CXZ CXQ

OXH HXH

CXQ HXQ

CXQ OM

CXQ CMA

OM HM

CMA CMB

CMA CMG

CMB HMB1

CMB HMB2

CMB HMB3

CMG HMG

CMG CMD

CMD HMD

CMD CME

CME HME

CME CMZ

CMZ HMZ

CMZ CMH

CMZ CMQ

CMH HMH1

CMH HMH2

CMH HMH3

CMQ HMQ

CMQ ON

CMQ CNA

ON HN

CNA HNA1

CNA HNA2

CNA HNA3

C O

-C N

[ impropers ]

-C CA N H

CA +N C O

CIG NH CIE OIZ

CIE CIQ NH HH

CK NKG CKA OKB

CKA CKD NKG HKG

CXG OXB CKA SKZ

CXQ CMB CMA CMG

CMA CMD CMG HMG

CME CMG CMD HMD

CMZ CMD CME HME

**Fully saturated carbon chain attached to phosphopantetheine**

[ SPD ]

[ atoms ]

N N -0.4157 1

H H 0.2719 2

CA CT -0.0880 3

HA H1 0.1559 4

CB CT 0.0314 5

HB1 H1 0.0854 6

HB2 H1 0.0854 7

OG OS -0.4357 8

PD p5r 1.0428 9

OE or -0.7312 10

OH or -0.7312 11

OZ osr -0.4008 12

CQ c3r -0.0210 13

HQ1 h1r 0.1022 14

HQ2 h1r 0.1022 15

CI c3r 0.1287 16

CIA c3r -0.1584 17

HIA1 hcr 0.0355 18

HIA2 hcr 0.0355 19

HIA3 hcr 0.0355 20

CIB c3r -0.1584 21

HIB1 hcr 0.0355 22

HIB2 hcr 0.0355 23

HIB3 hcr 0.0355 24

CIG c3r -0.0021 25

HIG h1r 0.1646 26

OID ohr -0.5875 27

HID hor 0.4057 28

CIE cr 0.3843 29

OIZ or -0.4996 30

NH nr -0.2887 31

HH hnr 0.2564 32

CIQ c3r 0.0384 33

HIQ1 h1r 0.0500 34

HIQ2 h1r 0.0500 35

CK c3r -0.1782 36

HK1 hcr 0.0746 37

HK2 hcr 0.0746 38

CKA cr 0.5692 39

OKB or -0.5754 40

NKG nr -0.4058 41

HKG hnr 0.2730 42

CKD c3r 0.0298 43

HKD1 h1r 0.0610 44

HKD2 h1r 0.0610 45

CKE c3r -0.0075 46

HKE1 h1r 0.0969 47

HKE2 h1r 0.0969 48

SKZ ssr -0.4230 49

CKH c3r -0.1364 50

HKH1 hcr 0.0980 51

HKH2 hcr 0.0980 52

CX c3r 0.0624 53

HX1 hcr 0.0311 54

HX2 hcr 0.0311 55

CXA c3r -0.0179 56

HXA1 hcr -0.0035 57

HXA2 hcr -0.0035 58

CXG c3r 0.0472 59

HXG1 hcr -0.0190 60

HXG2 hcr -0.0190 61

CXD c3r 0.0036 62

HXD1 hcr -0.0024 63

HXD2 hcr -0.0024 64

CXZ c3r 0.0074 65

HXZ1 hcr -0.0008 66

HXZ2 hcr -0.0008 67

CXQ c3r 0.0074 68

HXQ1 hcr -0.0008 69

HXQ2 hcr -0.0008 70

CMA c3r 0.0036 71

HMA1 hcr -0.0024 72

HMA2 hcr -0.0024 73

CMG c3r -0.0198 74

HMG1 hcr 0.0053 75

HMG2 hcr 0.0053 76

CMD c3r 0.0074 77

HMD1 hcr -0.0008 78

HMD2 hcr -0.0008 79

CME c3r -0.0091 80

HME1 hcr -0.0008 81

HME2 hcr -0.0008 82

CMZ c3r 0.0190 83

HMZ1 hcr -0.0013 84

HMZ2 hcr -0.0013 85

CMQ c3r 0.0329 86

HMQ1 hcr -0.0053 87

HMQ2 hcr -0.0053 88

CNA c3r -0.0579 89

HNA1 hcr 0.0097 90

HNA2 hcr 0.0097 91

HNA3 hcr 0.0097 92

C C 0.5973 93

O O -0.5679 94

[ bonds ]

C CA

CA HA

CA N

CA CB

N H

CB HB1

CB HB2

CB OG

OG PD

PD OE

PD OH

PD OZ

OZ CQ

CQ HQ1

CQ HQ2

CQ CI

CI CIA

CI CIB

CI CIG

CIA HIA1

CIA HIA2

CIA HIA3

CIB HIB1

CIB HIB2

CIB HIB3

CIG HIG

CIG OID

CIG CIE

OID HID

CIE OIZ

CIE NH

NH HH

NH CIQ

CIQ HIQ1

CIQ HIQ2

CIQ CK

CK HK1

CK HK2

CK CKA

CKA OKB

CKA NKG

NKG HKG

NKG CKD

CKD HKD1

CKD HKD2

CKD CKE

CKE HKE1

CKE HKE2

CKE SKZ

SKZ CKH

CKH HKH1

CKH HKH2

CKH CX

CX HX1

CX HX2

CX CXA

CXA HXA1

CXA HXA2

CXA CXG

CXG HXG1

CXG HXG2

CXG CXD

CXD HXD1

CXD HXD2

CXD CXZ

CXZ HXZ1

CXZ HXZ2

CXZ CXQ

CXQ HXQ1

CXQ HXQ2

CXQ CMA

CMA HMA1

CMA HMA2

CMA CMG

CMG HMG1

CMG HMG2

CMG CMD

CMD HMD1

CMD HMD2

CMD CME

CME HME1

CME HME2

CME CMZ

CMZ HMZ1

CMZ HMZ2

CMZ CMQ

CMQ HMQ1

CMQ HMQ2

CMQ CNA

CNA HNA1

CNA HNA2

CNA HNA3

C O

-C N

[ impropers ]

-C CA N H

CA +N C O

**Hydrogen Database (aminoacids.hdb) For:**

**Phosphopantetheine**

SPT 15

1 1 H N -C CA

1 5 HA CA N CB C

2 6 HB CB CA OG

2 6 HQ CQ OZ CI

3 4 HIA CIA CI CQ

3 4 HIB CIB CI CQ

1 5 HIG CIG CI CIE OID

1 2 HID OID CIG CI

1 1 HH NH CIE CIQ

2 6 HIQ CIQ NH CK

2 6 HK CK CIQ CKA

1 1 HKG NKG CKA CKD

2 6 HKD CKD NKG CKE

2 6 HKE CKE CKD SKZ

1 2 HKZ SKZ CKE CKD

**Unbranched monic acid attached to phosphopantetheine (ACP3 cognate substrate)**

SPM 31

1 1 H N -C CA

1 5 HA CA N CB C

2 6 HB CB CA OG

2 6 HQ CQ OZ CI

3 4 HIA CIA CI CQ

3 4 HIB CIB CI CQ

1 5 HIG CIG CI CIE OID

1 2 HID OID CIG CI

1 1 HH NH CIE CIQ

2 6 HIQ CIQ NH CK

2 6 HK CK CIQ CKA

1 1 HKG NKG CKA CKD

2 6 HKD CKD NKG CKE

2 6 HKE CKE CKD SKZ

2 6 HX CX CKH CXA

2 6 HXG CXG CXA CXD

1 5 HXD CXD CXG CXZ OXE

1 2 HXE OXE CXD CXG

1 5 HXZ CXZ CXD CXQ OXH

1 2 HXH OXH CXZ CXD

1 5 HXQ CXQ CXZ CMA OM

1 2 HM OM CXQ CXZ

3 4 HMB CMB CMA CXQ

1 1 HMG CMG CMA CMD

1 1 HMD CMD CMG CME

1 1 HME CME CMD CMZ

1 5 HMZ CMZ CME CMQ CMH

3 4 HMH CMH CMZ CME

1 5 HMQ CMQ CMZ CNA ON

1 2 HN ON CMQ CMZ

3 4 HNA CNA CMQ CMZ

**Mupirocin intermediate attached to phosphopantetheine (ACP2 cognate substrate)**

SPB 30

1 1 H N -C CA

1 5 HA CA N CB C

2 6 HB CB CA OG

2 6 HQ CQ OZ CI

3 4 HIA CIA CI CQ

3 4 HIB CIB CI CQ

1 5 HIG CIG CI CIE OID

1 2 HID OID CIG CI

1 1 HH NH CIE CIQ

2 6 HIQ CIQ NH CK

2 6 HK CK CIQ CKA

1 1 HKG NKG CKA CKD

2 6 HKD CKD NKG CKE

2 6 HKE CKE CKD SKZ

2 6 HXG CXG CXA CXD

1 5 HXD CXD CXG CXZ OXE

1 2 HXE OXE CXD CXG

1 5 HXZ CXZ CXD CXQ OXH

1 2 HXH OXH CXZ CXD

1 5 HXQ CXQ CXZ CMA OM

1 2 HM OM CXQ CXZ

3 4 HMB CMB CMA CXQ

1 1 HMG CMG CMA CMD

1 1 HMD CMD CMG CME

1 1 HME CME CMD CMZ

1 5 HMZ CMZ CME CMQ CMH

3 4 HMH CMH CMZ CME

1 5 HMQ CMQ CMZ CNA ON

1 2 HN ON CMQ CMZ

3 4 HNA CNA CMQ CMZ

**Fully saturated carbon chain attached to phosphopantetheine**

SPD 28

1 1 H N -C CA

1 5 HA CA N CB C

2 6 HB CB CA OG

2 6 HQ CQ OZ CI

3 4 HIA CIA CI CQ

3 4 HIB CIB CI CQ

1 5 HIG CIG CI CIE OID

1 2 HID OID CIG CI

1 1 HH NH CIE CIQ

2 6 HIQ CIQ NH CK

2 6 HK CK CIQ CKA

1 1 HKG NKG CKA CKD

2 6 HKD CKD NKG CKE

2 6 HKE CKE CKD SKZ

2 6 HKH CKH SKZ CX

2 6 HX CX CKH CXA

2 6 HXA CXA CX CXG

2 6 HXG CXG CXA CXD

2 6 HXD CXD CXG CXZ

2 6 HXZ CXZ CXD CXQ

2 6 HXQ CXQ CXZ CMA

2 6 HMA CMA CXQ CMG

2 6 HMG CMG CMA CMD

2 6 HMD CMD CMG CME

2 6 HME CME CMD CMZ

2 6 HMZ CMZ CME CMQ

2 6 HMQ CMQ CMZ CNA

3 4 HNA CNA CMQ CMZ

**Atom Types (atomtypes.atp)**

cr 12.01000 ; Sp2 C carbonyl group

c1r 12.01000 ; Sp C

c2r 12.01000 ; Sp2 C

c3r 12.01000 ; Sp3 C

car 12.01000 ; Sp2 C in pure aromatic systems

cpr 12.01000 ; Head Sp2 C that connect two rings in biphenyl sys.

cqr 12.01000 ; Head Sp2 C that connect two rings in biphenyl sys. identical to cp

ccr 12.01000 ; Sp2 carbons in non-pure aromatic systems

cdr 12.01000 ; Sp2 carbons in non-pure aromatic systems, identical to cc

cer 12.01000 ; Inner Sp2 carbons in conjugated systems

cfr 12.01000 ; Inner Sp2 carbons in conjugated systems, identical to ce

cgr 12.01000 ; Inner Sp carbons in conjugated systems

chr 12.01000 ; Inner Sp carbons in conjugated systems, identical to cg

cxr 12.01000 ; Sp3 carbons in triangle systems

cyr 12.01000 ; Sp3 carbons in square systems

cur 12.01000 ; Sp2 carbons in triangle systems

cvr 12.01000 ; Sp2 carbons in square systems

czr 12.01000 ; Sp2 carbon in guanidine group

h1r 1.00800 ; H bonded to aliphatic carbon with 1 electrwd. group

h2r 1.00800 ; H bonded to aliphatic carbon with 2 electrwd. group

h3r 1.00800 ; H bonded to aliphatic carbon with 3 electrwd. group

h4r 1.00800 ; H bonded to non-sp3 carbon with 1 electrwd. group

h5r 1.00800 ; H bonded to non-sp3 carbon with 2 electrwd. group

har 1.00800 ; H bonded to aromatic carbon

hcr 1.00800 ; H bonded to aliphatic carbon without electrwd. group

hnr 1.00800 ; H bonded to nitrogen atoms

hor 1.00800 ; Hydroxyl group

hpr 1.00800 ; H bonded to phosphate

hsr 1.00800 ; Hydrogen bonded to sulphur

hwr 1.00800 ; Hydrogen in water

hxr 1.00800 ; H bonded to C next to positively charged group

fr 19.00000 ; Fluorine

clr 35.45000 ; Chlorine

brr 79.90000 ; Bromine

ir 126.90000 ; Iodine

nr 14.01000 ; Sp2 nitrogen in amide groups

n1r 14.01000 ; Sp N

n2r 14.01000 ; aliphatic Sp2 N with two connected atoms

n3r 14.01000 ; Sp3 N with three connected atoms

n4r 14.01000 ; Sp3 N with four connected atoms

nar 14.01000 ; Sp2 N with three connected atoms

nbr 14.01000 ; Sp2 N in pure aromatic systems

ncr 14.01000 ; Sp2 N in non-pure aromatic systems

ndr 14.01000 ; Sp2 N in non-pure aromatic systems, identical to nc

ner 14.01000 ; Inner Sp2 N in conjugated systems

nfr 14.01000 ; Inner Sp2 N in conjugated systems, identical to ne

nhr 14.01000 ; Amine N connected one or more aromatic rings

nor 14.01000 ; Nitro N

or 16.00000 ; Oxygen with one connected atom

ohr 16.00000 ; Oxygen in hydroxyl group

osr 16.00000 ; Ether and ester oxygen

owr 16.00000 ; Oxygen in water

p2r 30.97000 ; Phosphate with two connected atoms

p3r 30.97000 ; Phosphate with three connected atoms, such as PH3

p4r 30.97000 ; Phosphate with three connected atoms, such as O=P(CH3)2

p5r 30.97000 ; Phosphate with four connected atoms, such as O=P(OH)3

pbr 30.97000 ; Sp2 P in pure aromatic systems

pcr 30.97000 ; Sp2 P in non-pure aromatic systems

pdr 30.97000 ; Sp2 P in non-pure aromatic systems, identical to pc

per 30.97000 ; Inner Sp2 P in conjugated systems

pfr 30.97000 ; Inner Sp2 P in conjugated systems, identical to pe

pxr 30.97000 ; Special p4 in conjugated systems

pyr 30.97000 ; Special p5 in conjugated systems

sr 32.06000 ; S with one connected atom

s2r 32.06000 ; S with two connected atom, involved at least one double bond

s4r 32.06000 ; S with three connected atoms

s6r 32.06000 ; S with four connected atoms

shr 32.06000 ; Sp3 S connected with hydrogen

ssr 32.06000 ; Sp3 S in thio-ester and thio-ether

sxr 32.06000 ; Special s4 in conjugated systems

syr 32.06000 ; Special s6 in conjugated systems

**Force Field Bonded Parameters (ffbonded.itp)**

[ bondtypes ]

; i j func b0 kb

owr hwr 1 0.09572 462750.4 ; TIP3P_Water 1

brr brr 1 0.25420 103093.8 ; SOURCE1 4 0.0000

brr c1r 1 0.17870 295139.4 ; SOURCE2 4 0.0024

brr c2r 1 0.18830 233216.2 ; SOURCE1 31 0.0000

brr cr 1 0.19460 201083.0 ; SOURCE2 2 0.0285

brr c3r 1 0.19660 192045.6 ; SOURCE1 100 0.0000

brr car 1 0.18970 225601.3 ; SOURCE1 127 0.0058

brr ccr 1 0.18847 232295.7 ; SOURCE4 39 0.0068

brr cxr 1 0.19100 218739.5 ; SOURCE1 8 0.0000

brr ir 1 0.26710 119160.3 ; SOURCE1 2 0.0245

brr n1r 1 0.18600 276478.7 ; SOUECE3 1

brr n2r 1 0.20380 183259.2 ; SOURCE3 5 0.1082

brr nr 1 0.18730 267943.4 ; SOURCE3 4 0.0046

brr n3r 1 0.19520 222505.1 ; SOURCE3 2 0.0000

brr n4r 1 0.19260 236312.3 ; SOURCE3 3 0.0013

brr nar 1 0.20020 198572.6 ; SOURCE3 7 0.2156

brr nhr 1 0.19440 226689.1 ; SOURCE3 1 0.0000

brr nor 1 0.21010 159828.8 ; SOURCE3 1 0.0000

brr or 1 0.18000 233383.5 ; SOUECE3 1

brr ohr 1 0.18660 198489.0 ; SOURCE3 1 0.0000

brr osr 1 0.18870 188782.1 ; SOURCE3 2 0.0000

brr p2r 1 0.22100 145854.2 ; SOURCE3 9 0.0510

brr p3r 1 0.22310 139745.6 ; SOURCE3 3 0.0101

brr p4r 1 0.21710 157987.8 ; SOUECE3 1

brr p5r 1 0.21960 150038.2 ; SOURCE3 3 0.0099

brr sr 1 0.22200 142758.1 ; SOUECE3 1

brr s4r 1 0.23410 112382.2 ; SOURCE3 1 0.0000

brr s6r 1 0.22140 144515.4 ; SOURCE3 3 0.0443

brr shr 1 0.22090 145937.9 ; SOURCE3 1 0.0000

brr ssr 1 0.22030 147778.9 ; SOURCE3 3 0.0035

c1r c1r 1 0.11810 825252.2 ; SOURCE1 265 0.0031

c1r c2r 1 0.13070 523000.0 ; SOURCE1 18 0.0000

c1r c3r 1 0.14700 308193.4 ; SOURCE1 215 0.0017

c1r car 1 0.14400 338150.9 ; SOUECE3 1

c1r cer 1 0.13153 508272.3 ; SOURCE4 6 0.0086

c1r cgr 1 0.12220 707765.4 ; SOURCE3 22 0.0101

c1r chr 1 0.12220 707765.4 ; SOURCE3 22 same_as_c1-cg

c1r clr 1 0.16310 351205.0 ; SOURCE2 6 0.0050

c1r cxr 1 0.14440 333966.9 ; SOURCE1 38 0.0000

c1r fr 1 0.12700 392793.9 ; SOURCE2 2 0.0085

c1r har 1 0.10660 314553.1 ; SOURCE3 63 0.0035

c1r hcr 1 0.10600 322670.1 ; SOUECE3 1

c1r ir 1 0.19890 266771.8 ; SOURCE2 4 0.0032

c1r n1r 1 0.11380 848933.6 ; SOURCE1 170 0.0055

c1r n2r 1 0.12100 644168.6 ; SOURCE3 5 0.0115

c1r n3r 1 0.13920 342920.6 ; SOURCE2 1 0.0000

c1r n4r 1 0.14170 316477.8 ; SOURCE3 3 0.0032

c1r nr 1 0.13300 420910.4 ; SOUECE3 1

c1r nar 1 0.13620 378233.6 ; SOURCE3 8 0.0034

c1r ner 1 0.11986 672201.4 ; SOURCE4 10 0.0088

c1r nfr 1 0.11986 672201.4 ; SOURCE4 10 same_as_c1-ne

c1r nhr 1 0.13408 405848.0 ; SOURCE4 11 0.0037

c1r nor 1 0.14050 328862.4 ; SOURCE3 3 0.0005

c1r or 1 0.11660 650193.6 ; SOURCE2 9 0.0052

c1r ohr 1 0.13260 364510.1 ; SOURCE3 1 0.0000

c1r osr 1 0.13250 365765.3 ; SOURCE3 3 0.0148

c1r p2r 1 0.17700 242086.2 ; SOUECE3 1

c1r p3r 1 0.17900 230203.7 ; SOUECE3 1

c1r p4r 1 0.17900 230203.7 ; SOUECE3 1

c1r p5r 1 0.17530 252881.0 ; SOURCE3 2 0.0000

c1r s2r 1 0.15950 343088.0 ; SOURCE3 1 0.0000

c1r sr 1 0.16300 311122.2 ; SOURCE1 14 0.0000

c1r s4r 1 0.17460 228362.7 ; SOURCE3 2 0.0000

c1r s6r 1 0.17220 243006.7 ; SOURCE3 2 0.0000

c1r shr 1 0.16800 271541.6 ; SOUECE3 1

c1r ssr 1 0.16790 272294.7 ; SOURCE1 10 0.0000

c2r c2r 1 0.13240 493461.0 ; SOURCE1 974 0.0096

c2r c3r 1 0.15080 274721.4 ; SOURCE1 2536 0.0021

c2r car 1 0.14800 298905.0 ; SOUECE3 1

c2r ccr 1 0.13600 437311.7 ; SOURCE1 771 0.0185

c2r cdr 1 0.13600 437311.7 ; SOURCE1 771 0.0185

c2r cer 1 0.13390 469026.4 ; SOURCE3 62 0.0128

c2r cfr 1 0.13390 469026.4 ; SOURCE3 62 same_as_c2-ce

c2r clr 1 0.17220 275139.8 ; SOURCE1 163 0.0098

c2r cur 1 0.13320 480239.5 ; SOURCE2 1 0.0000

c2r cxr 1 0.14836 295641.4 ; SOURCE4 26 0.0064

c2r cyr 1 0.15046 277566.6 ; SOURCE4 9 0.0053

c2r fr 1 0.13400 308528.2 ; SOURCE1 34 0.0000

c2r h4r 1 0.10840 291708.5 ; SOURCE3 40 0.0058

c2r h5r 1 0.10915 282838.4 ; SOURCE4 42 0.0017

c2r har 1 0.10870 288110.2 ; SOURCE3 797 0.0046

c2r hcr 1 0.10870 288110.2 ; SOURCE3 789 0.0046

c2r hxr 1 0.10830 292963.7 ; SOURCE3 3 0.0008

c2r ir 1 0.21530 186773.8 ; SOURCE3 2 0.0000

c2r n1r 1 0.13060 456892.8 ; SOURCE3 4 0.0161

c2r n2r 1 0.12880 486264.5 ; SOURCE1 103 0.0100

c2r n3r 1 0.13400 406935.8 ; SOUECE3 1

c2r nr 1 0.14070 326770.4 ; SOURCE3 9 0.0124

c2r n4r 1 0.14820 258654.9 ; SOURCE3 5 0.0064

c2r nar 1 0.13910 344008.5 ; SOURCE3 31 0.0289

c2r ncr 1 0.13130 446014.4 ; SOURCE1 99 0.0095

c2r ndr 1 0.13130 446014.4 ; SOURCE1 99 same_as_c2-nc

c2r ner 1 0.12800 500155.4 ; SOURCE3 37 0.0110

c2r nfr 1 0.12800 500155.4 ; SOURCE3 37 same_as_c2-ne

c2r nhr 1 0.13550 387103.7 ; SOURCE3 38 0.0413

c2r nor 1 0.14457 289198.1 ; SOURCE4 7 0.0087

c2r or 1 0.12244 521828.5 ; SOURCE4 15 0.0036

c2r ohr 1 0.13330 355974.7 ; SOURCE1 53 0.0000

c2r osr 1 0.13570 328527.7 ; SOURCE1 315 0.0097

c2r p2r 1 0.16700 314553.1 ; SOURCE3 62 0.0147

c2r p3r 1 0.18340 206354.9 ; SOURCE3 5 0.0042

c2r p4r 1 0.18220 212547.2 ; SOUECE3 1

c2r p5r 1 0.18658 190957.8 ; SOURCE4 5 0.0025

c2r per 1 0.16910 297315.0 ; SOURCE3 52 0.0542

c2r pfr 1 0.16910 297315.0 ; SOURCE3 52 same_as_c2-pe

c2r s2r 1 0.16100 328946.1 ; SOURCE2 1 0.0000

c2r sr 1 0.17340 235559.2 ; SOURCE3 4 0.0034

c2r s4r 1 0.17600 220245.8 ; SOUECE3 1

c2r s6r 1 0.17600 220245.8 ; SOUECE3 1

c2r shr 1 0.17771 210873.6 ; SOURCE4 5 0.0037

c2r ssr 1 0.17360 234304.0 ; SOURCE1 209 0.0155

c3r c3r 1 0.15350 253634.1 ; SOURCE1 14664 0.0048

c3r car 1 0.15130 270704.8 ; SOURCE1 1813 0.0000

c3r ccr 1 0.14990 282252.6 ; SOURCE3 50 0.0096

c3r cdr 1 0.14990 282252.6 ; SOURCE3 50 0.0096

c3r cer 1 0.15050 277231.8 ; SOURCE3 9 0.0024

c3r cfr 1 0.15050 277231.8 ; SOURCE3 9 same_as_c3-ce

c3r clr 1 0.17860 233467.2 ; SOURCE1 267 0.0194

c3r cur 1 0.14780 300745.9 ; SOURCE1 7 0.0000

c3r cvr 1 0.14890 290871.7 ; SOURCE1 11 0.0000

c3r cxr 1 0.15140 269868.0 ; SOURCE1 712 0.0045

c3r cyr 1 0.15290 258152.8 ; SOURCE1 376 0.0000

c3r fr 1 0.13440 304427.8 ; SOURCE1 617 0.0281

c3r h1r 1 0.10930 281081.1 ; SOURCE3 2175 0.0082

c3r h2r 1 0.11000 273131.5 ; SOURCE3 66 0.0280

c3r h3r 1 0.10948 278989.1 ; SOURCE4 25 0.0026

c3r hcr 1 0.10920 282252.6 ; SOURCE3 2815 0.0059

c3r hxr 1 0.10910 283424.2 ; SOURCE3 146 0.0066

c3r ir 1 0.21620 183342.9 ; SOURCE1 15 0.0000

c3r n1r 1 0.14700 272043.7 ; SOURCE3 0

c3r n2r 1 0.14770 262587.8 ; SOURCE1 129 0.0138

c3r nr 1 0.14600 276646.1 ; SOURCE1 187 0.0079

c3r n3r 1 0.14700 268278.1 ; SOURCE1 1678 0.0017

c3r n4r 1 0.14990 245684.5 ; SOURCE1 1370 0.0000

c3r nar 1 0.14560 280077.0 ; SOURCE3 23 0.0119

c3r ncr 1 0.14560 280077.0 ; SOURCE3 9 0.0109

c3r ndr 1 0.14560 280077.0 ; SOURCE3 9 same_as_c3-nc

c3r nhr 1 0.14580 278403.4 ; SOURCE3 27 0.0085

c3r nor 1 0.15330 222086.7 ; SOURCE1 83 0.0212

c3r or 1 0.13165 376476.3 ; SOURCE4 8 0.0193

c3r ohr 1 0.14260 262838.9 ; SOURCE1 914 0.0129

c3r osr 1 0.14390 252295.2 ; SOURCE1 3123 0.0126

c3r p2r 1 0.18550 196062.2 ; SOURCE3 9 0.0125

c3r p3r 1 0.18440 201334.1 ; SOURCE3 109 0.0107

c3r p4r 1 0.18330 206857.0 ; SOURCE3 29 0.0138

c3r p5r 1 0.18130 217317.0 ; SOURCE1 84 0.0000

c3r pxr 1 0.18240 211459.4 ; SOURCE3 28 0.0098

c3r pyr 1 0.18130 217317.0 ; SOURCE3 13 0.0163

c3r sr 1 0.18450 178154.7 ; SOURCE3 4 0.0185

c3r s4r 1 0.18070 195643.8 ; SOURCE1 139 0.0023

c3r s6r 1 0.17740 212547.2 ; SOURCE1 118 0.0103

c3r shr 1 0.18220 188531.0 ; SOURCE3 12 0.0051

c3r ssr 1 0.18210 188949.4 ; SOURCE1 358 0.0075

c3r sxr 1 0.18090 194639.7 ; SOURCE3 30 0.0067

c3r syr 1 0.17820 208279.5 ; SOURCE3 31 0.0039

car car 1 0.13870 400325.1 ; SOURCE1 6228 0.0147

car ccr 1 0.14340 344510.6 ; SOURCE1 80 0.0000

car cdr 1 0.14340 344510.6 ; SOURCE1 80 0.0000

car cer 1 0.14720 306268.8 ; SOURCE1 71 0.0030

car cfr 1 0.14720 306268.8 ; SOURCE1 71 0.0030

car cgr 1 0.14380 340242.9 ; SOURCE1 71 0.0045

car chr 1 0.14380 340242.9 ; SOURCE1 71 0.0045

car clr 1 0.17290 270119.0 ; SOURCE1 704 0.0095

car cpr 1 0.13950 390032.5 ; SOURCE3 14 0.0110

car cqr 1 0.14009 382752.3 ; SOURCE4 14 0.0058

car cxr 1 0.14860 293549.4 ; SOURCE1 98 0.0118

car cyr 1 0.15135 270286.4 ; SOURCE4 8 0.0043

car fr 1 0.13440 304427.8 ; SOURCE1 205 0.0089

car h4r 1 0.10880 286938.7 ; SOURCE3 57 0.0026

car h5r 1 0.10850 290537.0 ; SOURCE3 15 0.0048

car har 1 0.10870 288110.2 ; SOURCE3 1496 0.0045

car ir 1 0.20950 211208.3 ; SOURCE1 51 0.0000

car n1r 1 0.14000 333130.1 ; SOURCE3 0

car n2r 1 0.13030 461578.9 ; SOURCE4 7 0.0058

car nr 1 0.14220 311540.6 ; SOURCE3 9 0.0098

car n4r 1 0.14650 272462.1 ; SOURCE1 23 0.0000

car nar 1 0.13500 393547.0 ; SOURCE1 150 0.0103

car nbr 1 0.13420 404258.1 ; SOURCE3 104 0.0076

car ncr 1 0.13360 412458.7 ; SOURCE1 1826 0.0020

car ndr 1 0.13360 412458.7 ; SOURCE1 1826 0.0020

car ner 1 0.14310 302754.2 ; SOURCE1 52 0.0000

car nfr 1 0.14310 302754.2 ; SOURCE1 52 0.0000

car nhr 1 0.13640 375723.2 ; SOURCE1 137 0.0085

car nor 1 0.14680 269951.7 ; SOURCE1 556 0.0000

car or 1 0.12304 510448.0 ; SOURCE4 5 0.0026

car ohr 1 0.13620 323088.5 ; SOURCE1 551 0.0000

car osr 1 0.13730 311624.3 ; SOURCE1 1092 0.0071

car p2r 1 0.18400 203342.4 ; SOUECE3 1

car p3r 1 0.18240 211459.4 ; SOURCE1 145 0.0187

car p4r 1 0.18060 221166.2 ; SOUECE3 1

car p5r 1 0.17950 227274.9 ; SOURCE1 571 0.0028

car per 1 0.18290 208865.3 ; SOURCE3 10 0.0042

car pfr 1 0.18290 208865.3 ; SOURCE3 10 0.0042

car pxr 1 0.18250 210957.3 ; SOURCE3 5 0.0168

car pyr 1 0.17999 224513.4 ; SOURCE4 5 0.0072

car sr 1 0.17390 232546.7 ; SOURCE3 2 0.0000

car s4r 1 0.17880 205183.4 ; SOURCE1 51 0.0048

car s6r 1 0.17590 220831.5 ; SOURCE1 229 0.0036

car shr 1 0.17783 210287.8 ; SOURCE4 12 0.0041

car ssr 1 0.17700 214722.9 ; SOURCE1 297 0.0041

car sxr 1 0.18252 187024.8 ; SOURCE4 24 0.0032

car syr 1 0.17840 207275.4 ; SOURCE3 13 0.0094

cr c1r 1 0.14600 317816.6 ; SOUECE3 1

cr c2r 1 0.14060 376476.3 ; SOURCE3 2 0.0370

cr cr 1 0.15500 242755.7 ; SOURCE1 31 0.0100

cr c3r 1 0.15080 274721.4 ; SOURCE1 2949 0.0060

cr car 1 0.14870 292629.0 ; SOURCE1 480 0.0055

cr ccr 1 0.14620 315808.3 ; SOURCE3 132 0.0210

ccr ccr 1 0.14290 350033.4 ; SOURCE1 740 0.0069

ccr cdr 1 0.13710 421747.2 ; SOURCE3 523 0.0217

ccr cer 1 0.14502 327523.5 ; SOURCE4 157 0.0098

ccr cfr 1 0.13605 436558.6 ; SOURCE4 27 0.0086

ccr cgr 1 0.14270 352209.1 ; SOURCE1 560 0.0000

ccr chr 1 0.14270 352209.1 ; SOURCE1 560 0.0000

ccr clr 1 0.17359 265349.3 ; SOURCE4 55 0.0078

ccr cxr 1 0.14691 309030.2 ; SOURCE4 18 0.0037

cr cdr 1 0.14620 315808.3 ; SOURCE3 132 0.0210

cr cer 1 0.14740 304427.8 ; SOURCE1 601 0.0105

cr cfr 1 0.14740 304427.8 ; SOURCE1 601 0.0105

ccr fr 1 0.13401 308444.5 ; SOURCE4 24 0.0034

cr cgr 1 0.14520 325766.2 ; SOURCE3 2 0.0000

cr chr 1 0.14520 325766.2 ; SOURCE3 2 same_as_c-cg

ccr h4r 1 0.10830 292963.7 ; SOURCE3 599 0.0037

ccr h5r 1 0.10790 297900.8 ; SOURCE3 40 0.0051

ccr har 1 0.10850 290537.0 ; SOURCE3 740 0.0039

cr clr 1 0.17660 245600.8 ; SOURCE3 6 0.0250

ccr n2r 1 0.12923 479068.0 ; SOURCE4 61 0.0067

ccr nr 1 0.13800 356476.8 ; SOURCE3 56 0.0109

ccr n4r 1 0.14930 250203.2 ; SOURCE4 7 0.0148

ccr nar 1 0.13710 367187.8 ; SOURCE3 440 0.0144

ccr ncr 1 0.13760 361162.9 ; SOURCE1 88 0.0000

ccr ndr 1 0.13350 413881.3 ; SOURCE3 203 0.0239

ccr ner 1 0.13790 357648.3 ; SOURCE4 30 0.0126

ccr nfr 1 0.12885 485427.7 ; SOURCE4 10 0.0112

ccr nhr 1 0.13640 375723.2 ; SOURCE3 6 0.0040

ccr nor 1 0.14262 307440.3 ; SOURCE4 133 0.0061

ccr ohr 1 0.13427 344594.2 ; SOURCE4 64 0.0073

ccr osr 1 0.13700 314720.5 ; SOURCE3 86 0.0192

ccr pdr 1 0.17330 266269.8 ; SOURCE3 84 0.0161

ccr shr 1 0.17681 215810.7 ; SOURCE4 8 0.0027

ccr ssr 1 0.17370 233718.2 ; SOURCE3 52 0.0194

ccr sxr 1 0.18113 193551.8 ; SOURCE4 16 0.0050

ccr syr 1 0.17872 205601.8 ; SOURCE4 33 0.0105

cr cur 1 0.14120 369363.5 ; SOURCE2 1 0.0000

cr cxr 1 0.14860 293549.4 ; SOURCE1 105 0.0000

cr cyr 1 0.15290 258152.8 ; SOURCE1 18 0.0000

cdr cdr 1 0.14290 350033.4 ; SOURCE1 740 0.0069

cdr cer 1 0.13705 422416.6 ; SOURCE4 43 0.0138

cdr cfr 1 0.14583 319490.2 ; SOURCE4 92 0.0079

cdr cgr 1 0.14270 352209.1 ; SOURCE1 560 0.0000

cdr chr 1 0.14270 352209.1 ; SOURCE1 560 0.0000

cdr clr 1 0.17356 265516.6 ; SOURCE4 11 0.0080

cdr cxr 1 0.14787 300076.5 ; SOURCE4 6 0.0029

cdr cyr 1 0.15054 276897.1 ; SOURCE4 10 0.0008

cdr h4r 1 0.10830 292963.7 ; SOURCE3 599 0.0037

cdr h5r 1 0.10790 297900.8 ; SOURCE3 40 0.0051

cdr har 1 0.10850 290537.0 ; SOURCE3 740 0.0039

cdr n2r 1 0.12897 483419.4 ; SOURCE4 20 0.0086

cdr nr 1 0.13800 356476.8 ; SOURCE3 56 0.0109

cdr nar 1 0.13710 367187.8 ; SOURCE3 440 0.0144

cdr ncr 1 0.13350 413881.3 ; SOURCE3 203 0.0239

cdr ndr 1 0.13760 361162.9 ; SOURCE1 88 0.0000

cdr ner 1 0.13017 463670.9 ; SOURCE4 13 0.0118

cdr nhr 1 0.13640 375723.2 ; SOURCE3 6 0.0040

cdr ohr 1 0.13479 338653.0 ; SOURCE4 57 0.0063

cdr osr 1 0.13700 314720.5 ; SOURCE3 86 0.0192

cdr pcr 1 0.17330 266269.8 ; SOURCE3 84 same_as_cc-pd

cdr ssr 1 0.17370 233718.2 ; SOURCE3 52 0.0194

cdr syr 1 0.17777 210622.6 ; SOURCE4 22 0.0034

cer cer 1 0.14510 326770.4 ; SOURCE1 66 0.0060

cer cfr 1 0.13380 470616.3 ; SOURCE1 543 0.0045

cer cgr 1 0.14310 347774.1 ; SOURCE1 22 0.0000

cer chr 1 0.14310 347774.1 ; SOURCE1 22 0.0000

cer clr 1 0.17671 244847.7 ; SOURCE4 24 0.0062

cer cxr 1 0.14993 282001.6 ; SOURCE4 5 0.0066

cer cyr 1 0.15135 270286.4 ; SOURCE4 17 0.0024

cer h4r 1 0.10916 282671.0 ; SOURCE4 125 0.0033

cer har 1 0.10890 285767.2 ; SOURCE3 55 0.0056

cer n1r 1 0.13090 452123.0 ; SOURCE4 10 0.0027

cer n2r 1 0.12790 501912.6 ; SOURCE1 75 0.0000

cer nr 1 0.14246 308946.6 ; SOURCE4 130 0.0066

cer nar 1 0.14207 312795.8 ; SOURCE4 5 0.0051

cer ner 1 0.14140 319490.2 ; SOURCE3 7 0.0103

cer nfr 1 0.12926 478565.9 ; SOURCE4 15 0.0042

cer nhr 1 0.13912 343757.4 ; SOURCE4 148 0.0104

cer ohr 1 0.13545 331289.1 ; SOURCE4 23 0.0100

cer osr 1 0.13745 310118.1 ; SOURCE4 39 0.0077

cer p2r 1 0.18140 216814.9 ; SOUECE3 1

cer per 1 0.18180 214639.2 ; SOURCE3 8 0.0108

cer pxr 1 0.18210 213049.3 ; SOURCE3 6 0.0046

cer pyr 1 0.17940 227860.6 ; SOURCE3 5 0.0045

cer sr 1 0.16800 271541.6 ; SOUECE3 1

cer ssr 1 0.17906 203844.5 ; SOURCE4 10 0.0064

cer sxr 1 0.17970 200581.0 ; SOURCE3 5 0.0082

cer syr 1 0.17820 208279.5 ; SOURCE3 5 0.0114

cr fr 1 0.13250 324594.7 ; SOURCE2 6 0.0147

cfr cfr 1 0.14510 326770.4 ; SOURCE1 66 0.0060

cfr cgr 1 0.14310 347774.1 ; SOURCE1 22 0.0000

cfr chr 1 0.14310 347774.1 ; SOURCE1 22 0.0000

cfr h4r 1 0.10940 279909.6 ; SOURCE4 19 0.0019

cfr har 1 0.10890 285767.2 ; SOURCE3 55 0.0056

cfr n1r 1 0.13190 436977.0 ; SOURCE3 3 0.0121

cfr n2r 1 0.12790 501912.6 ; SOURCE1 75 same_as_ce-n2

cfr nr 1 0.14303 303423.7 ; SOURCE4 6 0.0082

cfr ner 1 0.12910 481243.7 ; SOURCE4 27 0.0083

cfr nfr 1 0.14140 319490.2 ; SOURCE3 7 same_as_ce-ne

cfr nhr 1 0.13822 353966.4 ; SOURCE4 20 0.0102

cfr ohr 1 0.13408 346769.9 ; SOURCE4 14 0.0084

cfr osr 1 0.13612 324009.0 ; SOURCE4 6 0.0111

cfr p2r 1 0.18140 216814.9 ; SOUECE3 1 same_as_ce-p2

cfr pfr 1 0.18180 214639.2 ; SOURCE3 8 same_as_ce-pe

cfr pxr 1 0.18210 213049.3 ; SOURCE3 6 same_as_ce-px

cfr pyr 1 0.17940 227860.6 ; SOURCE3 5 same_as_ce-py

cfr sr 1 0.16800 271541.6 ; SOUECE3 1 same_as_ce-s

cfr sxr 1 0.17970 200581.0 ; SOURCE3 5 same_as_ce-sx

cfr syr 1 0.17820 208279.5 ; SOURCE3 5 same_as_ce-sy

cgr cgr 1 0.13770 413546.6 ; SOURCE1 42 0.0000

cgr chr 1 0.11910 794541.6 ; SOURCE1 80 0.0015

cgr n1r 1 0.11430 832365.0 ; SOURCE1 316 0.0018

cgr ner 1 0.13262 426349.6 ; SOURCE4 17 0.0009

cgr per 1 0.16210 359656.6 ; SOURCE3 11 0.2008

cr h4r 1 0.11123 259826.4 ; SOURCE4 125 0.0023

cr h5r 1 0.11053 267273.9 ; SOURCE4 42 0.0028

cr har 1 0.11010 272043.7 ; SOURCE3 53 0.0102

chr chr 1 0.13770 413546.6 ; SOURCE1 42 0.0000

chr n1r 1 0.11430 832365.0 ; SOURCE1 316 0.0018

chr nfr 1 0.13262 426349.6 ; SOURCE4 17 same_as_cg-ne

chr pfr 1 0.16210 359656.6 ; SOURCE3 11 same_as_cg-pe

cr ir 1 0.22090 166439.5 ; SOURCE3 4 0.0365

clr clr 1 0.22670 119913.4 ; SOURCE1 2 0.0395

clr cxr 1 0.17550 252546.2 ; SOURCE1 64 0.0000

clr cyr 1 0.17680 244345.6 ; SOURCE2 2 0.0070

clr fr 1 0.16480 249868.5 ; SOURCE2 2 0.0500

clr ir 1 0.25500 136816.8 ; SOURCE1 6 0.0893

clr n1r 1 0.16300 361162.9 ; SOUECE3 1

clr n2r 1 0.18190 220413.1 ; SOURCE3 6 0.1020

clr n3r 1 0.17800 243006.7 ; SOURCE4 5 0.0021

clr nr 1 0.17140 288026.6 ; SOURCE4 5 0.0005

clr n4r 1 0.17530 260328.5 ; SOURCE3 4 0.0098

clr nar 1 0.18350 211877.8 ; SOURCE3 7 0.2083

clr nhr 1 0.17630 253717.8 ; SOURCE3 1 0.0000

clr nor 1 0.18400 209283.7 ; SOURCE2 1 0.0000

clr or 1 0.14830 466599.7 ; SOURCE3 4 0.0000

clr ohr 1 0.16900 259157.0 ; SOURCE2 1 0.0000

clr osr 1 0.17300 233299.8 ; SOURCE3 4 0.0000

clr p2r 1 0.20700 182004.0 ; SOURCE3 6 0.0108

clr p3r 1 0.20080 208697.9 ; SOURCE1 111 0.0000

clr p4r 1 0.20080 208697.9 ; SOURCE1 111 0.0000

clr p5r 1 0.20080 208697.9 ; SOURCE1 111 0.0000

clr pbr 1 0.19970 213886.1 ; SOURCE1 46 0.0000

clr sr 1 0.20720 174640.2 ; SOURCE1 6 0.0000

clr s2r 1 0.21610 144515.4 ; SOURCE2 1 0.0000

clr s4r 1 0.20720 174640.2 ; SOURCE1 6 0.0000

clr s6r 1 0.20720 174640.2 ; SOURCE1 6 0.0000

clr shr 1 0.20720 174640.2 ; SOURCE1 6 0.0000

clr ssr 1 0.20720 174640.2 ; SOURCE1 6 0.0000

clr sxr 1 0.20720 174640.2 ; SOURCE1 6 0.0000

clr syr 1 0.20720 174640.2 ; SOURCE1 6 0.0000

cr n2r 1 0.14200 313465.3 ; SOUECE3 1

cr n4r 1 0.15460 213802.4 ; SOURCE3 4 0.0388

cr nr 1 0.13450 400157.8 ; SOURCE1 1235 0.0215

cr ncr 1 0.13784 358401.4 ; SOURCE4 70 0.0128

cr ndr 1 0.14064 327356.2 ; SOURCE4 54 0.0130

cr ner 1 0.13929 341916.5 ; SOURCE4 47 0.0136

cr nfr 1 0.13958 338736.6 ; SOURCE4 5 0.0122

cr nor 1 0.15400 217651.7 ; SOUECE3 1

cr or 1 0.12140 542246.4 ; SOURCE1 3682 0.0165

cr ohr 1 0.13060 390283.5 ; SOURCE1 271 0.0041

cr osr 1 0.13430 344175.8 ; SOURCE1 1044 0.0171

cr p2r 1 0.19000 175979.0 ; SOUECE3 1

cr p3r 1 0.18830 183259.2 ; SOURCE3 6 0.0129

cr p4r 1 0.18800 184598.1 ; SOUECE3 1

cr p5r 1 0.18815 183928.6 ; SOURCE4 11 0.0078

cpr cpr 1 0.14900 289951.2 ; SOURCE1 242 0.0010

cpr cqr 1 0.14282 350870.2 ; SOURCE4 7 0.0034

cr per 1 0.19110 171460.3 ; SOURCE3 3 0.0025

cr pfr 1 0.19110 171460.3 ; SOURCE3 3 same_as_c-pe

cpr nar 1 0.13840 351874.4 ; SOURCE4 7 0.0181

cpr nbr 1 0.13398 407270.6 ; SOURCE4 70 0.0062

cr pxr 1 0.19040 174305.4 ; SOURCE3 1 0.0000

cr pyr 1 0.18670 190455.7 ; SOURCE3 6 0.0199

cqr cqr 1 0.14900 289951.2 ; SOURCE1 242 0.0010

cr sr 1 0.16750 275223.5 ; SOURCE1 401 0.0128

cr s4r 1 0.18700 167694.7 ; SOUECE3 1

cr s6r 1 0.18700 167694.7 ; SOUECE3 1

cr shr 1 0.17810 208865.3 ; SOURCE3 6 0.0171

cr ssr 1 0.17620 219157.9 ; SOURCE1 20 0.0000

cr sxr 1 0.18850 161753.4 ; SOURCE3 5 0.0088

cr syr 1 0.18650 169703.0 ; SOURCE3 5 0.0085

cur cur 1 0.12940 547016.2 ; SOURCE1 10 0.0000

cur cxr 1 0.15090 273884.6 ; SOURCE1 20 0.0000

cur har 1 0.10810 295390.4 ; SOURCE2 3 0.0111

cvr cvr 1 0.13350 475386.1 ; SOURCE1 25 0.0000

cvr cyr 1 0.15130 270704.8 ; SOURCE1 50 0.0000

cvr har 1 0.10870 288110.2 ; SOURCE3 2 0.0000

cxr cvr 1 0.15080 274721.4 ; SOURCE1 2536 as

cxr cxr 1 0.14990 282252.6 ; SOURCE1 1204 0.0183

cxr cyr 1 0.15150 269031.2 ; SOURCE3 2 0.0000

cxr fr 1 0.13580 290537.0 ; SOURCE2 3 0.0050

cxr h1r 1 0.10870 288110.2 ; SOURCE3 10 0.0017

cxr h2r 1 0.10830 292963.7 ; SOURCE3 2 0.0000

cxr hcr 1 0.10860 289365.4 ; SOURCE3 44 0.0011

cxr hxr 1 0.10850 290537.0 ; SOURCE4 5 0.0002

cxr n2r 1 0.14820 258654.9 ; SOURCE3 2 0.0000

cxr n3r 1 0.14720 266688.2 ; SOURCE1 134 0.0000

cxr nr 1 0.14411 293382.1 ; SOURCE4 11 0.0092

cxr nar 1 0.14616 275307.2 ; SOURCE4 11 0.0016

cxr nhr 1 0.14541 281750.6 ; SOURCE4 83 0.0076

cxr ohr 1 0.13610 324176.3 ; SOURCE3 3 0.0018

cxr osr 1 0.14200 267859.7 ; SOURCE3 7 0.0222

cxr p3r 1 0.18670 190455.7 ; SOURCE2 1 0.0000

cxr s4r 1 0.18220 188531.0 ; SOURCE2 1 0.0000

cxr s6r 1 0.17310 237400.2 ; SOURCE2 1 0.0000

cxr ssr 1 0.18150 191794.6 ; SOURCE2 1 0.0000

cyr cyr 1 0.15540 239994.2 ; SOURCE1 742 0.0041

cyr fr 1 0.13509 297482.4 ; SOURCE4 8 0.0047

cyr h1r 1 0.10970 276478.7 ; SOURCE3 17 0.0058

cyr h2r 1 0.10931 280997.4 ; SOURCE4 80 0.0019

cyr hcr 1 0.10940 279909.6 ; SOURCE3 63 0.0014

cyr nr 1 0.14693 268863.8 ; SOURCE4 250 0.0102

cyr n3r 1 0.14840 257065.0 ; SOURCE1 21 0.0000

cyr ohr 1 0.14150 272127.4 ; SOURCE3 2 0.0000

cyr osr 1 0.14316 258236.5 ; SOURCE4 23 0.0136

cyr s6r 1 0.18514 175393.3 ; SOURCE4 9 0.0166

cyr ssr 1 0.18481 176815.8 ; SOURCE4 78 0.0080

czr nhr 1 0.13391 408191.0 ; SOURCE4 32 0.0045

fr n1r 1 0.14100 314385.8 ; SOUECE3 1

fr n2r 1 0.14440 282420.0 ; SOURCE3 5 0.0377

fr n3r 1 0.14060 318486.1 ; SOURCE1 9 0.0000

fr nr 1 0.13970 327774.6 ; SOURCE3 3 0.0112

fr n4r 1 0.13080 440826.2 ; SOURCE3 2 0.0000

fr nar 1 0.14110 313381.6 ; SOURCE3 7 0.0611

fr nhr 1 0.14260 298821.3 ; SOURCE3 3 0.0085

fr nor 1 0.14670 263089.9 ; SOURCE2 1 0.0000

fr or 1 0.13300 370033.0 ; SOUECE3 1

fr ohr 1 0.14440 255558.7 ; SOURCE3 1 0.0000

fr osr 1 0.14230 272964.2 ; SOURCE3 2 0.0000

fr p2r 1 0.15360 240412.6 ; SOURCE3 7 0.2054

fr p3r 1 0.15780 212965.6 ; SOURCE2 8 0.0103

fr p4r 1 0.15900 205852.8 ; SOUECE3 1

fr p5r 1 0.15790 212379.8 ; SOURCE1 72 0.0000

fr s2r 1 0.16430 204513.9 ; SOURCE2 1 0.0000

fr sr 1 0.16600 195225.4 ; SOUECE3 1

fr s4r 1 0.15910 236312.3 ; SOURCE2 4 0.0065

fr s6r 1 0.15560 261165.3 ; SOURCE2 5 0.0220

fr shr 1 0.16490 201166.7 ; SOURCE3 1 0.0000

fr ssr 1 0.16340 209618.4 ; SOURCE3 3 0.0156

hnr n1r 1 0.09860 380827.7 ; SOURCE2 1 0.0000

hnr n2r 1 0.10290 314218.4 ; SOURCE3 108 0.0096

hnr n3r 1 0.10180 329782.9 ; SOURCE3 157 0.0086

hnr nr 1 0.10090 343255.4 ; SOURCE3 149 0.0098

hnr n4r 1 0.10330 308779.2 ; SOURCE3 264 0.0082

hnr nar 1 0.10110 340242.9 ; SOURCE3 46 0.0107

hnr nhr 1 0.10140 335724.2 ; SOURCE3 209 0.0091

hnr nor 1 0.10230 322670.1 ; SOURCE3 1 0.0000

hor or 1 0.09810 299490.7 ; SOURCE3 1 0.0000

hor ohr 1 0.09740 309281.3 ; SOURCE3 367 0.0105

hpr p2r 1 0.13360 322251.7 ; SOURCE3 87 0.1706

hpr p3r 1 0.14090 253634.1 ; SOURCE3 101 0.0617

hpr p4r 1 0.13490 308528.2 ; SOURCE3 17 0.1577

hpr p5r 1 0.14070 255224.0 ; SOURCE3 7 0.0062

hsr sr 1 0.13530 239659.5 ; SOURCE3 1 0.0000

hsr s4r 1 0.13750 222923.5 ; SOURCE3 5 0.0004

hsr s6r 1 0.13590 234973.4 ; SOURCE3 5 0.0015

hsr shr 1 0.13370 252881.0 ; SOURCE3 98 0.0486

ir ir 1 0.29170 91378.6 ; SOURCE1 1 0.0000

ir n1r 1 0.20600 252797.3 ; SOUECE3 1

ir n2r 1 0.23040 152799.7 ; SOURCE3 6 0.1186

ir nr 1 0.20980 232881.4 ; SOURCE3 5 0.0156

ir n3r 1 0.21850 193970.2 ; SOURCE3 3 0.0437

ir n4r 1 0.21550 206354.9 ; SOURCE3 3 0.0168

ir nar 1 0.21290 217986.4 ; SOURCE3 8 0.1276

ir nhr 1 0.21500 208530.6 ; SOURCE3 1 0.0000

ir nor 1 0.22310 176564.8 ; SOURCE3 1 0.0000

ir or 1 0.19800 270955.8 ; SOUECE3 1

ir ohr 1 0.21010 207442.7 ; SOURCE3 2 0.0000

ir osr 1 0.21290 195476.5 ; SOURCE3 3 0.0146

ir p2r 1 0.26430 90541.8 ; SOURCE3 6 0.0297

ir p3r 1 0.25660 103428.5 ; SOURCE3 3 0.0016

ir p4r 1 0.23520 153134.4 ; SOURCE3 4 0.2600

ir p5r 1 0.25960 98156.6 ; SOURCE3 3 0.0143

ir sr 1 0.24300 146523.7 ; SOUECE3 1

ir s4r 1 0.28700 69287.0 ; SOUECE3 1

ir s6r 1 0.28700 69287.0 ; SOURCE3 1 0.0000

ir shr 1 0.25600 115896.8 ; SOUECE3 1

ir ssr 1 0.25710 113721.1 ; SOURCE3 3 0.0065

n1r n1r 1 0.11240 1022318.6 ; SOURCE1 19 0.0000

n1r n2r 1 0.12160 717472.3 ; SOURCE1 19 0.0000

n1r n3r 1 0.13500 448273.8 ; SOUECE3 1

n1r n4r 1 0.13600 433629.8 ; SOUECE3 1

n1r nar 1 0.13500 448273.8 ; SOUECE3 1

n1r ncr 1 0.12160 717472.3 ; SOURCE1 38 0.0000

n1r ndr 1 0.12160 717472.3 ; SOURCE1 38 0.0000

n1r ner 1 0.12520 629189.9 ; SOURCE2 1 0.0000

n1r nfr 1 0.12520 629189.9 ; SOURCE2 1 same_as_n1-ne

n1r nhr 1 0.13400 463503.5 ; SOUECE3 1

n1r nor 1 0.14000 380576.6 ; SOUECE3 1

n1r or 1 0.12770 516724.0 ; SOURCE3 5 0.0438

n1r ohr 1 0.13000 476808.6 ; SOUECE3 1

n1r osr 1 0.13100 460658.4 ; SOUECE3 1

n1r p2r 1 0.16780 300243.8 ; SOURCE3 2 0.0282

n1r p3r 1 0.16600 315222.6 ; SOUECE3 1

n1r p4r 1 0.16800 295390.4 ; SOURCE3 0

n1r p5r 1 0.15710 403923.4 ; SOURCE1 132 0.0000

n1r s2r 1 0.14490 505678.2 ; SOURCE2 2 0.0010

n1r sr 1 0.16590 275056.2 ; SOURCE3 6 0.0789

n1r s4r 1 0.16500 281834.2 ; SOUECE3 1

n1r s6r 1 0.14160 560907.0 ; SOURCE2 2 0.0000

n1r shr 1 0.16100 314720.5 ; SOUECE3 1

n1r ssr 1 0.16100 314720.5 ; SOUECE3 1

n2r n2r 1 0.12710 588019.4 ; SOURCE3 27 0.0347

n2r n3r 1 0.13290 480992.6 ; SOURCE2 1 0.0000

n2r n4r 1 0.16790 168029.4 ; SOURCE3 7 0.3138

n2r nar 1 0.13685 421663.5 ; SOURCE4 18 0.0066

n2r ncr 1 0.12550 622495.5 ; SOURCE1 13 0.0000

n2r ndr 1 0.12550 622495.5 ; SOURCE1 13 same_as_n2_nc

n2r ner 1 0.12780 573626.4 ; SOURCE3 30 0.0302

n2r nfr 1 0.12780 573626.4 ; SOURCE3 30 same_as_n2-ne

n2r nhr 1 0.13560 439403.7 ; SOURCE3 22 0.0300

n2r nor 1 0.16260 194053.9 ; SOURCE3 4 0.1933

n2r or 1 0.12090 660988.3 ; SOURCE3 20 0.0344

n2r ohr 1 0.13940 348276.2 ; SOURCE1 67 0.0000

n2r osr 1 0.14060 335138.4 ; SOURCE3 10 0.0147

n2r p2r 1 0.16050 366769.4 ; SOURCE3 35 0.0737

n2r p3r 1 0.17640 239743.2 ; SOURCE3 7 0.0374

n2r p4r 1 0.17240 265851.4 ; SOUECE3 1

n2r p5r 1 0.15990 373045.4 ; SOURCE1 7 0.0000

n2r per 1 0.15400 441746.7 ; SOURCE3 20 0.1392

n2r pfr 1 0.15400 441746.7 ; SOURCE3 20 same_as_n2-pe

n2r s2r 1 0.15120 417563.2 ; SOURCE2 1 0.0000

n2r s4r 1 0.16100 314720.5 ; SOUECE3 1

n2r sr 1 0.15410 383338.1 ; SOURCE1 37 0.0000

n2r s6r 1 0.15513 372041.3 ; SOURCE4 5 0.0011

n2r shr 1 0.17380 223090.9 ; SOURCE3 5 0.0511

n2r ssr 1 0.16560 277315.5 ; SOURCE1 36 0.0000

n3r n3r 1 0.14540 320996.5 ; SOURCE1 44 0.0000

n3r n4r 1 0.14140 363924.3 ; SOURCE1 13 0.0000

n3r nar 1 0.14200 357062.6 ; SOURCE1 68 0.0000

n3r nhr 1 0.14200 357062.6 ; SOURCE1 68 0.0000

n3r nor 1 0.14450 330117.6 ; SOURCE3 3 0.0208

n3r or 1 0.13030 471955.2 ; SOURCE3 4 0.1217

n3r ohr 1 0.13960 346016.8 ; SOURCE1 28 0.0000

n3r osr 1 0.14400 300913.3 ; SOURCE1 34 0.0315

n3r p2r 1 0.16700 306770.9 ; SOUECE3 1

n3r p3r 1 0.17300 261751.0 ; SOURCE1 40 0.0000

n3r p4r 1 0.16970 285432.5 ; SOURCE1 88 0.0000

n3r p5r 1 0.16630 312628.5 ; SOURCE1 501 0.0086

n3r pyr 1 0.17003 282922.1 ; SOURCE4 6 0.0044

n3r sr 1 0.17920 194388.6 ; SOURCE3 3 0.0178

n3r s4r 1 0.17610 210287.8 ; SOURCE3 6 0.0766

n3r s6r 1 0.16320 296059.8 ; SOURCE1 99 0.0136

n3r shr 1 0.17390 222505.1 ; SOURCE3 3 0.0154

n3r ssr 1 0.17220 232546.7 ; SOURCE3 5 0.0207

n3r syr 1 0.16964 248780.6 ; SOURCE4 226 0.0081

n4r n4r 1 0.14840 292796.3 ; SOURCE3 4 0.0089

n4r nar 1 0.14350 340577.6 ; SOURCE3 9 0.0390

n4r nhr 1 0.14660 309365.0 ; SOURCE3 5 0.0108

n4r nor 1 0.14800 296394.6 ; SOUECE3 1

n4r or 1 0.13610 387940.5 ; SOURCE3 3 0.0041

n4r ohr 1 0.14000 341581.8 ; SOURCE3 3 0.0115

n4r osr 1 0.14210 319490.2 ; SOURCE3 5 0.0249

n4r p2r 1 0.19420 155561.1 ; SOURCE3 10 0.0643

n4r p3r 1 0.18800 179995.7 ; SOURCE3 5 0.0146

n4r p4r 1 0.19380 156983.7 ; SOURCE3 1 0.0000

n4r p5r 1 0.18300 203258.7 ; SOURCE3 5 0.0087

n4r pyr 1 0.19020 170874.6 ; SOURCE3 4 0.0000

n4r sr 1 0.18320 175979.0 ; SOURCE3 3 0.0004

n4r s4r 1 0.19720 126356.8 ; SOURCE3 3 0.0198

n4r s6r 1 0.19140 144515.4 ; SOURCE3 5 0.0432

n4r shr 1 0.18110 185351.2 ; SOURCE3 3 0.0027

n4r ssr 1 0.18120 184932.8 ; SOURCE3 5 0.0064

nar nar 1 0.14010 379321.4 ; SOURCE1 40 0.0000

nar nbr 1 0.13440 457311.2 ; SOURCE4 5 0.0070

nar ncr 1 0.13500 448273.8 ; SOURCE3 152 0.0180

nar ndr 1 0.13500 448273.8 ; SOURCE3 152 0.0180

nar nhr 1 0.14010 379321.4 ; SOURCE1 40 0.0000

nar nor 1 0.14390 336309.9 ; SOURCE3 9 0.0289

nar or 1 0.12650 539150.2 ; SOURCE1 25 0.0347

nar ohr 1 0.13970 344929.0 ; SOURCE3 9 0.0217

nar osr 1 0.14440 297231.4 ; SOURCE3 45 0.0423

nar p2r 1 0.17490 249199.0 ; SOURCE3 11 0.0192

nar p3r 1 0.17620 240998.4 ; SOURCE3 8 0.0113

nar p4r 1 0.15640 412040.3 ; SOURCE3 5 0.2161

nar p5r 1 0.17150 272211.0 ; SOURCE3 11 0.0238

nar pcr 1 0.17320 260328.5 ; SOURCE3 81 0.0207

nar pdr 1 0.17320 260328.5 ; SOURCE3 81 same_as_na-pc

nar pyr 1 0.17120 274303.0 ; SOURCE3 2 0.0000

nar sr 1 0.17650 208112.2 ; SOURCE3 8 0.0095

nar s4r 1 0.17930 193886.6 ; SOURCE3 10 0.0421

nar s6r 1 0.17270 229534.2 ; SOURCE3 10 0.0201

nar shr 1 0.17210 233132.5 ; SOURCE3 9 0.0113

nar ssr 1 0.17330 226019.7 ; SOURCE3 38 0.0412

nar syr 1 0.17270 229534.2 ; SOURCE3 1

nbr nbr 1 0.13420 460407.4 ; SOURCE1 15 0.0314

nbr pbr 1 0.15870 385848.5 ; SOURCE1 162 0.0091

ncr ncr 1 0.13790 407354.2 ; SOURCE3 9 0.0164

ncr ndr 1 0.13150 504506.7 ; SOURCE3 9 0.0221

ncr osr 1 0.13950 347188.3 ; SOURCE1 46 0.0188

ncr ssr 1 0.15600 362752.8 ; SOURCE1 74 0.0000

ncr syr 1 0.15550 368024.6 ; SOURCE3 2

ndr ndr 1 0.13790 407354.2 ; SOURCE3 9 0.0164

ndr osr 1 0.13950 347188.3 ; SOURCE1 46 0.0188

ndr ssr 1 0.15600 362752.8 ; SOURCE1 74 0.0000

ndr syr 1 0.15550 368024.6 ; SOURCE3 2

ner ner 1 0.14790 297315.0 ; SOURCE3 19 0.1705

ner nfr 1 0.12635 603834.9 ; SOURCE4 25 0.0034

ner or 1 0.12280 616219.5 ; SOURCE3 40 0.0255

ner p2r 1 0.15630 413295.5 ; SOURCE3 14 0.1325

ner per 1 0.17120 274303.0 ; SOURCE3 28 0.1076

ner pxr 1 0.17020 281666.9 ; SOURCE3 11 0.0883

ner pyr 1 0.16157 355974.7 ; SOURCE4 10 0.0094

ner sr 1 0.15370 387856.8 ; SOURCE3 22 0.1708

ner sxr 1 0.18380 173468.6 ; SOURCE3 7 0.1060

ner syr 1 0.17520 215141.3 ; SOURCE3 7 0.0814

nfr nfr 1 0.14790 297315.0 ; SOURCE3 19 same_as_ne-ne

nfr or 1 0.12280 616219.5 ; SOURCE3 40 same_as_ne-o

nfr p2r 1 0.15630 413295.5 ; SOURCE3 14 same_as_ne-p2

nfr pfr 1 0.17120 274303.0 ; SOURCE3 28 same_as_ne-pe

nfr pxr 1 0.17020 281666.9 ; SOURCE3 11 same_as_ne-px

nfr pyr 1 0.16157 355974.7 ; SOURCE4 10 same_as_ne-py

nfr sr 1 0.15370 387856.8 ; SOURCE3 22 same_as_ne-s

nfr sxr 1 0.18380 173468.6 ; SOURCE3 7 same_as_ne-sx

nfr syr 1 0.17520 215141.3 ; SOURCE3 7 same_as_ne-sy

nhr nhr 1 0.14010 379321.4 ; SOURCE1 40 0.0000

nhr nor 1 0.13850 399488.3 ; SOURCE4 7 0.0036

nhr or 1 0.12870 498900.2 ; SOURCE3 3 0.0450

nhr ohr 1 0.14144 326268.3 ; SOURCE4 19 0.0064

nhr osr 1 0.14161 324511.0 ; SOURCE4 6 0.0039

nhr p2r 1 0.16790 299407.0 ; SOURCE3 17 0.0872

nhr p3r 1 0.17300 261751.0 ; SOURCE3 3 0.0016

nhr p4r 1 0.17060 278738.1 ; SOURCE3 3 0.0008

nhr p5r 1 0.16710 305934.1 ; SOURCE3 3 0.0007

nhr sr 1 0.17840 198321.6 ; SOURCE3 3 0.0076

nhr s4r 1 0.17490 216814.9 ; SOURCE3 3 0.0203

nhr s6r 1 0.16965 248697.0 ; SOURCE4 33 0.0062

nhr shr 1 0.17080 241249.4 ; SOURCE3 1 0.0000

nhr ssr 1 0.17080 241249.4 ; SOURCE1 52 0.0015

nhr syr 1 0.17144 237232.8 ; SOURCE4 80 0.0066

nr n1r 1 0.13400 463503.5 ; SOUECE3 1

nr n2r 1 0.13710 418149.0 ; SOURCE3 9 0.0200

nr n3r 1 0.14080 370953.4 ; SOURCE3 5 0.0087

nr n4r 1 0.14320 343757.4 ; SOURCE3 5 0.0098

nr nr 1 0.13900 393045.0 ; SOURCE3 5 0.0038

nr nar 1 0.13790 407354.2 ; SOURCE3 11 0.0071

nr ncr 1 0.13561 439236.3 ; SOURCE4 63 0.0104

nr ndr 1 0.13603 433211.4 ; SOURCE4 13 0.0122

nr nhr 1 0.14025 377564.2 ; SOURCE4 20 0.0074

nr nor 1 0.14560 318988.2 ; SOURCE3 4 0.0327

nr or 1 0.12640 541074.9 ; SOURCE3 9 0.0381

nr ohr 1 0.14100 330870.7 ; SOURCE3 6 0.0106

nor nor 1 0.18240 115729.4 ; SOURCE3 1 0.0000

nor or 1 0.12190 636972.2 ; SOURCE1 1838 0.0049

nor ohr 1 0.14060 335138.4 ; SOURCE2 1 0.0000

nor osr 1 0.14229 317565.6 ; SOURCE4 53 0.0076

nor osr 1 0.14229 317565.6 ; SOURCE4 53 0.0076

nor p2r 1 0.17380 256311.8 ; SOURCE3 10 0.2231

nor p3r 1 0.18440 196397.0 ; SOURCE3 3 0.0005

nor p4r 1 0.18700 184430.7 ; SOURCE3 3 0.0006

nor p5r 1 0.18340 201250.4 ; SOURCE3 4 0.0020

nor sr 1 0.17420 220747.8 ; SOURCE3 2 0.0000

nr osr 1 0.14103 330536.0 ; SOURCE4 30 0.0112

nor s4r 1 0.19960 119662.4 ; SOURCE3 3 0.0313

nor s6r 1 0.19760 125185.3 ; SOURCE3 3 0.0520

nor shr 1 0.18040 188614.7 ; SOURCE3 1 0.0000

nor ssr 1 0.18280 177736.3 ; SOURCE3 3 0.0244

nr p2r 1 0.17330 259659.0 ; SOURCE3 8 0.0217

nr p3r 1 0.17700 236145.0 ; SOURCE3 9 0.0118

nr p4r 1 0.17340 258989.6 ; SOURCE3 1 0.0000

nr p5r 1 0.17080 277231.8 ; SOURCE4 6 0.0022

nr pcr 1 0.17400 255056.6 ; SOURCE3 3 0.0010

nr pdr 1 0.17400 255056.6 ; SOURCE3 3 same_as_n-pc

nr sr 1 0.17670 207108.0 ; SOURCE3 3 0.0011

nr s4r 1 0.17820 199325.8 ; SOURCE3 4 0.0214

nr s6r 1 0.17151 236814.4 ; SOURCE4 13 0.0138

nr shr 1 0.17280 228948.5 ; SOURCE3 4 0.0128

nr ssr 1 0.17170 235642.9 ; SOURCE3 7 0.0133

nr syr 1 0.17152 236730.7 ; SOURCE4 51 0.0079

ohr ohr 1 0.14690 284930.4 ; SOURCE3 1 0.0000

ohr osr 1 0.14547 297733.4 ; SOURCE4 19 0.0050

ohr p2r 1 0.16300 265098.2 ; SOURCE3 8 0.0916

ohr p3r 1 0.16770 233299.8 ; SOURCE3 3 0.0148

ohr p4r 1 0.16410 257232.3 ; SOURCE3 4 0.0092

ohr p5r 1 0.16250 268780.2 ; SOURCE3 92 0.0451

ohr pyr 1 0.16130 277901.3 ; SOURCE3 79 0.0138

ohr sr 1 0.18120 158992.0 ; SOURCE3 2 0.0000

ohr s4r 1 0.16954 214471.8 ; SOURCE4 10 0.0091

ohr s6r 1 0.15880 287942.9 ; SOURCE3 13 0.0091

ohr shr 1 0.16920 216396.5 ; SOURCE3 2 0.0003

ohr ssr 1 0.16820 222254.1 ; SOURCE3 4 0.0131

ohr syr 1 0.16490 243006.7 ; SOURCE4 33 0.0044

or or 1 0.14300 321582.2 ; SOURCE3 2 0.0500

or ohr 1 0.15170 246521.3 ; SOURCE3 2 0.0000

or osr 1 0.15040 256311.8 ; SOURCE3 3 0.0117

or p2r 1 0.15080 376309.0 ; SOURCE3 17 0.0306

or p3r 1 0.15150 368526.7 ; SOURCE3 35 0.0297

or p4r 1 0.15030 381915.5 ; SOURCE3 42 0.0749

or p5r 1 0.14810 408107.4 ; SOURCE1 263 0.0205

or per 1 0.15210 361999.7 ; SOURCE3 20 0.0171

or pfr 1 0.15210 361999.7 ; SOURCE3 20 same_as_o-pe

or pxr 1 0.15010 384258.6 ; SOURCE3 37 0.0160

or pyr 1 0.14880 399572.0 ; SOURCE3 63 0.0091

or sr 1 0.18020 163008.6 ; SOURCE3 2 0.0000

or s2r 1 0.15990 279156.5 ; SOURCE3 3 0.0707

or s4r 1 0.14970 375472.2 ; SOURCE1 90 0.0000

or s6r 1 0.14360 452792.5 ; SOURCE1 1038 0.0128

or shr 1 0.16050 274470.4 ; SOURCE3 2 0.0000

osr osr 1 0.14660 287524.5 ; SOURCE1 20 0.0067

osr p2r 1 0.15730 311205.9 ; SOURCE1 16 0.0000

osr p3r 1 0.16860 227777.0 ; SOURCE3 6 0.0201

osr p4r 1 0.16360 260746.9 ; SOURCE3 4 0.0057

osr p5r 1 0.16020 286604.0 ; SOURCE1 248 0.0400

OS p5r 1 0.16020 286604.0 ; SOURCE1 248 0.0400

osr pyr 1 0.16170 274888.8 ; SOURCE3 17 0.0139

osr sr 1 0.18000 163845.4 ; SOURCE3 3 0.0052

or ssr 1 0.15370 333464.8 ; SOURCE3 3 0.0501

osr s4r 1 0.16990 212463.5 ; SOURCE3 8 0.0223

osr s6r 1 0.15770 297064.0 ; SOURCE1 75 0.0030

osr shr 1 0.16710 228948.5 ; SOURCE3 3 0.0106

osr ssr 1 0.17040 209618.4 ; SOURCE3 9 0.0277

osr syr 1 0.16990 212463.5 ; SOURCE3 1 0.0000

or sxr 1 0.15080 363338.6 ; SOURCE3 40 0.0130

or syr 1 0.14660 412542.4 ; SOURCE3 92 0.0114

p2r p2r 1 0.17860 410283.0 ; SOURCE3 25 0.3488

p2r p3r 1 0.21520 177317.9 ; SOURCE3 9 0.1777

p2r p4r 1 0.21790 167694.7 ; SOUECE3 1

p2r p5r 1 0.21800 167276.3 ; SOUECE3 1

p2r per 1 0.18670 336058.9 ; SOURCE3 16 0.3571

p2r pfr 1 0.18670 336058.9 ; SOURCE3 16 same_as_p2-pe

p2r sr 1 0.17720 302586.9 ; SOURCE3 26 0.3014

p2r s4r 1 0.21900 116649.9 ; SOUECE3 1

p2r s6r 1 0.21800 119076.6 ; SOUECE3 1

p2r shr 1 0.19710 187443.2 ; SOURCE3 10 0.2829

p2r ssr 1 0.19660 189618.9 ; SOURCE3 10 0.2739

p3r p3r 1 0.22140 156063.2 ; SOURCE1 41 0.0000

p3r p4r 1 0.22160 155393.8 ; SOURCE3 3 0.0011

p3r p5r 1 0.22130 156397.9 ; SOURCE3 9 0.0265

p3r sr 1 0.20700 150373.0 ; SOUECE3 1

p3r s4r 1 0.20870 144933.8 ; SOURCE3 8 0.2235

p3r s6r 1 0.20770 148029.9 ; SOURCE3 11 0.1420

p3r shr 1 0.21320 131628.6 ; SOURCE3 3 0.0078

p3r ssr 1 0.21210 134724.8 ; SOURCE3 3 0.0059

p4r p4r 1 0.20340 228530.1 ; SOURCE1 1 0.0000

p4r p5r 1 0.22370 148950.4 ; SOUECE3 1

p4r sr 1 0.21460 127779.4 ; SOURCE3 5 0.0601

p4r s4r 1 0.22510 103093.8 ; SOUECE3 1

p4r s6r 1 0.22690 99495.5 ; SOUECE3 1

p4r shr 1 0.21150 136482.1 ; SOURCE3 4 0.0008

p4r ssr 1 0.21040 139745.6 ; SOURCE3 4 0.0044

p5r p5r 1 0.20540 218739.5 ; SOURCE1 1 0.0000

p5r sr 1 0.19220 209869.4 ; SOURCE1 89 0.0140

p5r s4r 1 0.20400 160581.9 ; SOUECE3 1

p5r s6r 1 0.20400 160581.9 ; SOUECE3 1

p5r shr 1 0.20820 146440.0 ; SOURCE3 3 0.0035

p5r ssr 1 0.21149 136482.1 ; SOURCE4 24 0.0106

per per 1 0.20920 201417.8 ; SOURCE3 7 0.1369

per pfr 1 0.20550 218237.4 ; SOURCE3 1 0.0000

per pxr 1 0.20050 243843.5 ; SOURCE3 12 0.2609

per pyr 1 0.20250 233132.5 ; SOURCE3 12 0.2617

per sr 1 0.17580 313549.0 ; SOURCE3 31 0.3197

per sxr 1 0.21680 122089.1 ; SOURCE3 9 0.1743

per syr 1 0.22130 111294.4 ; SOURCE3 6 0.0127

pfr pfr 1 0.20920 201417.8 ; SOURCE3 7 same_as_pe-pe

pfr pxr 1 0.20050 243843.5 ; SOURCE3 12 same_as_pe-px

pfr pyr 1 0.20250 233132.5 ; SOURCE3 12 same_as_pe-py

pfr sr 1 0.17580 313549.0 ; SOURCE3 31 same_as_pe-s

pfr sxr 1 0.21680 122089.1 ; SOURCE3 9 same_as_pe-sx

pfr syr 1 0.22130 111294.4 ; SOURCE3 6 same_as_pe-sy

pxr pyr 1 0.21990 160916.6 ; SOURCE3 5 0.0238

pxr sxr 1 0.22420 104934.7 ; SOURCE3 3 0.0119

pxr syr 1 0.22490 103512.2 ; SOURCE3 3 0.0272

pyr pyr 1 0.21860 165268.0 ; SOURCE3 8 0.0132

pyr sxr 1 0.22590 101420.2 ; SOURCE3 7 0.0603

pyr syr 1 0.21820 118574.6 ; SOURCE3 5 0.0047

s4r s4r 1 0.20800 126775.2 ; SOUECE3 1

s4r s6r 1 0.20800 126775.2 ; SOUECE3 1

s4r shr 1 0.21680 105185.8 ; SOURCE3 3 0.0227

s4r ssr 1 0.21660 105604.2 ; SOURCE3 5 0.0247

s6r s6r 1 0.20800 126775.2 ; SOUECE3 1

s6r shr 1 0.21080 119327.7 ; SOURCE3 3 0.0144

s6r ssr 1 0.21180 116817.3 ; SOURCE3 5 0.0209

shr shr 1 0.20580 132967.5 ; SOURCE2 1 0.0000

shr ssr 1 0.20670 130373.4 ; SOURCE3 3 0.0029

sr sr 1 0.20300 141419.2 ; SOURCE3 1 0.0000

sr s2r 1 0.18970 191794.6 ; SOURCE1 5 0.0000

sr s4r 1 0.20760 127863.0 ; SOURCE3 4 0.0345

sr s6r 1 0.20380 138908.8 ; SOURCE3 3 0.0311

sr shr 1 0.21100 118825.6 ; SOURCE3 2 0.0000

sr ssr 1 0.20890 124264.8 ; SOURCE3 1 0.0000

ssr ssr 1 0.20500 135310.6 ; SOURCE1 225 0.0015

sxr sxr 1 0.23910 67697.1 ; SOURCE3 3 0.0185

sxr syr 1 0.22550 88115.0 ; SOURCE3 5 0.0737

syr syr 1 0.22500 89035.5 ; SOURCE3 3 0.0289

**Force Field Non-Bonded Parameters (ffnonbonded.itp)**

[ atomtypes ]

; name at.num mass charge ptype sigma epsilon

h1r 1 1.008 0.0000 A 2.47135e-01 6.56888e-02

h2r 1 1.008 0.0000 A 2.29317e-01 6.56888e-02

h3r 1 1.008 0.0000 A 2.11499e-01 6.56888e-02

h4r 1 1.008 0.0000 A 2.51055e-01 6.27600e-02

h5r 1 1.008 0.0000 A 2.42146e-01 6.27600e-02

har 1 1.008 0.0000 A 2.59964e-01 6.27600e-02

hcr 1 1.008 0.0000 A 2.64953e-01 6.56888e-02

hnr 1 1.008 0.0000 A 1.06908e-01 6.56888e-02

hor 1 1.008 0.0000 A 0.00000e+00 0.00000e+00

hpr 1 1.008 0.0000 A 1.06908e-01 6.56888e-02

hsr 1 1.008 0.0000 A 1.06908e-01 6.56888e-02

hwr 1 1.008 0.0000 A 0.00000e+00 0.00000e+00

hxr 1 1.008 0.0000 A 1.95998e-01 6.56888e-02

or 8 16.00 0.0000 A 2.95957e-01 8.78640e-01

ohr 8 16.00 0.0000 A 3.06647e-01 8.80314e-01

osr 8 16.00 0.0000 A 2.99877e-01 7.11280e-01

owr 8 16.00 0.0000 A 3.15022e-01 6.35968e-01

cr 6 12.01 0.0000 A 3.39967e-01 3.59824e-01

c1r 6 12.01 0.0000 A 3.39967e-01 8.78640e-01

c2r 6 12.01 0.0000 A 3.39967e-01 3.59824e-01

c3r 6 12.01 0.0000 A 3.39967e-01 4.57730e-01

car 6 12.01 0.0000 A 3.39967e-01 3.59824e-01

cbr 6 12.01 0.0000 A 3.39967e-01 3.59824e-01

ccr 6 12.01 0.0000 A 3.39967e-01 3.59824e-01

cdr 6 12.01 0.0000 A 3.39967e-01 3.59824e-01

cer 6 12.01 0.0000 A 3.39967e-01 3.59824e-01

cfr 6 12.01 0.0000 A 3.39967e-01 3.59824e-01

cgr 6 12.01 0.0000 A 3.39967e-01 8.78640e-01

chr 6 12.01 0.0000 A 3.39967e-01 8.78640e-01

cpr 6 12.01 0.0000 A 3.39967e-01 3.59824e-01

cqr 6 12.01 0.0000 A 3.39967e-01 3.59824e-01

cur 6 12.01 0.0000 A 3.39967e-01 3.59824e-01

cvr 6 12.01 0.0000 A 3.39967e-01 3.59824e-01

cxr 6 12.01 0.0000 A 3.39967e-01 3.59824e-01

cyr 6 12.01 0.0000 A 3.39967e-01 3.59824e-01

czr 6 12.01 0.0000 A 3.39967e-01 3.59824e-01

nr 7 14.01 0.0000 A 3.25000e-01 7.11280e-01

n1r 7 14.01 0.0000 A 3.25000e-01 7.11280e-01

n2r 7 14.01 0.0000 A 3.25000e-01 7.11280e-01

n3r 7 14.01 0.0000 A 3.25000e-01 7.11280e-01

n4r 7 14.01 0.0000 A 3.25000e-01 7.11280e-01

nar 7 14.01 0.0000 A 3.25000e-01 7.11280e-01

nbr 7 14.01 0.0000 A 3.25000e-01 7.11280e-01

ncr 7 14.01 0.0000 A 3.25000e-01 7.11280e-01

ndr 7 14.01 0.0000 A 3.25000e-01 7.11280e-01

ner 7 14.01 0.0000 A 3.25000e-01 7.11280e-01

nfr 7 14.01 0.0000 A 3.25000e-01 7.11280e-01

nhr 7 14.01 0.0000 A 3.25000e-01 7.11280e-01

nor 7 14.01 0.0000 A 3.25000e-01 7.11280e-01

sr 16 32.06 0.0000 A 3.56359e-01 1.04600e+00

s2r 16 32.06 0.0000 A 3.56359e-01 1.04600e+00

s4r 16 32.06 0.0000 A 3.56359e-01 1.04600e+00

s6r 16 32.06 0.0000 A 3.56359e-01 1.04600e+00

sxr 16 32.06 0.0000 A 3.56359e-01 1.04600e+00

syr 16 32.06 0.0000 A 3.56359e-01 1.04600e+00

shr 16 32.06 0.0000 A 3.56359e-01 1.04600e+00

ssr 16 32.06 0.0000 A 3.56359e-01 1.04600e+00

p2r 15 30.97 0.0000 A 3.74177e-01 8.36800e-01

p3r 15 30.97 0.0000 A 3.74177e-01 8.36800e-01

p4r 15 30.97 0.0000 A 3.74177e-01 8.36800e-01

p5r 15 30.97 0.0000 A 3.74177e-01 8.36800e-01

pbr 15 30.97 0.0000 A 3.74177e-01 8.36800e-01

pcr 15 30.97 0.0000 A 3.74177e-01 8.36800e-01

pdr 15 30.97 0.0000 A 3.74177e-01 8.36800e-01

per 15 30.97 0.0000 A 3.74177e-01 8.36800e-01

pfr 15 30.97 0.0000 A 3.74177e-01 8.36800e-01

pxr 15 30.97 0.0000 A 3.74177e-01 8.36800e-01

pyr 15 30.97 0.0000 A 3.74177e-01 8.36800e-01

fr 9 19.00 0.0000 A 3.11815e-01 2.55224e-01

clr 17 35.45 0.0000 A 3.47094e-01 1.10876e+00

brr 35 79.90 0.0000 A 3.59923e-01 1.75728e+00

ir 53 126.9 0.0000 A 3.83086e-01 2.09200e+00

**References**

1. Wang JM, Wolf RM, Caldwell JW, Kollman P a, Case D a. Development and testing of a general amber force field. J Comput Chem. 2004;25:1157–74.

2. Lindorff-Larsen K, Piana S, Palmo K, Maragakis P, Klepeis JL, Dror RO, et al. Improved side-chain torsion potentials for the Amber ff99SB protein force field. Proteins Struct Funct Bioinforma. 2010 Jun;78(8):1950–8.

3. Bayly CICCI, Cieplak P, Cornell WD, Kollman P a. A well-behaved electrostatic potential based method using charge restraints for deriving atomic charges: the RESP model. J Phys …. 1993 Oct;97(40):10269–80.

4. Dupradeau F-Y, Pigache A, Zaffran T, Savineau C, Lelong R, Grivel N, et al. The R.E.D. tools: advances in RESP and ESP charge derivation and force field library building. Phys Chem Chem Phys. 2010 Jul 28;12(28):7821–39.

5. Vanquelef E, Simon S, Marquant G, Garcia E, Klimerak G, Delepine JC, et al. R.E.D. Server: A web service for deriving RESP and ESP charges and building force field libraries for new molecules and molecular fragments. Nucleic Acids Res. 2011;39(May):511–7.

6. Paramo T, East A, Garzón D, Ulmschneider MB, Bond PJ. Efficient Characterization of Protein Cavities within Molecular Simulation Trajectories: trj_cavity. J Chem Theory Comput. 2014 May 13;10(5):2151–64.

7. Huang B. MetaPocket: a meta approach to improve protein ligand binding site prediction. OMICS. 2009 Aug;13(4):325–30.

8. Menke M, Berger B, Cowen L. Matt: local flexibility aids protein multiple structure alignment. PLoS Comput Biol. 2008 Jan;4(1):e10.
